# Supplementary material for: Epigenomic analyses identify FOXM1 as a key regulator of anti-tumor immune response in esophageal adenocarcinoma
Source: Cell Death Dis. 2024 Feb 19;15(2):152. doi: 10.1038/s41419-024-06488-x (PMC10876663; doi:10.1038/s41419-024-06488-x)
Supplement: Supplementary file 1 — Supplemental Figure Legends [file 41419_2024_6488_MOESM1_ESM.docx]

**Supplementary Figure Legends**

**Figure S1.** **Figure S1**. Heatmaps of ChIP-seq signals at FOXM1 and H3K27ac peak regions, as well as TCGA DNA methylation HM450K data (±1.5Kb of peak center). Lines, peaks; color scale of peak intensity shown at the bottom. White color denotes regions without array probes.

**Figure S2.**Bar graphs showing the baseline gene expression of FOXM1 in EAC cells using (**A**) qPCR and (**B**) RNA-seq data. Bars are shown as the means ± SD for 2 replicates and as the mean for 1 replicate. (**C**) Bar graph showing the relative expression of FOXM1 upon 48-hour treatment with siRNA for FOXM1. Shown are the means ± SD from three technical replicates. ****P<0.0001; P-values were determined using an ordinary one-way ANOVA. (**D**) Western blot detection of FOXM1 in EAC cell lines upon 48-hour treatment with siRNA for FOXM1. Blot shown is representative of one replicate. (**E**) Bar graphs showing relative expression of FOXM1 upon FOXM1 knockdown cells with two different shRNA targets. Shown are the means ± SD from three technical replicates. ****P<0.0001; P-values were determined using an ordinary one-way ANOVA. (**F**) Western blot detection of FOXM1 in EAC cell lines upon FOXM1 knockdown cells with two different shRNA targets. Blot shown is representative of two replicates. (**G**) Approach for targeting FOXM1 using CRISPR Cas9. A guide RNA specific for exon 2 of FOXM1 was designed to create a deletion of a single base, causing a frameshift mutation. **(H**) Sanger sequencing showing the effects of the CRISPR Cas9 single base deletion for the guide RNA upon induction using doxycycline in the EAC cell line SKGT4. (**I**) Western blot detection of FOXM1 in the EAC cell line SKGT4 upon induction with doxycycline. Blot shown is representative of one replicate.

**Figure S3.** Representative images of colony formation upon (**A**) treatment with siRNA for FOXM1 and (**B**) FOXM1 knockdown cells with two different shRNA targets. Images shown are representative of three replicates.

**Figure S4**: *Ex vivo* coculture viability in the presence or absence of OVA-pulsed gastric cancer cells. YTN cells were either pulsed or non-pulsed with OVA peptide and co-cultured with OTI CD8+ T cells. Bar graphs show relative viability of YTN5 and YTN16 cells. Bars show the means ± SD representative of four technical replicates. **P<0.01, ***P<0.001, ****P<0.0001; P-values were determined using a one-way ANOVA with multiple comparisons.
