# Supplementary figures and images for: Epigenomic analyses identify FOXM1 as a key regulator of anti-tumor immune response in esophageal adenocarcinoma
Source: Cell Death Dis. 2024 Feb 19;15(2):152. doi: 10.1038/s41419-024-06488-x (PMC10876663; doi:10.1038/s41419-024-06488-x)

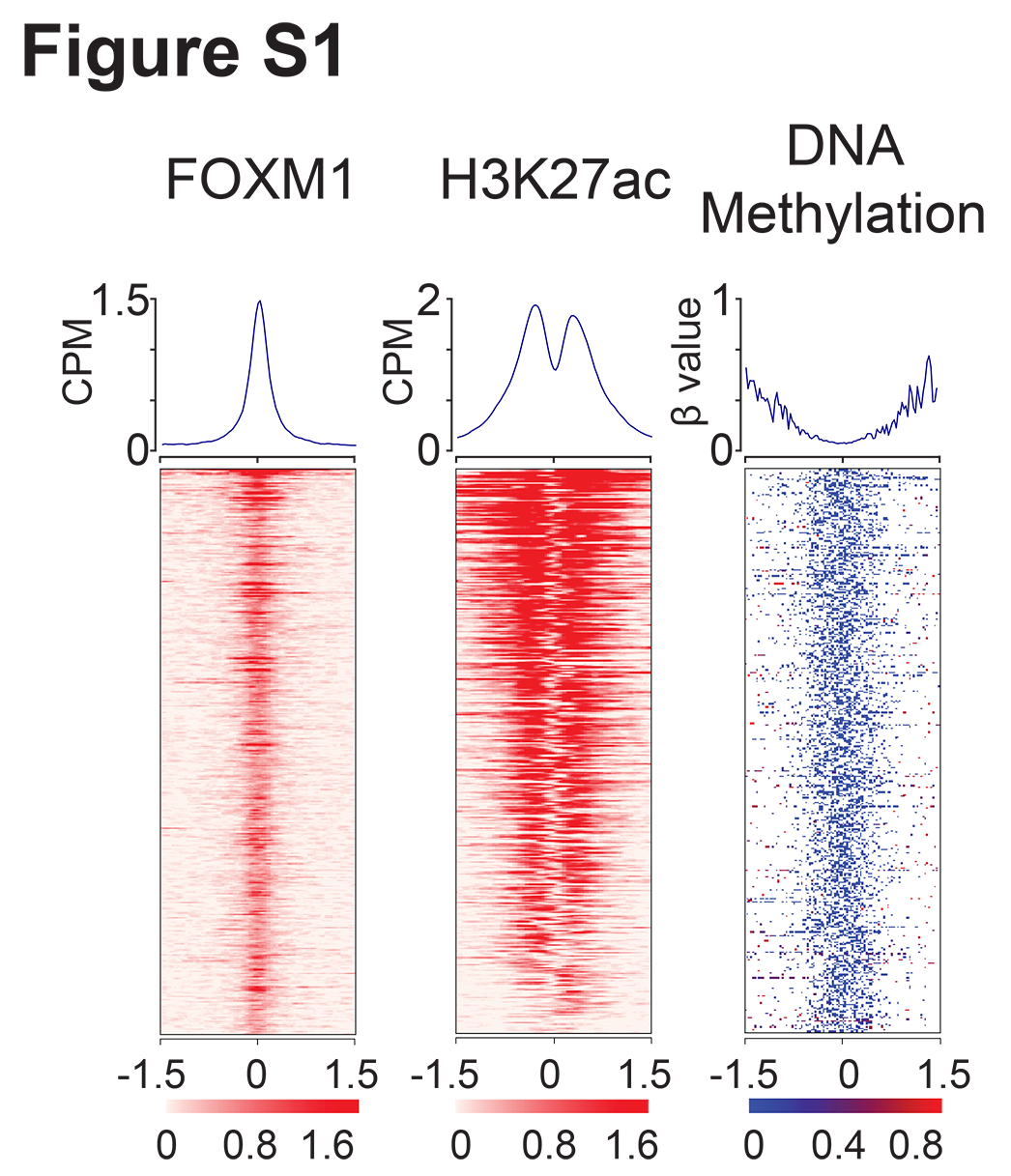

Supplement: Supplementary file 2 — Figure S1 [file 41419_2024_6488_MOESM2_ESM.tif]

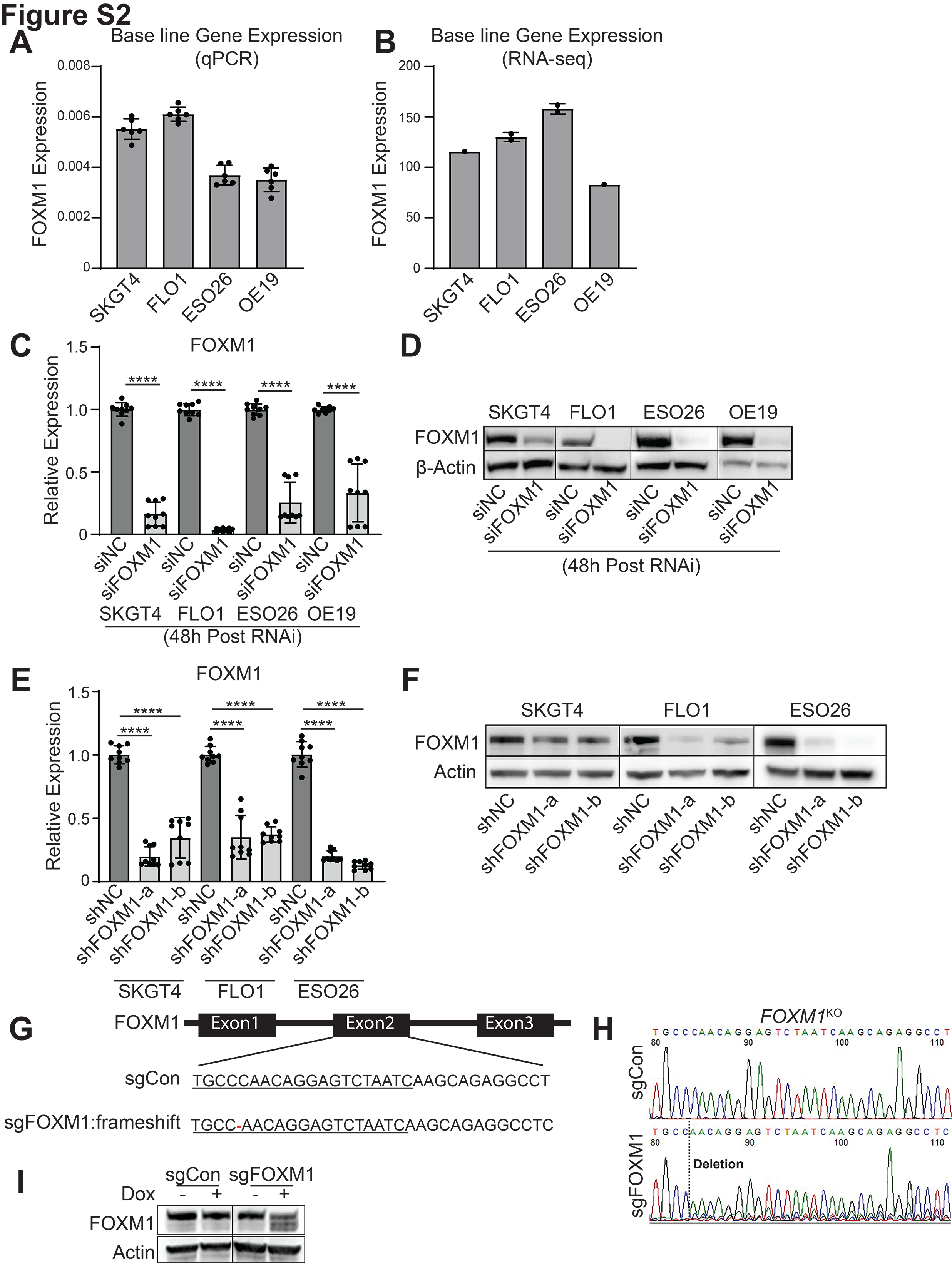

Supplement: Supplementary file 3 — Figure S2 [file 41419_2024_6488_MOESM3_ESM.tif]

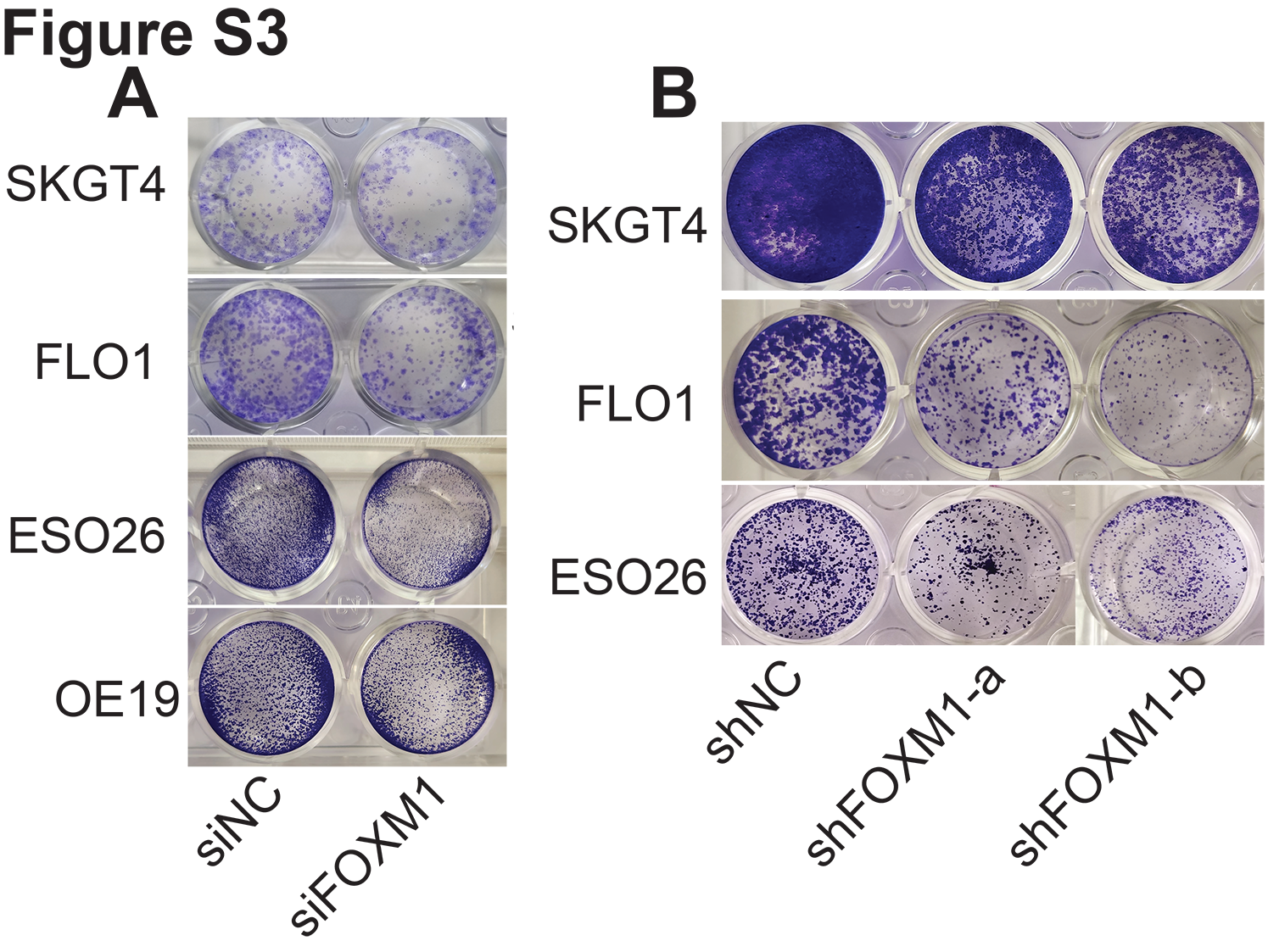

Supplement: Supplementary file 4 — Figure S3 [file 41419_2024_6488_MOESM4_ESM.tif]

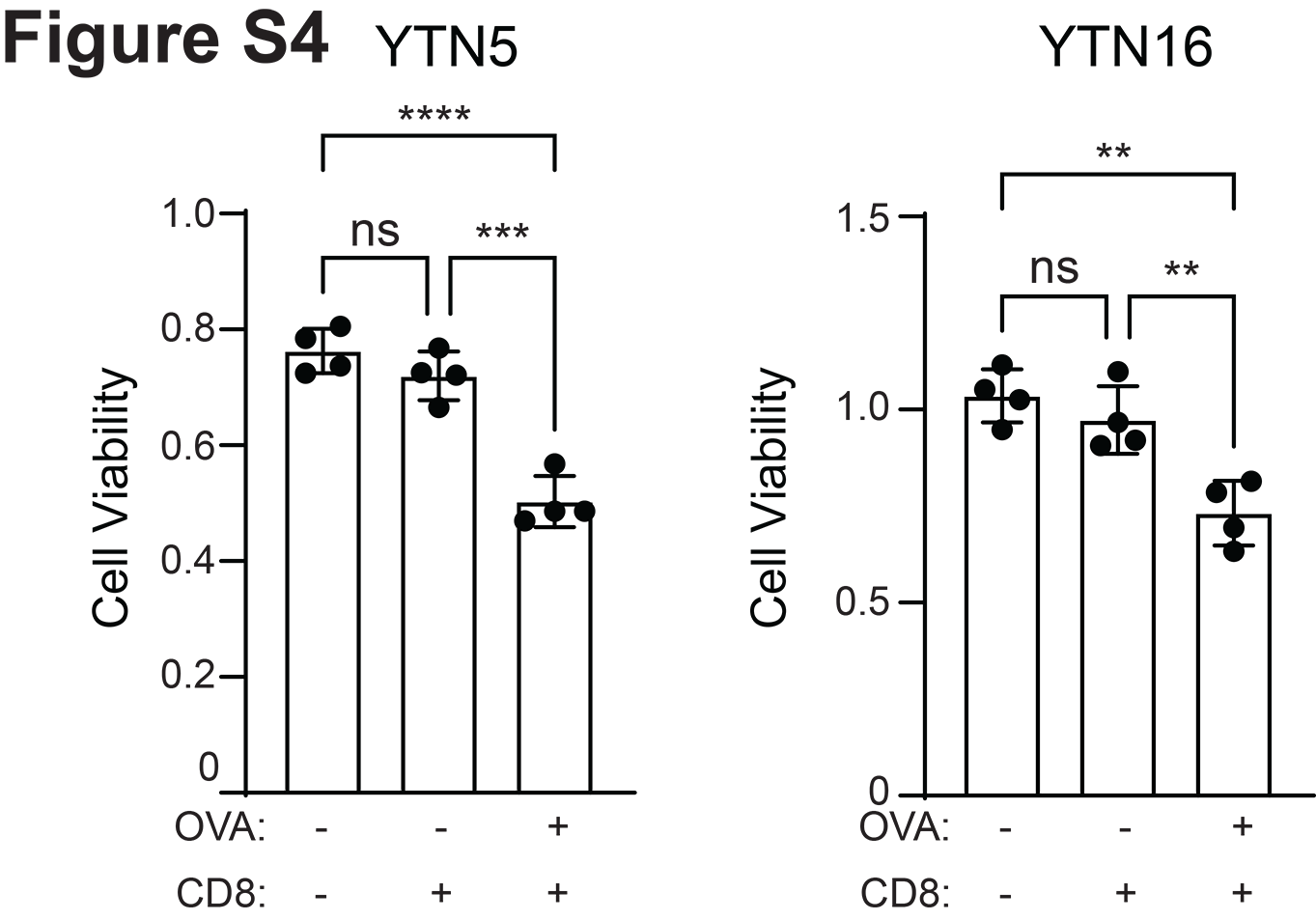

Supplement: Supplementary file 5 — Figure S4 [file 41419_2024_6488_MOESM5_ESM.tif]

CRISPR

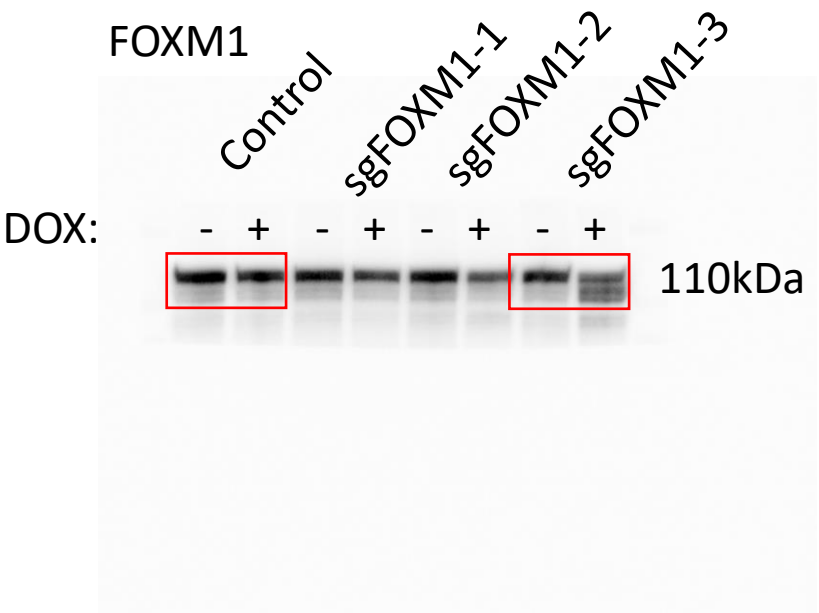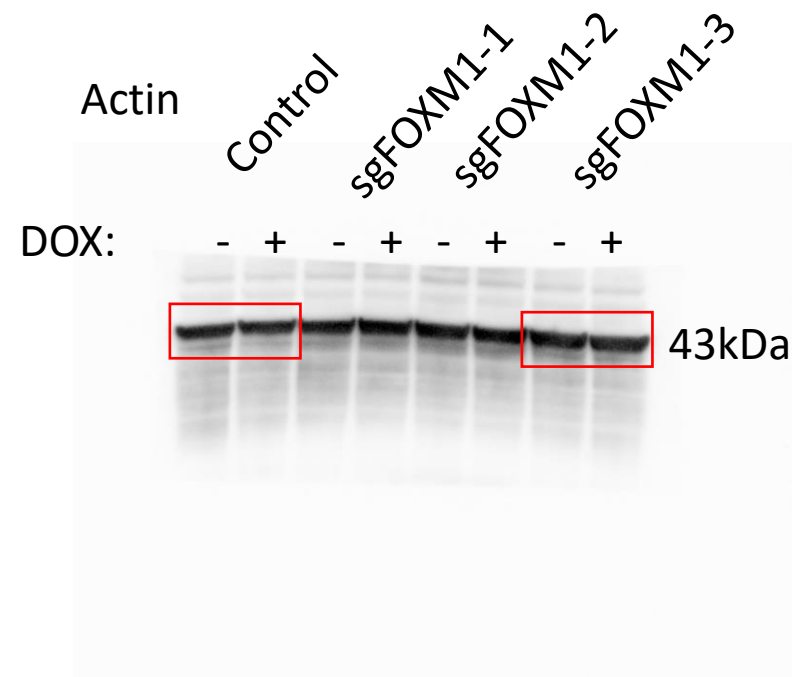

shFOXM1

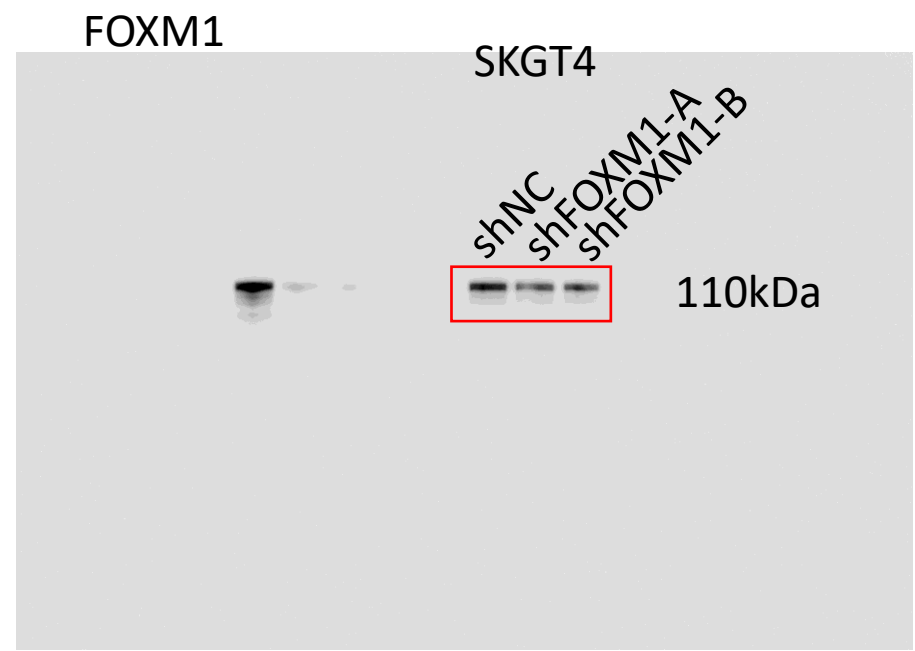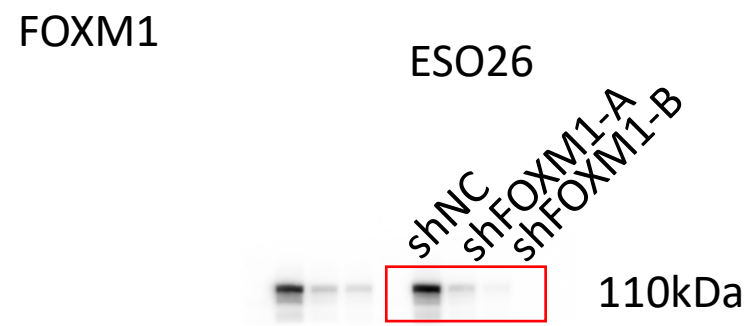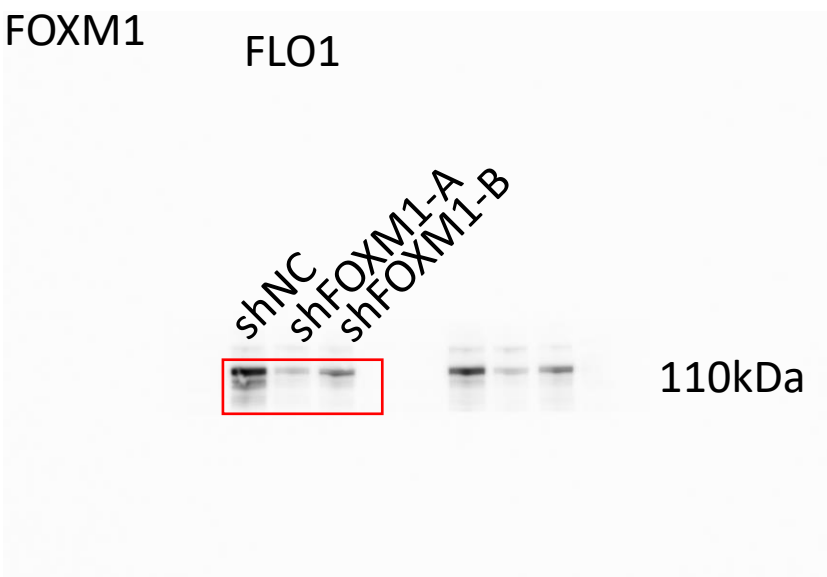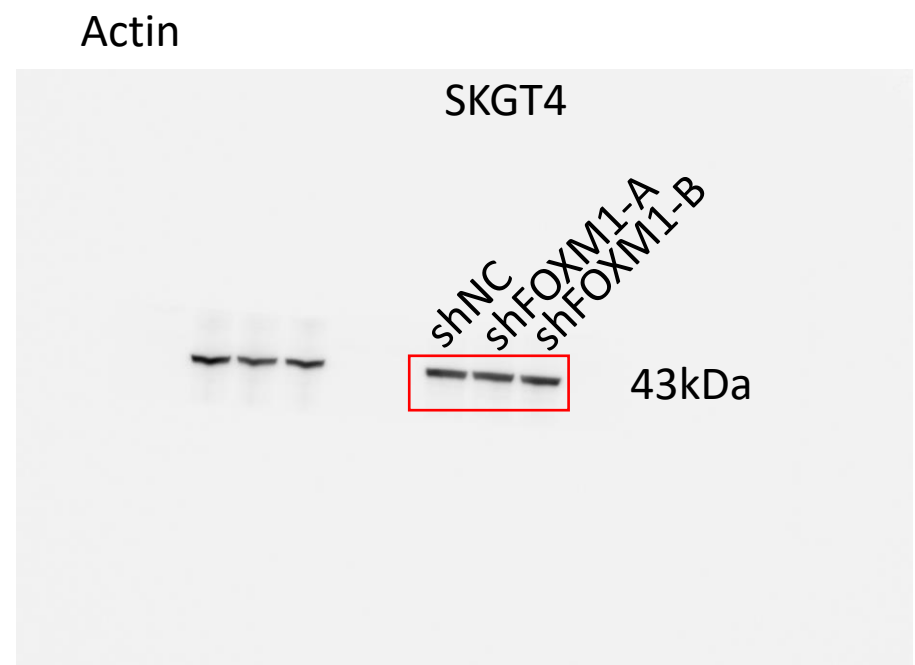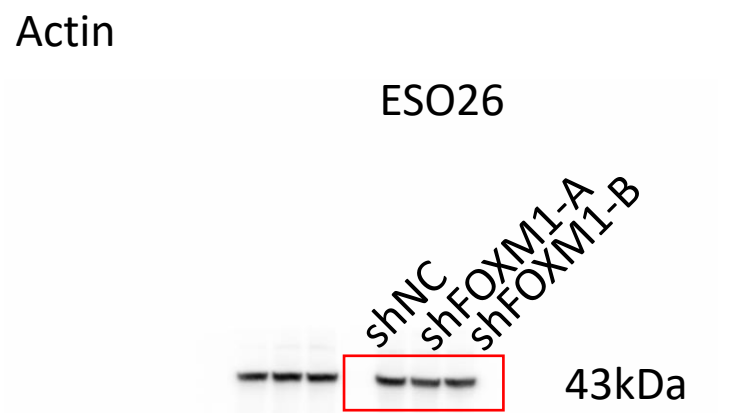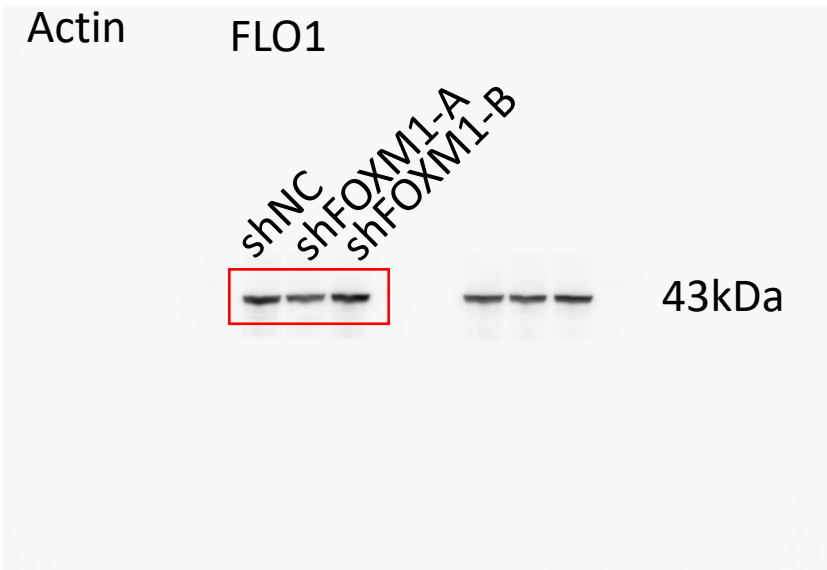

siFOXM1

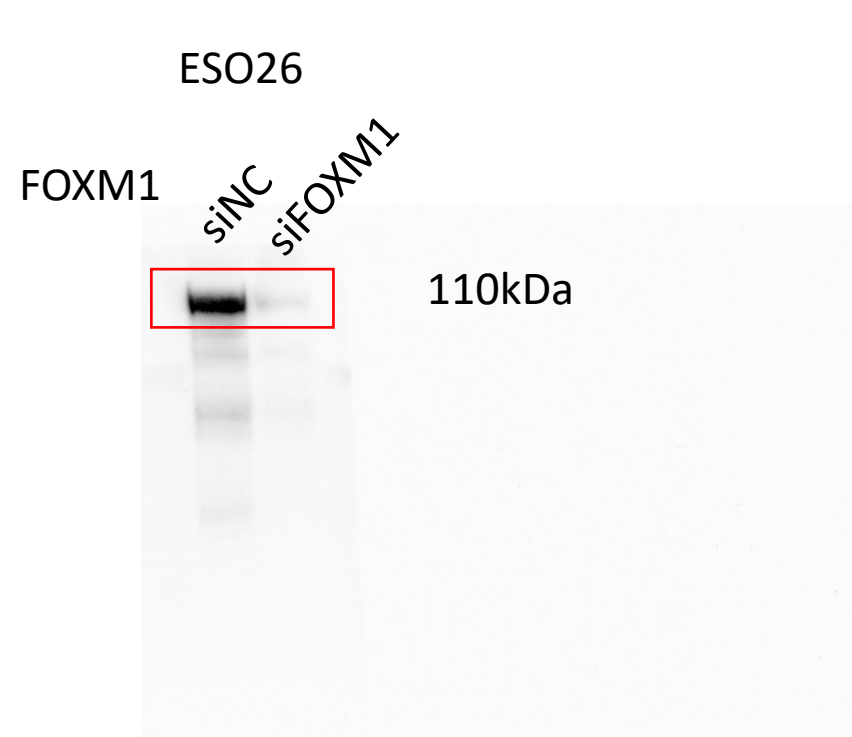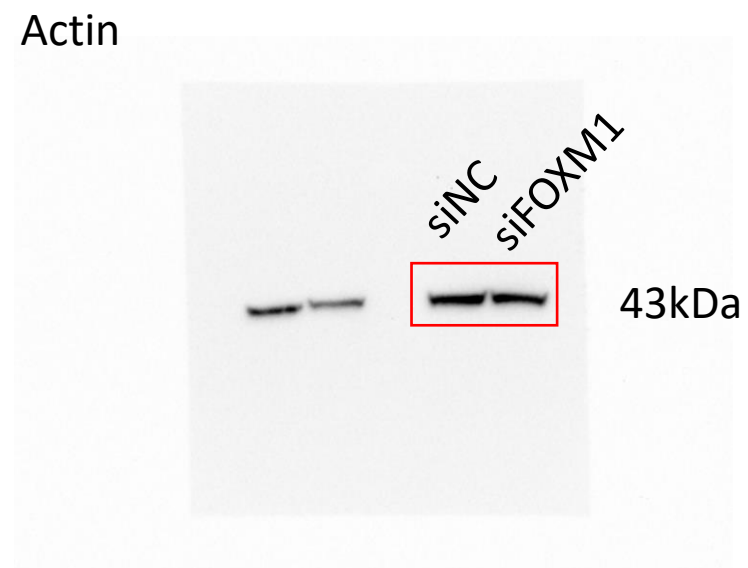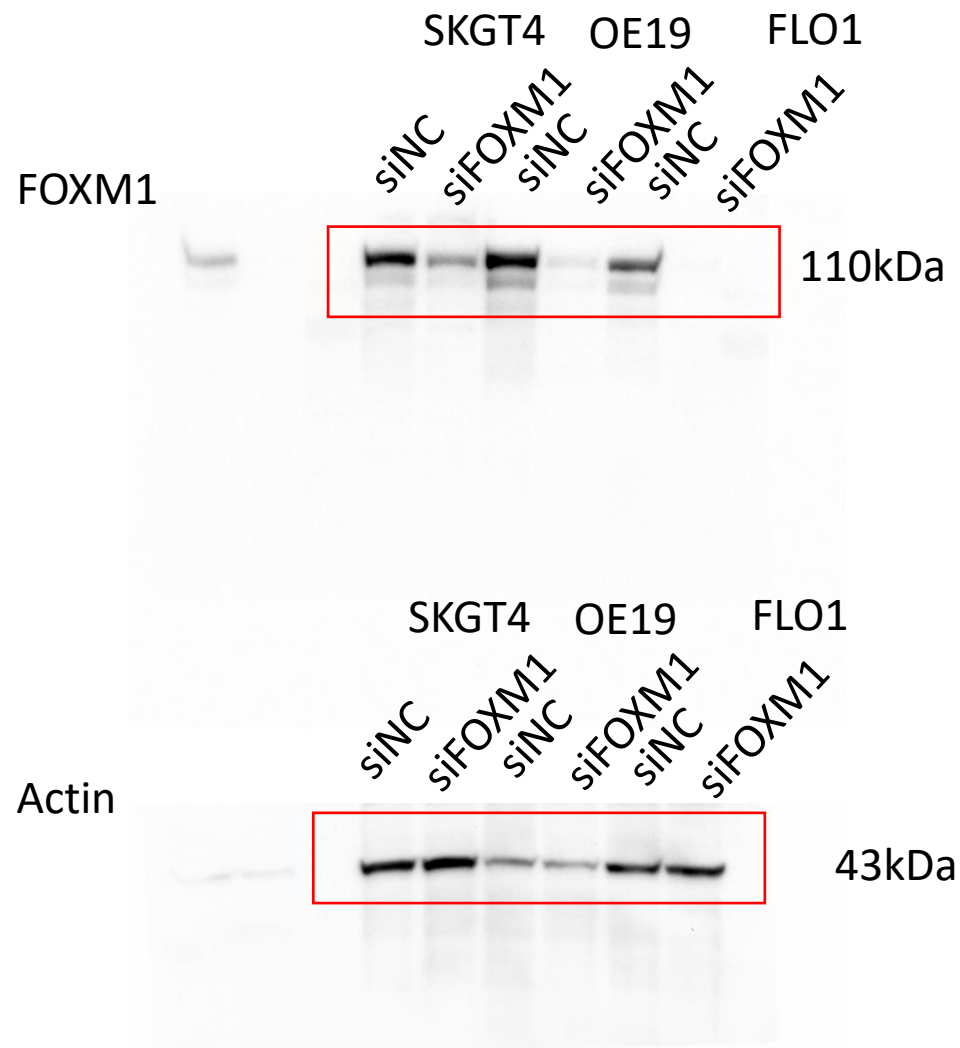

1uM Lapatinib

FOXM1

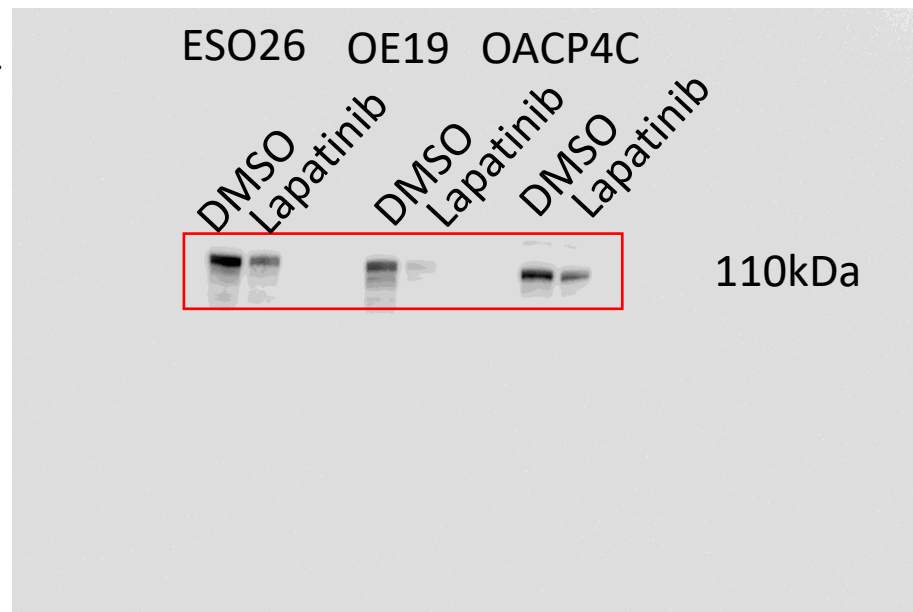

Actin

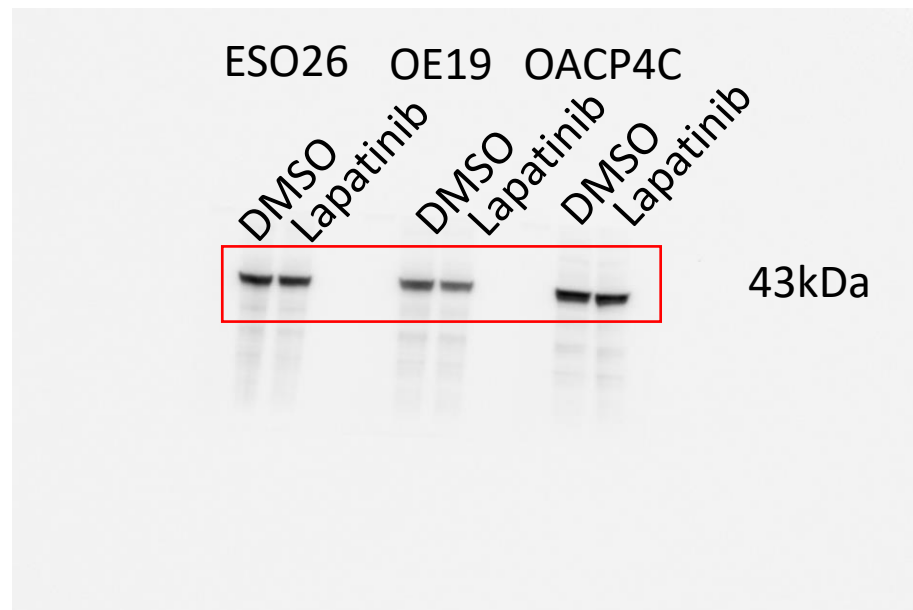

Supplement: Supplementary file 9 — whole wester blot images [file 41419_2024_6488_MOESM9_ESM.pdf]
