# Supplementary material for: Epigenomic analyses identify FOXM1 as a key regulator of anti-tumor immune response in esophageal adenocarcinoma
Source: Cell Death Dis. 2024 Feb 19;15(2):152. doi: 10.1038/s41419-024-06488-x (PMC10876663; doi:10.1038/s41419-024-06488-x)
Supplement: Supplementary file 7 — Table S2 [file 41419_2024_6488_MOESM7_ESM.docx]

| sgRNA |  |
| --- | --- |
| sgScramble | AAAUGUGAGAUCAGAGUAAU |
| sg3FOXM1 | TCCCGATTAGACTCCTGTTGGGCA |
|  |  |
| Mouse shRNA |  |
| shScramble | TAAGGTTAAGTCGCCCTCGGTGTGCTGTCCCGAGGGCGACTTAACCTTA |
| shFOXM1-1 | GAGCATCATCACAGCGCTAGTGTGCTGTCCTAGCGCTGTGATGATGCTC |
| shFOXM1-2 | TGTGAAAGCCTATTGGATTGTGTGCTGTCC AATCCAATAGGCTTTCACA |
