# Supplementary material for: Epigenomic analyses identify FOXM1 as a key regulator of anti-tumor immune response in esophageal adenocarcinoma
Source: Cell Death Dis. 2024 Feb 19;15(2):152. doi: 10.1038/s41419-024-06488-x (PMC10876663; doi:10.1038/s41419-024-06488-x)
Supplement: Supplementary file 8 — Table S3 [file 41419_2024_6488_MOESM8_ESM.docx]

ESO26 Notch Signaling

| SYMBOL | RANK IN GENE LIST | RANK METRIC SCORE | RUNNING ES | CORE ENRICHMENT |
| --- | --- | --- | --- | --- |
| MAML2 | 4048 | 0.231970042 | -0.20854941 | No |
| JAG1 | 5133 | 0.154738858 | -0.25233227 | No |
| NOTCH2 | 6057 | 0.105953969 | -0.2929731 | No |
| DTX2 | 6728 | 0.074679904 | -0.32276466 | No |
| DTX4 | 7996 | 0.022765677 | -0.39453104 | No |
| APH1A | 8442 | 0.005717341 | -0.42003384 | No |
| PPARD | 8526 | 0.003050279 | -0.42453215 | No |
| FBXW11 | 8756 | -0.005284383 | -0.437351 | No |
| KAT2A | 9058 | -0.01692684 | -0.45290017 | No |
| RBX1 | 9174 | -0.020822346 | -0.4569711 | No |
| SKP1 | 9328 | -0.026193665 | -0.46258378 | No |
| FZD7 | 9380 | -0.027906124 | -0.46195713 | No |
| PSENEN | 10250 | -0.059950214 | -0.5054049 | No |
| HES1 | 10615 | -0.073380738 | -0.5173168 | No |
| NOTCH3 | 10905 | -0.084188804 | -0.5233973 | No |
| CUL1 | 10938 | -0.085489422 | -0.51414967 | No |
| FZD1 | 11818 | -0.124174334 | -0.54982203 | No |
| ARRB1 | 11869 | -0.12666063 | -0.53627354 | No |
| PRKCA | 12770 | -0.173120081 | -0.5668093 | No |
| FZD5 | 14099 | -0.274476051 | -0.6093882 | No |
| DLL1 | 14985 | -0.393888414 | -0.6102839 | Yes |
| NOTCH1 | 15191 | -0.43331489 | -0.5659358 | Yes |
| TCF7L2 | 15334 | -0.464343488 | -0.51383024 | Yes |
| PSEN2 | 15469 | -0.501625359 | -0.45639685 | Yes |
| LFNG | 15653 | -0.55782932 | -0.394533 | Yes |
| WNT5A | 15803 | -0.614977539 | -0.32322013 | Yes |
| SAP30 | 15816 | -0.618784726 | -0.24333066 | Yes |
| CCND1 | 16304 | -0.90926373 | -0.15362303 | Yes |
| ST3GAL6 | 16670 | -1.486013532 | 0.018402701 | Yes |

ESO26 G2M Checkpoint

| NAME | SYMBOL | RANK IN GENE LIST | RANK METRIC SCORE | RUNNING ES | CORE ENRICHMENT |
| --- | --- | --- | --- | --- | --- |
| row_0 | NOLC1 | 818 | 1.167953372 | -0.01813832 | No |
| row_1 | HOXC10 | 2012 | 0.541524291 | -0.07499618 | No |
| row_2 | EFNA5 | 2435 | 0.436712652 | -0.08869304 | No |
| row_3 | LIG3 | 2510 | 0.422215819 | -0.08205003 | No |
| row_4 | TLE3 | 2607 | 0.404499978 | -0.07718047 | No |
| row_5 | SMARCC1 | 2817 | 0.366069108 | -0.080044374 | No |
| row_6 | RBL1 | 3047 | 0.333683342 | -0.08494653 | No |
| row_7 | SLC7A1 | 3732 | 0.262140065 | -0.11881067 | No |
| row_8 | MARCKS | 4054 | 0.231537238 | -0.13186334 | No |
| row_9 | WRN | 4778 | 0.178116381 | -0.17024824 | No |
| row_10 | KPNB1 | 4973 | 0.165267631 | -0.17747378 | No |
| row_11 | ATF5 | 5066 | 0.15912579 | -0.17878717 | No |
| row_12 | CCNT1 | 5394 | 0.139771625 | -0.19459847 | No |
| row_13 | CDC25A | 5454 | 0.136656106 | -0.19453509 | No |
| row_14 | FOXN3 | 5908 | 0.112751357 | -0.21855526 | No |
| row_15 | TOP1 | 5914 | 0.112612523 | -0.21590602 | No |
| row_16 | NOTCH2 | 6057 | 0.105953969 | -0.22158775 | No |
| row_17 | SMC1A | 6137 | 0.101658322 | -0.223631 | No |
| row_18 | MEIS2 | 6204 | 0.0981692 | -0.22499155 | No |
| row_19 | RAD23B | 6478 | 0.085412756 | -0.23901027 | No |
| row_20 | ABL1 | 6503 | 0.084638461 | -0.23822431 | No |
| row_21 | RPS6KA5 | 6621 | 0.078877039 | -0.24312615 | No |
| row_22 | CUL5 | 6628 | 0.078721695 | -0.24142334 | No |
| row_23 | CDC27 | 6664 | 0.077503316 | -0.24147902 | No |
| row_24 | MNAT1 | 6863 | 0.068882734 | -0.25146496 | No |
| row_25 | MYBL2 | 6864 | 0.068827324 | -0.24966386 | No |
| row_26 | SMAD3 | 6873 | 0.068407178 | -0.24835004 | No |
| row_27 | PRIM2 | 6885 | 0.068060279 | -0.24722391 | No |
| row_28 | ARID4A | 6992 | 0.063062876 | -0.25188467 | No |
| row_29 | BRCA2 | 7011 | 0.062120613 | -0.25133073 | No |
| row_30 | GSPT1 | 7108 | 0.057870638 | -0.255532 | No |
| row_31 | SQLE | 7117 | 0.057499863 | -0.2545036 | No |
| row_32 | KIF15 | 7177 | 0.055793285 | -0.2565563 | No |
| row_33 | CDK4 | 7240 | 0.05318914 | -0.25885576 | No |
| row_34 | SLC38A1 | 7314 | 0.050223902 | -0.26188776 | No |
| row_35 | BIRC5 | 7341 | 0.049318079 | -0.26214516 | No |
| row_36 | DKC1 | 7561 | 0.040093474 | -0.27413478 | No |
| row_37 | CTCF | 7832 | 0.029085156 | -0.28944892 | No |
| row_38 | HSPA8 | 7850 | 0.028235605 | -0.28972217 | No |
| row_39 | CHAF1A | 8005 | 0.022395959 | -0.29830495 | No |
| row_40 | SLC12A2 | 8015 | 0.021926934 | -0.298267 | No |
| row_41 | TACC3 | 8155 | 0.016781712 | -0.30610362 | No |
| row_42 | CENPE | 8176 | 0.016114909 | -0.30687267 | No |
| row_43 | YTHDC1 | 8193 | 0.015569 | -0.30741787 | No |
| row_44 | DDX39A | 8384 | 0.008283676 | -0.3185133 | No |
| row_45 | CUL3 | 8404 | 0.007453194 | -0.31944948 | No |
| row_46 | HNRNPU | 8422 | 0.006482786 | -0.320292 | No |
| row_47 | CHMP1A | 8575 | 0.001049602 | -0.3293143 | No |
| row_48 | NDC80 | 8762 | -0.005450462 | -0.34024572 | No |
| row_49 | TFDP1 | 8773 | -0.005805179 | -0.3406892 | No |
| row_50 | SFPQ | 8849 | -0.008808002 | -0.34492406 | No |
| row_51 | CDC6 | 9043 | -0.016376229 | -0.35598636 | No |
| row_52 | HNRNPD | 9098 | -0.018089497 | -0.35872802 | No |
| row_53 | NUMA1 | 9265 | -0.02412777 | -0.36797994 | No |
| row_54 | KIF5B | 9327 | -0.026141588 | -0.37092766 | No |
| row_55 | NUP98 | 9336 | -0.02658711 | -0.37070823 | No |
| row_56 | TRA2B | 9339 | -0.026699742 | -0.3701286 | No |
| row_57 | INCENP | 9428 | -0.029347876 | -0.37459993 | No |
| row_58 | G3BP1 | 9450 | -0.030268811 | -0.37505814 | No |
| row_59 | PRPF4B | 9487 | -0.031909686 | -0.3763665 | No |
| row_60 | HMGN2 | 9539 | -0.033778381 | -0.378519 | No |
| row_61 | ESPL1 | 9617 | -0.036151309 | -0.3821574 | No |
| row_62 | MYC | 9668 | -0.037797388 | -0.38414517 | No |
| row_63 | CDC45 | 9684 | -0.038415309 | -0.38403296 | No |
| row_64 | HUS1 | 9735 | -0.040152516 | -0.38595912 | No |
| row_65 | PAFAH1B1 | 9746 | -0.040373061 | -0.385498 | No |
| row_66 | PML | 9760 | -0.041073442 | -0.38519716 | No |
| row_67 | ODC1 | 9768 | -0.04146241 | -0.3845289 | No |
| row_68 | KIF22 | 9774 | -0.041818984 | -0.38373226 | No |
| row_69 | POLA2 | 9849 | -0.044032108 | -0.3869858 | No |
| row_70 | NASP | 9881 | -0.045272164 | -0.38764676 | No |
| row_71 | EWSR1 | 9914 | -0.046496559 | -0.38833523 | No |
| row_72 | CBX1 | 9989 | -0.04967716 | -0.39144105 | No |
| row_73 | ILF3 | 10023 | -0.051008444 | -0.39207098 | No |
| row_74 | HMMR | 10028 | -0.051316615 | -0.39096627 | No |
| row_75 | CUL4A | 10096 | -0.053980913 | -0.3935427 | No |
| row_76 | EGF | 10259 | -0.060321271 | -0.40160933 | No |
| row_77 | PRC1 | 10404 | -0.066267431 | -0.40844867 | No |
| row_78 | PDS5B | 10521 | -0.070244364 | -0.41351688 | No |
| row_79 | NCL | 10525 | -0.070331067 | -0.411855 | No |
| row_80 | RASAL2 | 10536 | -0.070815392 | -0.41059726 | No |
| row_81 | BCL3 | 10554 | -0.071314454 | -0.4097432 | No |
| row_82 | AMD1 | 10591 | -0.072778061 | -0.40998206 | No |
| row_83 | ORC6 | 10594 | -0.072811812 | -0.40819576 | No |
| row_84 | STAG1 | 10612 | -0.073283061 | -0.4072902 | No |
| row_85 | NUSAP1 | 10683 | -0.076347254 | -0.40945995 | No |
| row_86 | MCM3 | 10720 | -0.077929534 | -0.409564 | No |
| row_87 | UPF1 | 10733 | -0.078216277 | -0.40823165 | No |
| row_88 | BUB3 | 10836 | -0.081974089 | -0.41215935 | No |
| row_89 | SRSF2 | 10857 | -0.082752936 | -0.41118458 | No |
| row_90 | SRSF1 | 10873 | -0.083090149 | -0.4099033 | No |
| row_91 | RACGAP1 | 10929 | -0.085097201 | -0.41095102 | No |
| row_92 | CUL1 | 10938 | -0.085489422 | -0.40919018 | No |
| row_93 | AURKB | 10941 | -0.085543647 | -0.4070707 | No |
| row_94 | E2F1 | 11027 | -0.089880317 | -0.40977937 | No |
| row_95 | TOP2A | 11032 | -0.089936562 | -0.407664 | No |
| row_96 | RAD54L | 11075 | -0.092200428 | -0.40775183 | No |
| row_97 | EZH2 | 11082 | -0.092746414 | -0.40568203 | No |
| row_98 | BUB1 | 11167 | -0.096104115 | -0.4081683 | No |
| row_99 | CKS1B | 11200 | -0.097467385 | -0.40752292 | No |
| row_100 | PRMT5 | 11228 | -0.098761611 | -0.406546 | No |
| row_101 | TRAIP | 11244 | -0.099610008 | -0.4048324 | No |
| row_102 | E2F4 | 11253 | -0.099776186 | -0.4026977 | No |
| row_103 | HIF1A | 11307 | -0.101846911 | -0.40318802 | No |
| row_104 | ORC5 | 11394 | -0.105463937 | -0.40554842 | No |
| row_105 | MCM5 | 11501 | -0.109774999 | -0.4089868 | No |
| row_106 | PURA | 11564 | -0.112756714 | -0.40972745 | No |
| row_107 | TNPO2 | 11691 | -0.11812266 | -0.41413814 | No |
| row_108 | PLK4 | 11712 | -0.119062096 | -0.4122132 | No |
| row_109 | UBE2S | 11755 | -0.121454142 | -0.4115355 | No |
| row_110 | BARD1 | 11813 | -0.123926841 | -0.41168618 | No |
| row_111 | XPO1 | 11897 | -0.127866194 | -0.41328174 | No |
| row_112 | DMD | 12032 | -0.135170743 | -0.4177226 | Yes |
| row_113 | SMC2 | 12042 | -0.135653406 | -0.41470858 | Yes |
| row_114 | SMC4 | 12059 | -0.136375472 | -0.41209242 | Yes |
| row_115 | STMN1 | 12216 | -0.143757969 | -0.4176184 | Yes |
| row_116 | SNRPD1 | 12221 | -0.144121617 | -0.4140851 | Yes |
| row_117 | CHEK1 | 12310 | -0.148544014 | -0.41543725 | Yes |
| row_118 | SRSF10 | 12362 | -0.150821969 | -0.41452688 | Yes |
| row_119 | ODF2 | 12385 | -0.152047828 | -0.4118578 | Yes |
| row_120 | UCK2 | 12391 | -0.152210563 | -0.40817237 | Yes |
| row_121 | NUP50 | 12411 | -0.153415516 | -0.4052889 | Yes |
| row_122 | KIF20B | 12422 | -0.153671846 | -0.40186292 | Yes |
| row_123 | SS18 | 12474 | -0.156646624 | -0.4008001 | Yes |
| row_124 | HMGA1 | 12499 | -0.158196896 | -0.39808923 | Yes |
| row_125 | TTK | 12545 | -0.160560757 | -0.39656678 | Yes |
| row_126 | SYNCRIP | 12575 | -0.162334874 | -0.3940453 | Yes |
| row_127 | MTF2 | 12615 | -0.163543582 | -0.39208758 | Yes |
| row_128 | MCM6 | 12617 | -0.163568884 | -0.38786674 | Yes |
| row_129 | MKI67 | 12633 | -0.164532632 | -0.38445422 | Yes |
| row_130 | KIF4A | 12638 | -0.164825752 | -0.38037908 | Yes |
| row_131 | EXO1 | 12850 | -0.177050084 | -0.38830844 | Yes |
| row_132 | KIF11 | 12870 | -0.178428575 | -0.38477042 | Yes |
| row_133 | MCM2 | 12985 | -0.184968457 | -0.38671738 | Yes |
| row_134 | DR1 | 12993 | -0.185251847 | -0.38228637 | Yes |
| row_135 | POLE | 13148 | -0.193979204 | -0.38637903 | Yes |
| row_136 | TMPO | 13468 | -0.216994077 | -0.39969322 | Yes |
| row_137 | CDC20 | 13544 | -0.223620892 | -0.39830673 | Yes |
| row_138 | RPA2 | 13599 | -0.228930593 | -0.39553097 | Yes |
| row_139 | AURKA | 13621 | -0.230222151 | -0.39075667 | Yes |
| row_140 | TPX2 | 13663 | -0.233330324 | -0.3870918 | Yes |
| row_141 | DTYMK | 13667 | -0.233760104 | -0.38115323 | Yes |
| row_142 | RBM14 | 13726 | -0.238516793 | -0.37836477 | Yes |
| row_143 | MEIS1 | 13790 | -0.244186297 | -0.37572563 | Yes |
| row_144 | CDC7 | 13815 | -0.247035578 | -0.37068996 | Yes |
| row_145 | FBXO5 | 13872 | -0.252553135 | -0.3674151 | Yes |
| row_146 | CKS2 | 13874 | -0.252683699 | -0.36086226 | Yes |
| row_147 | MAPK14 | 13918 | -0.25629738 | -0.35671544 | Yes |
| row_148 | HIRA | 14001 | -0.265128404 | -0.3546595 | Yes |
| row_149 | CDKN1B | 14016 | -0.266339481 | -0.3485233 | Yes |
| row_150 | STIL | 14076 | -0.272829503 | -0.34489647 | Yes |
| row_151 | MAD2L1 | 14176 | -0.281245261 | -0.3434309 | Yes |
| row_152 | E2F2 | 14213 | -0.28469032 | -0.3381243 | Yes |
| row_153 | UBE2C | 14236 | -0.287833542 | -0.33190194 | Yes |
| row_154 | RAD21 | 14320 | -0.298637807 | -0.32902864 | Yes |
| row_155 | POLQ | 14359 | -0.302579045 | -0.32337302 | Yes |
| row_156 | KATNA1 | 14402 | -0.308454216 | -0.31780177 | Yes |
| row_157 | PBK | 14466 | -0.316604942 | -0.31326756 | Yes |
| row_158 | PLK1 | 14507 | -0.321772277 | -0.3072287 | Yes |
| row_159 | HMGB3 | 14710 | -0.348112255 | -0.31014577 | Yes |
| row_160 | LMNB1 | 14748 | -0.353913784 | -0.30308723 | Yes |
| row_161 | GINS2 | 14785 | -0.36108765 | -0.29578143 | Yes |
| row_162 | E2F3 | 14786 | -0.36110267 | -0.28633186 | Yes |
| row_163 | SUV39H1 | 14849 | -0.371700078 | -0.28029633 | Yes |
| row_164 | CENPF | 14871 | -0.373942107 | -0.27176106 | Yes |
| row_165 | KIF23 | 15051 | -0.405675948 | -0.2718024 | Yes |
| row_166 | FANCC | 15074 | -0.410611987 | -0.26236707 | Yes |
| row_167 | KIF2C | 15093 | -0.413861305 | -0.25260857 | Yes |
| row_168 | ATRX | 15107 | -0.415624797 | -0.2425062 | Yes |
| row_169 | DBF4 | 15172 | -0.429754466 | -0.23507056 | Yes |
| row_170 | LBR | 15235 | -0.442696035 | -0.22717716 | Yes |
| row_171 | CDKN2C | 15407 | -0.482022941 | -0.22474426 | Yes |
| row_172 | MT2A | 15487 | -0.505933106 | -0.21620819 | Yes |
| row_173 | TGFB1 | 15499 | -0.510027289 | -0.20351638 | Yes |
| row_174 | CDC25B | 15618 | -0.5488379 | -0.19617952 | Yes |
| row_175 | CCNB2 | 15650 | -0.557093084 | -0.18344682 | Yes |
| row_176 | CDK1 | 15741 | -0.58914119 | -0.17338823 | Yes |
| row_177 | SAP30 | 15816 | -0.618784726 | -0.16160128 | Yes |
| row_178 | SLC7A5 | 15882 | -0.649424613 | -0.14847669 | Yes |
| row_179 | CCNA2 | 16057 | -0.736903191 | -0.13955255 | Yes |
| row_180 | PTTG1 | 16131 | -0.780340493 | -0.12347837 | Yes |
| row_181 | CENPA | 16219 | -0.838700712 | -0.10671051 | Yes |
| row_182 | CCNF | 16303 | -0.908327162 | -0.087882474 | Yes |
| row_183 | CCND1 | 16304 | -0.90926373 | -0.06408828 | Yes |
| row_184 | NEK2 | 16549 | -1.213480115 | -0.046860423 | Yes |
| row_185 | TROAP | 16575 | -1.260965466 | -0.015351118 | Yes |
| row_186 | CDKN3 | 16677 | -1.510334969 | 0.018158961 | Yes |

ESO26 E2F Targets

| NAME | SYMBOL | RANK IN GENE LIST | RANK METRIC SCORE | RUNNING ES | CORE ENRICHMENT |
| --- | --- | --- | --- | --- | --- |
| row_0 | NOLC1 | 818 | 1.167953372 | -0.01430261 | No |
| row_1 | ZW10 | 1045 | 0.975589871 | 9.85E-04 | No |
| row_2 | PRDX4 | 2134 | 0.506501913 | -0.04888968 | No |
| row_3 | PHF5A | 2384 | 0.447051018 | -0.050546385 | No |
| row_4 | TIPIN | 3651 | 0.269659609 | -0.11800217 | No |
| row_5 | CSE1L | 3884 | 0.248554289 | -0.1244956 | No |
| row_6 | PPP1R8 | 3922 | 0.244440854 | -0.11949619 | No |
| row_7 | CCP110 | 4447 | 0.200893566 | -0.14478537 | No |
| row_8 | UNG | 5037 | 0.161046878 | -0.1751201 | No |
| row_9 | LUC7L3 | 5080 | 0.158427328 | -0.17295311 | No |
| row_10 | TP53 | 5117 | 0.15578194 | -0.17050672 | No |
| row_11 | PRKDC | 5287 | 0.145612016 | -0.17628138 | No |
| row_12 | CDC25A | 5454 | 0.136656106 | -0.18214127 | No |
| row_13 | TFRC | 5638 | 0.126929402 | -0.18930031 | No |
| row_14 | GINS4 | 5765 | 0.120719939 | -0.19324744 | No |
| row_15 | SMC6 | 5777 | 0.120126501 | -0.19036275 | No |
| row_16 | PNN | 6011 | 0.107822642 | -0.20106277 | No |
| row_17 | SMC1A | 6137 | 0.101658322 | -0.20551205 | No |
| row_18 | CTPS1 | 6166 | 0.099815704 | -0.20423837 | No |
| row_19 | RAD50 | 6315 | 0.093434684 | -0.21029983 | No |
| row_20 | NBN | 6356 | 0.091272295 | -0.20999262 | No |
| row_21 | PRPS1 | 6410 | 0.088678896 | -0.2105361 | No |
| row_22 | CBX5 | 6491 | 0.084914602 | -0.21279861 | No |
| row_23 | DIAPH3 | 6630 | 0.078593612 | -0.21870182 | No |
| row_24 | MYBL2 | 6864 | 0.068827324 | -0.23055094 | No |
| row_25 | PRIM2 | 6885 | 0.068060279 | -0.22973655 | No |
| row_26 | BRCA2 | 7011 | 0.062120613 | -0.2353509 | No |
| row_27 | RPA1 | 7021 | 0.061721157 | -0.23406817 | No |
| row_28 | UBR7 | 7078 | 0.058992606 | -0.23566511 | No |
| row_29 | GSPT1 | 7108 | 0.057870638 | -0.23568702 | No |
| row_30 | CDK4 | 7240 | 0.05318914 | -0.24192192 | No |
| row_31 | BIRC5 | 7341 | 0.049318079 | -0.24642456 | No |
| row_32 | NAP1L1 | 7584 | 0.03944812 | -0.25967544 | No |
| row_33 | TCF19 | 7791 | 0.030790217 | -0.27103734 | No |
| row_34 | CDCA3 | 7824 | 0.029357776 | -0.27207813 | No |
| row_35 | CTCF | 7832 | 0.029085156 | -0.27163798 | No |
| row_36 | MELK | 7903 | 0.026846623 | -0.275016 | No |
| row_37 | DUT | 7972 | 0.023759056 | -0.2783659 | No |
| row_38 | PMS2 | 7986 | 0.023120739 | -0.2784589 | No |
| row_39 | TRIP13 | 8002 | 0.022487216 | -0.27868962 | No |
| row_40 | BUB1B | 8121 | 0.017608138 | -0.28519875 | No |
| row_41 | TACC3 | 8155 | 0.016781712 | -0.28666967 | No |
| row_42 | CENPE | 8176 | 0.016114909 | -0.287386 | No |
| row_43 | CHEK2 | 8254 | 0.013214234 | -0.29158267 | No |
| row_44 | DDX39A | 8384 | 0.008283676 | -0.29902172 | No |
| row_45 | NUP153 | 8433 | 0.006025139 | -0.301703 | No |
| row_46 | EIF2S1 | 8619 | -4.56E-04 | -0.31270805 | No |
| row_47 | CDKN2A | 8665 | -0.002403379 | -0.3153174 | No |
| row_48 | RFC2 | 8838 | -0.008473477 | -0.3253119 | No |
| row_49 | RAD51C | 8909 | -0.010871191 | -0.3291607 | No |
| row_50 | NUDT21 | 8953 | -0.012594564 | -0.3313506 | No |
| row_51 | EXOSC8 | 9097 | -0.018089453 | -0.33933452 | No |
| row_52 | HNRNPD | 9098 | -0.018089497 | -0.33880147 | No |
| row_53 | DEPDC1 | 9176 | -0.020893503 | -0.34277186 | No |
| row_54 | POLD2 | 9207 | -0.022035684 | -0.3439093 | No |
| row_55 | TIMELESS | 9301 | -0.025138948 | -0.3487075 | No |
| row_56 | TRA2B | 9339 | -0.026699742 | -0.35012442 | No |
| row_57 | NUP205 | 9366 | -0.027531067 | -0.3508617 | No |
| row_58 | ESPL1 | 9617 | -0.036151309 | -0.36468622 | No |
| row_59 | PAICS | 9658 | -0.037513562 | -0.36596313 | No |
| row_60 | MYC | 9668 | -0.037797388 | -0.36538538 | No |
| row_61 | NCAPD2 | 9718 | -0.039612673 | -0.36713648 | No |
| row_62 | HUS1 | 9735 | -0.040152516 | -0.36690623 | No |
| row_63 | KIF22 | 9774 | -0.041818984 | -0.36793718 | No |
| row_64 | POLA2 | 9849 | -0.044032108 | -0.37104705 | No |
| row_65 | NASP | 9881 | -0.045272164 | -0.37155932 | No |
| row_66 | E2F8 | 9882 | -0.045337733 | -0.3702233 | No |
| row_67 | PA2G4 | 9892 | -0.045644954 | -0.3694143 | No |
| row_68 | MLH1 | 9916 | -0.046511792 | -0.36941355 | No |
| row_69 | ILF3 | 10023 | -0.051008444 | -0.37422374 | No |
| row_70 | HMMR | 10028 | -0.051316615 | -0.3729498 | No |
| row_71 | RANBP1 | 10084 | -0.053331446 | -0.374654 | No |
| row_72 | AK2 | 10093 | -0.053720627 | -0.37354746 | No |
| row_73 | POP7 | 10160 | -0.056492232 | -0.37581366 | No |
| row_74 | TBRG4 | 10260 | -0.060354326 | -0.37993154 | No |
| row_75 | GINS3 | 10463 | -0.068041213 | -0.3899575 | Yes |
| row_76 | NME1 | 10479 | -0.068740271 | -0.38882527 | Yes |
| row_77 | PDS5B | 10521 | -0.070244364 | -0.38919726 | Yes |
| row_78 | SHMT1 | 10540 | -0.071032785 | -0.38817614 | Yes |
| row_79 | BRCA1 | 10555 | -0.071317635 | -0.3869084 | Yes |
| row_80 | GINS1 | 10580 | -0.072211802 | -0.38620993 | Yes |
| row_81 | ORC6 | 10594 | -0.072811812 | -0.3848386 | Yes |
| row_82 | RFC1 | 10602 | -0.072967306 | -0.38310534 | Yes |
| row_83 | STAG1 | 10612 | -0.073283061 | -0.3814819 | Yes |
| row_84 | PSMC3IP | 10642 | -0.074474886 | -0.38101453 | Yes |
| row_85 | DNMT1 | 10678 | -0.076059945 | -0.38085777 | Yes |
| row_86 | MCM3 | 10720 | -0.077929534 | -0.3810033 | Yes |
| row_87 | RNASEH2A | 10738 | -0.078380324 | -0.3797061 | Yes |
| row_88 | IPO7 | 10767 | -0.079471476 | -0.37903196 | Yes |
| row_89 | WEE1 | 10772 | -0.079715572 | -0.37692115 | Yes |
| row_90 | SRSF2 | 10857 | -0.082752936 | -0.3794856 | Yes |
| row_91 | XRCC6 | 10859 | -0.082817078 | -0.37710473 | Yes |
| row_92 | SRSF1 | 10873 | -0.083090149 | -0.37543052 | Yes |
| row_93 | SPC24 | 10921 | -0.084849171 | -0.3757295 | Yes |
| row_94 | RACGAP1 | 10929 | -0.085097201 | -0.3736388 | Yes |
| row_95 | AURKB | 10941 | -0.085543647 | -0.37177318 | Yes |
| row_96 | TOP2A | 11032 | -0.089936562 | -0.3744833 | Yes |
| row_97 | SPC25 | 11050 | -0.090701275 | -0.37282303 | Yes |
| row_98 | EZH2 | 11082 | -0.092746414 | -0.37193635 | Yes |
| row_99 | SMC3 | 11115 | -0.093878753 | -0.37107587 | Yes |
| row_100 | ING3 | 11177 | -0.09659823 | -0.37186247 | Yes |
| row_101 | NUP107 | 11179 | -0.096653268 | -0.36907387 | Yes |
| row_102 | CKS1B | 11200 | -0.097467385 | -0.36739293 | Yes |
| row_103 | HELLS | 11223 | -0.09842246 | -0.36580294 | Yes |
| row_104 | LIG1 | 11238 | -0.099212505 | -0.3637132 | Yes |
| row_105 | TUBG1 | 11305 | -0.10169445 | -0.36464742 | Yes |
| row_106 | POLD1 | 11423 | -0.106433719 | -0.3684795 | Yes |
| row_107 | MTHFD2 | 11475 | -0.108706899 | -0.36831367 | Yes |
| row_108 | DEK | 11497 | -0.109653063 | -0.36633322 | Yes |
| row_109 | MCM5 | 11501 | -0.109774999 | -0.36327708 | Yes |
| row_110 | RBBP7 | 11510 | -0.110143885 | -0.36050788 | Yes |
| row_111 | SSRP1 | 11512 | -0.110237435 | -0.35731897 | Yes |
| row_112 | PLK4 | 11712 | -0.119062096 | -0.36566278 | Yes |
| row_113 | UBE2S | 11755 | -0.121454142 | -0.3645853 | Yes |
| row_114 | BARD1 | 11813 | -0.123926841 | -0.36432835 | Yes |
| row_115 | DCK | 11823 | -0.124376312 | -0.3611993 | Yes |
| row_116 | XPO1 | 11897 | -0.127866194 | -0.3617792 | Yes |
| row_117 | SMC4 | 12059 | -0.136375472 | -0.3673496 | Yes |
| row_118 | STMN1 | 12216 | -0.143757969 | -0.3724046 | Yes |
| row_119 | CHEK1 | 12310 | -0.148544014 | -0.3735664 | Yes |
| row_120 | RAN | 12421 | -0.153669387 | -0.3755896 | Yes |
| row_121 | HMGA1 | 12499 | -0.158196896 | -0.375514 | Yes |
| row_122 | SNRPB | 12564 | -0.161652118 | -0.37456226 | Yes |
| row_123 | SYNCRIP | 12575 | -0.162334874 | -0.37037423 | Yes |
| row_124 | TK1 | 12589 | -0.162742913 | -0.36635286 | Yes |
| row_125 | MCM6 | 12617 | -0.163568884 | -0.36314094 | Yes |
| row_126 | MKI67 | 12633 | -0.164532632 | -0.35918596 | Yes |
| row_127 | KIF4A | 12638 | -0.164825752 | -0.35456717 | Yes |
| row_128 | RAD51AP1 | 12643 | -0.165133819 | -0.3499393 | Yes |
| row_129 | POLE4 | 12714 | -0.169189826 | -0.3491228 | Yes |
| row_130 | DCLRE1B | 12796 | -0.174481928 | -0.34880552 | Yes |
| row_131 | USP1 | 12815 | -0.175469622 | -0.34470692 | Yes |
| row_132 | ANP32E | 12856 | -0.17761004 | -0.34185553 | Yes |
| row_133 | EED | 12918 | -0.181143403 | -0.34015077 | Yes |
| row_134 | MCM2 | 12985 | -0.184968457 | -0.3386311 | Yes |
| row_135 | DLGAP5 | 13006 | -0.186003968 | -0.33434117 | Yes |
| row_136 | POLE | 13148 | -0.193979204 | -0.3370229 | Yes |
| row_137 | POLD3 | 13174 | -0.196234584 | -0.3327293 | Yes |
| row_138 | ATAD2 | 13188 | -0.197381005 | -0.32768723 | Yes |
| row_139 | PAN2 | 13197 | -0.197859123 | -0.32233325 | Yes |
| row_140 | LYAR | 13205 | -0.198130742 | -0.31691173 | Yes |
| row_141 | CENPM | 13220 | -0.198757827 | -0.3118886 | Yes |
| row_142 | DONSON | 13294 | -0.204303414 | -0.3102161 | Yes |
| row_143 | SPAG5 | 13318 | -0.206022799 | -0.30551493 | Yes |
| row_144 | ASF1B | 13349 | -0.207901284 | -0.30117536 | Yes |
| row_145 | PPM1D | 13354 | -0.208179459 | -0.29527903 | Yes |
| row_146 | NOP56 | 13386 | -0.210086077 | -0.29093462 | Yes |
| row_147 | UBE2T | 13433 | -0.213480368 | -0.2873836 | Yes |
| row_148 | TMPO | 13468 | -0.216994077 | -0.28301427 | Yes |
| row_149 | MCM7 | 13515 | -0.220523909 | -0.2792557 | Yes |
| row_150 | RFC3 | 13535 | -0.22284703 | -0.27382052 | Yes |
| row_151 | CDC20 | 13544 | -0.223620892 | -0.2677074 | Yes |
| row_152 | MCM4 | 13577 | -0.22661376 | -0.26293552 | Yes |
| row_153 | RPA2 | 13599 | -0.228930593 | -0.2574402 | Yes |
| row_154 | NAA38 | 13615 | -0.229767308 | -0.2515629 | Yes |
| row_155 | AURKA | 13621 | -0.230222151 | -0.24507658 | Yes |
| row_156 | ASF1A | 13664 | -0.23337175 | -0.24070115 | Yes |
| row_157 | MSH2 | 13690 | -0.235617444 | -0.23524703 | Yes |
| row_158 | DSCC1 | 13722 | -0.237802103 | -0.2300859 | Yes |
| row_159 | RRM2 | 13767 | -0.242045209 | -0.225574 | Yes |
| row_160 | SLBP | 13805 | -0.246115834 | -0.22052523 | Yes |
| row_161 | RPA3 | 13868 | -0.252382338 | -0.21678078 | Yes |
| row_162 | CKS2 | 13874 | -0.252683699 | -0.20963258 | Yes |
| row_163 | CDKN1B | 14016 | -0.266339481 | -0.21018203 | Yes |
| row_164 | DCTPP1 | 14092 | -0.273785681 | -0.20658115 | Yes |
| row_165 | MAD2L1 | 14176 | -0.281245261 | -0.20323692 | Yes |
| row_166 | CIT | 14239 | -0.28812325 | -0.19843927 | Yes |
| row_167 | RAD1 | 14254 | -0.289494962 | -0.19074236 | Yes |
| row_168 | CCNE1 | 14258 | -0.290272862 | -0.18236737 | Yes |
| row_169 | RAD21 | 14320 | -0.298637807 | -0.17720033 | Yes |
| row_170 | PCNA | 14338 | -0.300506741 | -0.1693576 | Yes |
| row_171 | MMS22L | 14381 | -0.305563748 | -0.16285484 | Yes |
| row_172 | HMGB2 | 14394 | -0.307105422 | -0.15451987 | Yes |
| row_173 | PLK1 | 14507 | -0.321772277 | -0.15170863 | Yes |
| row_174 | CDCA8 | 14590 | -0.331643283 | -0.14681973 | Yes |
| row_175 | HMGB3 | 14710 | -0.348112255 | -0.14364922 | Yes |
| row_176 | LMNB1 | 14748 | -0.353913784 | -0.1354239 | Yes |
| row_177 | BRMS1L | 14832 | -0.369551182 | -0.1294775 | Yes |
| row_178 | SUV39H1 | 14849 | -0.371700078 | -0.11947732 | Yes |
| row_179 | ORC2 | 14856 | -0.37222451 | -0.10886609 | Yes |
| row_180 | PSIP1 | 14858 | -0.372370601 | -0.09795275 | Yes |
| row_181 | WDR90 | 14982 | -0.393367022 | -0.09368693 | Yes |
| row_182 | KIF2C | 15093 | -0.413861305 | -0.088042915 | Yes |
| row_183 | LBR | 15235 | -0.442696035 | -0.08339555 | Yes |
| row_184 | CDKN2C | 15407 | -0.482022941 | -0.079376094 | Yes |
| row_185 | MXD3 | 15607 | -0.545603752 | -0.07515071 | Yes |
| row_186 | CDC25B | 15618 | -0.5488379 | -0.05957333 | Yes |
| row_187 | CCNB2 | 15650 | -0.557093084 | -0.04500343 | Yes |
| row_188 | CDK1 | 15741 | -0.58914119 | -0.033003144 | Yes |
| row_189 | KIF18B | 15885 | -0.650936365 | -0.022338537 | Yes |
| row_190 | CDKN1A | 15940 | -0.678551853 | -0.005559394 | Yes |
| row_191 | PTTG1 | 16131 | -0.780340493 | 0.006119164 | Yes |
| row_192 | CDKN3 | 16677 | -1.510334969 | 0.01816542 | Yes |

ESO26 IL2 Stat5 Signaling

| NAME | SYMBOL | RANK IN GENE LIST | RANK METRIC SCORE | RUNNING ES | CORE ENRICHMENT |
| --- | --- | --- | --- | --- | --- |
| row_0 | PLEC | 0 | 23.54065514 | 0.28383163 | Yes |
| row_1 | TNFRSF4 | 101 | 3.573562622 | 0.32096988 | Yes |
| row_2 | SCN9A | 161 | 2.768275976 | 0.3508376 | Yes |
| row_3 | SLC2A3 | 177 | 2.628319502 | 0.3816352 | Yes |
| row_4 | TIAM1 | 209 | 2.423045397 | 0.40900603 | Yes |
| row_5 | AGER | 257 | 2.219220877 | 0.43296757 | Yes |
| row_6 | IL13 | 621 | 1.412258029 | 0.42840227 | Yes |
| row_7 | CSF2 | 667 | 1.365479231 | 0.44218916 | Yes |
| row_8 | SELL | 767 | 1.225902677 | 0.45108095 | Yes |
| row_9 | GADD45B | 907 | 1.086149454 | 0.45590836 | Yes |
| row_10 | PRAF2 | 998 | 1.004213691 | 0.4626626 | Yes |
| row_11 | IL10RA | 1068 | 0.960665703 | 0.47014096 | Yes |
| row_12 | DENND5A | 1420 | 0.755158782 | 0.45836678 | No |
| row_13 | GPR83 | 1593 | 0.677832067 | 0.45630807 | No |
| row_14 | BATF3 | 1734 | 0.626174033 | 0.45553002 | No |
| row_15 | EMP1 | 1830 | 0.597337127 | 0.4570811 | No |
| row_16 | TNFRSF9 | 1889 | 0.583548665 | 0.4606669 | No |
| row_17 | TRAF1 | 1947 | 0.564714193 | 0.46408504 | No |
| row_18 | RHOB | 2148 | 0.501393378 | 0.45823342 | No |
| row_19 | IGF1R | 2601 | 0.405339837 | 0.4362335 | No |
| row_20 | ITGAV | 2972 | 0.341702908 | 0.41834402 | No |
| row_21 | LTB | 3014 | 0.337170929 | 0.41997042 | No |
| row_22 | IGF2R | 3045 | 0.333966047 | 0.42221254 | No |
| row_23 | HUWE1 | 3116 | 0.32724151 | 0.42199418 | No |
| row_24 | BCL2 | 3124 | 0.326483011 | 0.42551422 | No |
| row_25 | AHNAK | 3230 | 0.312736928 | 0.42303902 | No |
| row_26 | ANXA4 | 3238 | 0.311896771 | 0.42638317 | No |
| row_27 | BMP2 | 3287 | 0.303825229 | 0.42719114 | No |
| row_28 | BATF | 3361 | 0.297515571 | 0.42643592 | No |
| row_29 | MAFF | 3424 | 0.292279184 | 0.4262719 | No |
| row_30 | HIPK2 | 3542 | 0.279130965 | 0.42267767 | No |
| row_31 | IRF6 | 3770 | 0.258589059 | 0.41229245 | No |
| row_32 | TNFRSF21 | 3795 | 0.256606996 | 0.41395873 | No |
| row_33 | ABCB1 | 3919 | 0.244521648 | 0.4095903 | No |
| row_34 | TLR7 | 4068 | 0.230626017 | 0.40356722 | No |
| row_35 | ALCAM | 4090 | 0.229279816 | 0.4050825 | No |
| row_36 | ST3GAL4 | 4094 | 0.228879645 | 0.40766367 | No |
| row_37 | CD44 | 4169 | 0.222843841 | 0.40594864 | No |
| row_38 | BCL2L1 | 4242 | 0.21710217 | 0.40428334 | No |
| row_39 | SH3BGRL2 | 4305 | 0.211406216 | 0.4031442 | No |
| row_40 | SERPINB6 | 4472 | 0.199004173 | 0.39566913 | No |
| row_41 | SYNGR2 | 4597 | 0.190493092 | 0.3905898 | No |
| row_42 | IFNGR1 | 4616 | 0.189266667 | 0.3918011 | No |
| row_43 | GABARAPL1 | 4645 | 0.187166676 | 0.3923922 | No |
| row_44 | IKZF2 | 4648 | 0.187077761 | 0.39452884 | No |
| row_45 | TTC39B | 4665 | 0.185844928 | 0.39581782 | No |
| row_46 | PDCD2L | 4808 | 0.176389202 | 0.38949773 | No |
| row_47 | GBP4 | 4945 | 0.167109281 | 0.3834226 | No |
| row_48 | RGS16 | 4981 | 0.164758131 | 0.38332716 | No |
| row_49 | PLIN2 | 5012 | 0.162789613 | 0.38350537 | No |
| row_50 | KLF6 | 5087 | 0.158143327 | 0.38101023 | No |
| row_51 | UMPS | 5146 | 0.153661221 | 0.37941283 | No |
| row_52 | SNX14 | 5283 | 0.1458859 | 0.37308186 | No |
| row_53 | DHRS3 | 5318 | 0.14355889 | 0.37279028 | No |
| row_54 | IL18R1 | 5380 | 0.140262753 | 0.37085286 | No |
| row_55 | SLC1A5 | 5621 | 0.127676472 | 0.35811588 | No |
| row_56 | AHCY | 5787 | 0.11948777 | 0.34974155 | No |
| row_57 | CYFIP1 | 5893 | 0.11382129 | 0.344868 | No |
| row_58 | IKZF4 | 6025 | 0.107470244 | 0.33837128 | No |
| row_59 | RNH1 | 6069 | 0.10549964 | 0.33708543 | No |
| row_60 | CKAP4 | 6074 | 0.105162159 | 0.33811545 | No |
| row_61 | SHE | 6209 | 0.097860053 | 0.33132437 | No |
| row_62 | MXD1 | 6235 | 0.096740119 | 0.33100367 | No |
| row_63 | WLS | 6398 | 0.089590244 | 0.3224473 | No |
| row_64 | DRC1 | 6401 | 0.089459963 | 0.32340696 | No |
| row_65 | NFIL3 | 6444 | 0.087074324 | 0.32195845 | No |
| row_66 | MYO1C | 6543 | 0.082550019 | 0.31712425 | No |
| row_67 | TNFSF10 | 6605 | 0.079443641 | 0.31445354 | No |
| row_68 | COCH | 6608 | 0.079303354 | 0.31529075 | No |
| row_69 | POU2F1 | 6748 | 0.07396318 | 0.3079141 | No |
| row_70 | PRKCH | 6773 | 0.073058404 | 0.30736735 | No |
| row_71 | IRF8 | 6896 | 0.067548446 | 0.30092463 | No |
| row_72 | NCOA3 | 6922 | 0.066355042 | 0.30023757 | No |
| row_73 | NDRG1 | 6954 | 0.065211743 | 0.2991798 | No |
| row_74 | ENPP1 | 7128 | 0.056941848 | 0.28957546 | No |
| row_75 | FAM126B | 7304 | 0.050804574 | 0.27977818 | No |
| row_76 | SNX9 | 7319 | 0.050053325 | 0.27954888 | No |
| row_77 | PRNP | 7325 | 0.049863398 | 0.27985266 | No |
| row_78 | XBP1 | 7413 | 0.046398114 | 0.2752369 | No |
| row_79 | ITGAE | 7538 | 0.041017376 | 0.26835534 | No |
| row_80 | PIM1 | 7744 | 0.032692086 | 0.2565551 | No |
| row_81 | P4HA1 | 7758 | 0.032196775 | 0.25617 | No |
| row_82 | PTRH2 | 7989 | 0.023069683 | 0.24276665 | No |
| row_83 | NCS1 | 8009 | 0.022206837 | 0.24190418 | No |
| row_84 | SPP1 | 8235 | 0.014217005 | 0.2286915 | No |
| row_85 | AHR | 8294 | 0.01165055 | 0.22538185 | No |
| row_86 | SWAP70 | 8579 | 9.94E-04 | 0.20850015 | No |
| row_87 | GSTO1 | 8670 | -0.002602112 | 0.20317788 | No |
| row_88 | CAPG | 8716 | -0.003987202 | 0.20054913 | No |
| row_89 | PLAGL1 | 8733 | -0.004712603 | 0.19965419 | No |
| row_90 | CDC6 | 9043 | -0.016376229 | 0.18147083 | No |
| row_91 | ENO3 | 9249 | -0.023466175 | 0.16955936 | No |
| row_92 | CSF1 | 9323 | -0.026080659 | 0.16553143 | No |
| row_93 | PHLDA1 | 9443 | -0.02990241 | 0.15881325 | No |
| row_94 | MYC | 9668 | -0.037797388 | 0.14594437 | No |
| row_95 | ARL4A | 9675 | -0.038107831 | 0.14604694 | No |
| row_96 | ODC1 | 9768 | -0.04146241 | 0.14107424 | No |
| row_97 | TGM2 | 9858 | -0.044342678 | 0.13631473 | No |
| row_98 | MAPKAPK2 | 10203 | -0.058075644 | 0.11655217 | No |
| row_99 | BMPR2 | 10304 | -0.062338766 | 0.111355305 | No |
| row_100 | SLC29A2 | 10617 | -0.073439263 | 0.09368149 | No |
| row_101 | CA2 | 10630 | -0.073974937 | 0.09385959 | No |
| row_102 | IL10 | 10763 | -0.079366758 | 0.086964525 | No |
| row_103 | CD83 | 10860 | -0.082840241 | 0.082252786 | No |
| row_104 | SOCS2 | 10877 | -0.083169341 | 0.08230381 | No |
| row_105 | LCLAT1 | 10982 | -0.087805085 | 0.07717606 | No |
| row_106 | AMACR | 11155 | -0.095698014 | 0.0680985 | No |
| row_107 | FAH | 11193 | -0.09720964 | 0.06706963 | No |
| row_108 | CXCL10 | 11427 | -0.106558509 | 0.054494437 | No |
| row_109 | GPX4 | 11491 | -0.109419547 | 0.052066173 | No |
| row_110 | CCR4 | 11494 | -0.109527782 | 0.05326779 | No |
| row_111 | TNFRSF1B | 11631 | -0.115914211 | 0.046575435 | No |
| row_112 | CASP3 | 11892 | -0.127560675 | 0.03264738 | No |
| row_113 | CDCP1 | 11905 | -0.128692448 | 0.03348522 | No |
| row_114 | PNP | 11928 | -0.129799291 | 0.033741552 | No |
| row_115 | NOP2 | 12112 | -0.138797283 | 0.024529312 | No |
| row_116 | CAPN3 | 12139 | -0.140271291 | 0.024673969 | No |
| row_117 | DCPS | 12141 | -0.140302852 | 0.026306126 | No |
| row_118 | SPRED2 | 12190 | -0.142500967 | 0.025168998 | No |
| row_119 | CD81 | 12245 | -0.145282567 | 0.0237085 | No |
| row_120 | MYO1E | 12297 | -0.1479031 | 0.022458052 | No |
| row_121 | UCK2 | 12391 | -0.152210563 | 0.018761175 | No |
| row_122 | BHLHE40 | 12557 | -0.161105901 | 0.010888641 | No |
| row_123 | FURIN | 12583 | -0.162668303 | 0.011362824 | No |
| row_124 | SPRY4 | 12590 | -0.162751526 | 0.012968224 | No |
| row_125 | IL4R | 12788 | -0.173999175 | 0.003347628 | No |
| row_126 | CTSZ | 12823 | -0.175829962 | 0.003445139 | No |
| row_127 | HK2 | 12836 | -0.176386043 | 0.004858022 | No |
| row_128 | HOPX | 12857 | -0.177684397 | 0.00581068 | No |
| row_129 | SLC39A8 | 12902 | -0.180233687 | 0.005366439 | No |
| row_130 | CDC42SE2 | 12908 | -0.180467173 | 0.007244922 | No |
| row_131 | IL1R2 | 12983 | -0.184928745 | 0.005072743 | No |
| row_132 | RABGAP1L | 13074 | -0.190100506 | 0.002011163 | No |
| row_133 | LRIG1 | 13243 | -0.200532869 | -0.005564452 | No |
| row_134 | TNFRSF8 | 13266 | -0.202227324 | -0.004434848 | No |
| row_135 | MUC1 | 13268 | -0.20223701 | -0.002055944 | No |
| row_136 | FLT3LG | 13302 | -0.204608351 | -0.001551965 | No |
| row_137 | PLSCR1 | 13332 | -0.20686014 | -7.83E-04 | No |
| row_138 | CISH | 13358 | -0.208618969 | 2.45E-04 | No |
| row_139 | PUS1 | 13423 | -0.212803885 | -9.96E-04 | No |
| row_140 | P2RX4 | 13446 | -0.214590743 | 2.83E-04 | No |
| row_141 | SMPDL3A | 13504 | -0.21976687 | -4.58E-04 | No |
| row_142 | SYT11 | 13605 | -0.22918728 | -0.003643293 | No |
| row_143 | NRP1 | 13626 | -0.230588749 | -0.002052763 | No |
| row_144 | APLP1 | 14068 | -0.27135843 | -0.025013795 | No |
| row_145 | LIF | 14070 | -0.271922886 | -0.021794684 | No |
| row_146 | ECM1 | 14118 | -0.276274204 | -0.021259412 | No |
| row_147 | CCNE1 | 14258 | -0.290272862 | -0.026027963 | No |
| row_148 | COL6A1 | 14313 | -0.297200292 | -0.025656777 | No |
| row_149 | CDKN1C | 14668 | -0.340517431 | -0.04260877 | No |
| row_150 | NT5E | 14722 | -0.350571573 | -0.0415346 | No |
| row_151 | NFKBIZ | 14761 | -0.356567711 | -0.039495856 | No |
| row_152 | MAP3K8 | 14862 | -0.372637212 | -0.040951427 | No |
| row_153 | GALM | 14879 | -0.375216156 | -0.03737917 | No |
| row_154 | SOCS1 | 14906 | -0.38038069 | -0.034339495 | No |
| row_155 | ETV4 | 15019 | -0.399627477 | -0.03618346 | No |
| row_156 | PHTF2 | 15104 | -0.415073425 | -0.036175612 | No |
| row_157 | TWSG1 | 15195 | -0.434531778 | -0.036290064 | No |
| row_158 | CCND3 | 15219 | -0.43960768 | -0.03235783 | No |
| row_159 | IFITM3 | 15365 | -0.471685678 | -0.035295982 | No |
| row_160 | LRRC8C | 15483 | -0.505468369 | -0.036161236 | No |
| row_161 | ITIH5 | 15532 | -0.521914423 | -0.032723747 | No |
| row_162 | ITGA6 | 15558 | -0.531347752 | -0.027804364 | No |
| row_163 | TNFRSF18 | 15567 | -0.534395933 | -0.021836987 | No |
| row_164 | IL3RA | 15755 | -0.593194962 | -0.025808455 | No |
| row_165 | RORA | 15836 | -0.628019631 | -0.02299516 | No |
| row_166 | PTGER2 | 15918 | -0.670851052 | -0.019724928 | No |
| row_167 | CCND2 | 15925 | -0.672859251 | -0.011969117 | No |
| row_168 | GLIPR2 | 16001 | -0.704795003 | -0.00793271 | No |
| row_169 | PTCH1 | 16154 | -0.789741695 | -0.007452428 | No |
| row_170 | IL1RL1 | 16665 | -1.479965806 | -0.019945636 | No |
| row_171 | SERPINC1 | 16912 | -3.213284969 | 0.004163927 | No |

ESO26 Inflammatory Response

| NAME | SYMBOL | RANK IN GENE LIST | RANK METRIC SCORE | RUNNING ES | CORE ENRICHMENT |
| --- | --- | --- | --- | --- | --- |
| row_0 | IL1A | 181 | 2.590133667 | 0.031455886 | Yes |
| row_1 | NLRP3 | 471 | 1.70213747 | 0.042013682 | Yes |
| row_2 | SELE | 520 | 1.608354926 | 0.065376066 | Yes |
| row_3 | CCL2 | 535 | 1.585931063 | 0.09039505 | Yes |
| row_4 | CALCRL | 586 | 1.484196663 | 0.11161462 | Yes |
| row_5 | FPR1 | 605 | 1.446271896 | 0.13411918 | Yes |
| row_6 | ROS1 | 661 | 1.368083119 | 0.15314867 | Yes |
| row_7 | SCN1B | 748 | 1.259665847 | 0.16856718 | Yes |
| row_8 | SELL | 767 | 1.225902677 | 0.18747959 | Yes |
| row_9 | C3AR1 | 768 | 1.223286271 | 0.20741989 | Yes |
| row_10 | IL18RAP | 817 | 1.16795814 | 0.22360353 | Yes |
| row_11 | PTGIR | 837 | 1.147836328 | 0.24118394 | Yes |
| row_12 | MARCO | 841 | 1.145837784 | 0.25968337 | Yes |
| row_13 | AQP9 | 846 | 1.139349699 | 0.27801755 | Yes |
| row_14 | HAS2 | 881 | 1.112376094 | 0.29412782 | Yes |
| row_15 | CXCL6 | 932 | 1.063996553 | 0.30849788 | Yes |
| row_16 | ITGB3 | 972 | 1.028929949 | 0.32295054 | Yes |
| row_17 | PDE4B | 980 | 1.016637444 | 0.33910602 | Yes |
| row_18 | EDN1 | 1020 | 0.990911841 | 0.35293898 | Yes |
| row_19 | IL10RA | 1068 | 0.960665703 | 0.36580312 | Yes |
| row_20 | LCK | 1146 | 0.910015047 | 0.3760574 | Yes |
| row_21 | MMP14 | 1194 | 0.876987159 | 0.3875575 | Yes |
| row_22 | CCL24 | 1276 | 0.82311213 | 0.39615732 | Yes |
| row_23 | IL7R | 1383 | 0.770652235 | 0.40241513 | Yes |
| row_24 | CYBB | 1419 | 0.75518012 | 0.41264343 | Yes |
| row_25 | CCL7 | 1513 | 0.707209766 | 0.41864026 | Yes |
| row_26 | GPR132 | 1515 | 0.706384599 | 0.4300953 | Yes |
| row_27 | PDPN | 1594 | 0.677757919 | 0.43650413 | Yes |
| row_28 | GP1BA | 1848 | 0.591380894 | 0.431097 | Yes |
| row_29 | TNFRSF9 | 1889 | 0.583548665 | 0.43823025 | Yes |
| row_30 | HBEGF | 2111 | 0.511701524 | 0.43342745 | Yes |
| row_31 | GPC3 | 2351 | 0.451443315 | 0.4265719 | Yes |
| row_32 | SERPINE1 | 2438 | 0.436262757 | 0.42856848 | Yes |
| row_33 | SLC28A2 | 2444 | 0.435605168 | 0.43537173 | Yes |
| row_34 | CD14 | 2481 | 0.427575111 | 0.4402004 | Yes |
| row_35 | EBI3 | 2495 | 0.424481928 | 0.44634652 | Yes |
| row_36 | DCBLD2 | 2534 | 0.417036057 | 0.45088443 | Yes |
| row_37 | EMP3 | 2604 | 0.404841125 | 0.45337987 | Yes |
| row_38 | IRAK2 | 2805 | 0.368634045 | 0.44749397 | Yes |
| row_39 | EREG | 3128 | 0.326034516 | 0.43365782 | Yes |
| row_40 | TNFSF9 | 3131 | 0.325743049 | 0.43884867 | Yes |
| row_41 | CCL22 | 3164 | 0.321075469 | 0.44217923 | Yes |
| row_42 | ICAM1 | 3252 | 0.308981627 | 0.44204155 | Yes |
| row_43 | F3 | 3308 | 0.301742136 | 0.44368905 | Yes |
| row_44 | P2RX7 | 3312 | 0.301502705 | 0.4484253 | Yes |
| row_45 | PTAFR | 3377 | 0.296460778 | 0.44945142 | Yes |
| row_46 | GNA15 | 3385 | 0.295762062 | 0.4538562 | Yes |
| row_47 | CX3CL1 | 3456 | 0.289358407 | 0.45440972 | Yes |
| row_48 | LDLR | 3479 | 0.286428154 | 0.45777023 | Yes |
| row_49 | ABCA1 | 3505 | 0.282620817 | 0.46089026 | Yes |
| row_50 | TLR1 | 3531 | 0.279919714 | 0.46396628 | Yes |
| row_51 | SLC7A2 | 3547 | 0.278562665 | 0.4676149 | Yes |
| row_52 | BDKRB1 | 3717 | 0.264264792 | 0.46187142 | Yes |
| row_53 | SLC7A1 | 3732 | 0.262140065 | 0.46531183 | Yes |
| row_54 | BEST1 | 3834 | 0.253459543 | 0.46343645 | Yes |
| row_55 | KIF1B | 3940 | 0.243039459 | 0.46115336 | Yes |
| row_56 | RASGRP1 | 3995 | 0.236407101 | 0.46179533 | Yes |
| row_57 | CD40 | 4075 | 0.230154112 | 0.4608485 | Yes |
| row_58 | CD55 | 4116 | 0.22675699 | 0.4621658 | Yes |
| row_59 | IL15RA | 4144 | 0.224882066 | 0.4642257 | Yes |
| row_60 | RIPK2 | 4189 | 0.221887097 | 0.46522576 | Yes |
| row_61 | IL15 | 4198 | 0.221385464 | 0.46835867 | Yes |
| row_62 | MET | 4588 | 0.191028699 | 0.44833708 | No |
| row_63 | CXCL11 | 4661 | 0.186077669 | 0.4470881 | No |
| row_64 | SLC4A4 | 4685 | 0.184170097 | 0.44872227 | No |
| row_65 | PTGER4 | 4751 | 0.179649353 | 0.44778484 | No |
| row_66 | HRH1 | 4815 | 0.175898403 | 0.4469052 | No |
| row_67 | IL1R1 | 4894 | 0.170885369 | 0.44505176 | No |
| row_68 | ICAM4 | 4917 | 0.168846428 | 0.44649562 | No |
| row_69 | NFKBIA | 4961 | 0.165857896 | 0.4466418 | No |
| row_70 | RGS16 | 4981 | 0.164758131 | 0.44819745 | No |
| row_71 | TNFSF15 | 5035 | 0.161221743 | 0.44767332 | No |
| row_72 | KLF6 | 5087 | 0.158143327 | 0.44721797 | No |
| row_73 | NFKB1 | 5147 | 0.153656781 | 0.4462137 | No |
| row_74 | KCNJ2 | 5278 | 0.146376505 | 0.44086805 | No |
| row_75 | IFNGR2 | 5348 | 0.141918957 | 0.4390777 | No |
| row_76 | IL18R1 | 5380 | 0.140262753 | 0.43952036 | No |
| row_77 | STAB1 | 5422 | 0.138128772 | 0.4393335 | No |
| row_78 | ITGB8 | 5534 | 0.132284269 | 0.43488815 | No |
| row_79 | C5AR1 | 5852 | 0.116040036 | 0.41792634 | No |
| row_80 | OLR1 | 5855 | 0.115921825 | 0.419697 | No |
| row_81 | CD82 | 5949 | 0.110622399 | 0.4159691 | No |
| row_82 | IL6 | 5975 | 0.109609604 | 0.41626894 | No |
| row_83 | PLAUR | 6035 | 0.107005179 | 0.41450423 | No |
| row_84 | ATP2C1 | 6087 | 0.10432516 | 0.4131716 | No |
| row_85 | MXD1 | 6235 | 0.096740119 | 0.4060058 | No |
| row_86 | BST2 | 6371 | 0.090660401 | 0.3994546 | No |
| row_87 | BTG2 | 6456 | 0.086466372 | 0.3958682 | No |
| row_88 | SLC31A1 | 6471 | 0.085656136 | 0.39643183 | No |
| row_89 | ACVR2A | 6502 | 0.084661715 | 0.39602762 | No |
| row_90 | TNFSF10 | 6605 | 0.079443641 | 0.39125624 | No |
| row_91 | SGMS2 | 6635 | 0.078518152 | 0.39081138 | No |
| row_92 | CCL20 | 6721 | 0.074960612 | 0.38697797 | No |
| row_93 | ADRM1 | 6800 | 0.071941197 | 0.38351166 | No |
| row_94 | TACR1 | 6877 | 0.068312317 | 0.38010514 | No |
| row_95 | PSEN1 | 7175 | 0.055860486 | 0.36335185 | No |
| row_96 | RNF144B | 7350 | 0.048803296 | 0.35379887 | No |
| row_97 | ABI1 | 7390 | 0.047436211 | 0.3522526 | No |
| row_98 | OSMR | 7446 | 0.045155089 | 0.34971756 | No |
| row_99 | IL1B | 7496 | 0.042746071 | 0.34750012 | No |
| row_100 | ACVR1B | 7616 | 0.038347807 | 0.34104776 | No |
| row_101 | IL18 | 7720 | 0.033852331 | 0.33547375 | No |
| row_102 | TLR3 | 7921 | 0.025996001 | 0.32400265 | No |
| row_103 | ATP2A2 | 7948 | 0.024583535 | 0.32285705 | No |
| row_104 | GNAI3 | 8062 | 0.019721944 | 0.31645793 | No |
| row_105 | NAMPT | 8174 | 0.016176211 | 0.31011996 | No |
| row_106 | MEP1A | 8194 | 0.015490053 | 0.30924246 | No |
| row_107 | AHR | 8294 | 0.01165055 | 0.3035444 | No |
| row_108 | CD69 | 8992 | -0.014028252 | 0.26231954 | No |
| row_109 | TNFAIP6 | 9026 | -0.015448892 | 0.2606087 | No |
| row_110 | RAF1 | 9088 | -0.017910274 | 0.25727272 | No |
| row_111 | SEMA4D | 9116 | -0.019012488 | 0.25597683 | No |
| row_112 | TAPBP | 9254 | -0.023693079 | 0.24821508 | No |
| row_113 | LAMP3 | 9299 | -0.025109081 | 0.2460075 | No |
| row_114 | CSF1 | 9323 | -0.026080659 | 0.24506472 | No |
| row_115 | SCARF1 | 9476 | -0.031434596 | 0.23653704 | No |
| row_116 | IRF1 | 9569 | -0.034941316 | 0.23163497 | No |
| row_117 | ADM | 9599 | -0.035636816 | 0.23049112 | No |
| row_118 | MYC | 9668 | -0.037797388 | 0.227063 | No |
| row_119 | ICOSLG | 10228 | -0.05906596 | 0.1947797 | No |
| row_120 | CCL5 | 10465 | -0.068143778 | 0.18185456 | No |
| row_121 | SLC31A2 | 10579 | -0.072183579 | 0.1763106 | No |
| row_122 | IL10 | 10763 | -0.079366758 | 0.16672054 | No |
| row_123 | RELA | 11172 | -0.096260414 | 0.14402415 | No |
| row_124 | HIF1A | 11307 | -0.101846911 | 0.13771477 | No |
| row_125 | IFNAR1 | 11396 | -0.105524749 | 0.13420115 | No |
| row_126 | LYN | 11402 | -0.105692357 | 0.13562663 | No |
| row_127 | TLR2 | 11404 | -0.105786785 | 0.13729154 | No |
| row_128 | CXCL10 | 11427 | -0.106558509 | 0.13772008 | No |
| row_129 | SRI | 11469 | -0.108390287 | 0.13704845 | No |
| row_130 | RHOG | 11527 | -0.111117661 | 0.1354697 | No |
| row_131 | TNFRSF1B | 11631 | -0.115914211 | 0.13123333 | No |
| row_132 | TIMP1 | 11662 | -0.116896994 | 0.1313546 | No |
| row_133 | ITGA5 | 11684 | -0.117888175 | 0.13202728 | No |
| row_134 | NMI | 11721 | -0.119720958 | 0.13183774 | No |
| row_135 | CXCL9 | 11750 | -0.121234506 | 0.13214865 | No |
| row_136 | ADORA2B | 11772 | -0.122330867 | 0.13289376 | No |
| row_137 | P2RY2 | 11906 | -0.128702641 | 0.12708162 | No |
| row_138 | LPAR1 | 11998 | -0.133566201 | 0.123846665 | No |
| row_139 | TPBG | 12294 | -0.147858471 | 0.10871194 | No |
| row_140 | EIF2AK2 | 12360 | -0.150781974 | 0.10730395 | No |
| row_141 | SPHK1 | 12586 | -0.162692532 | 0.09657423 | No |
| row_142 | IL4R | 12788 | -0.173999175 | 0.0874562 | No |
| row_143 | SLC11A2 | 12833 | -0.176305279 | 0.08771321 | No |
| row_144 | PTPRE | 12914 | -0.180872053 | 0.08590359 | No |
| row_145 | RTP4 | 13000 | -0.185634106 | 0.083874226 | No |
| row_146 | ATP2B1 | 13045 | -0.188449562 | 0.0843292 | No |
| row_147 | PVR | 13181 | -0.196709976 | 0.079506665 | No |
| row_148 | CSF3 | 13330 | -0.206834361 | 0.074076004 | No |
| row_149 | P2RX4 | 13446 | -0.214590743 | 0.07073442 | No |
| row_150 | GCH1 | 13459 | -0.215591416 | 0.073534995 | No |
| row_151 | LY6E | 13719 | -0.237709403 | 0.062005974 | No |
| row_152 | CCRL2 | 13751 | -0.239841118 | 0.06407183 | No |
| row_153 | FFAR2 | 13915 | -0.256111234 | 0.058552288 | No |
| row_154 | LIF | 14070 | -0.271922886 | 0.05382576 | No |
| row_155 | FZD5 | 14099 | -0.274476051 | 0.0566346 | No |
| row_156 | PCDH7 | 14424 | -0.311903417 | 0.042449158 | No |
| row_157 | NOD2 | 14567 | -0.328593224 | 0.03936008 | No |
| row_158 | MEFV | 14839 | -0.37051329 | 0.029282145 | No |
| row_159 | IFITM1 | 15196 | -0.43466866 | 0.01519467 | No |
| row_160 | AXL | 15253 | -0.444943339 | 0.019116955 | No |
| row_161 | IRF7 | 15316 | -0.457957298 | 0.022894528 | No |
| row_162 | GABBR1 | 15363 | -0.470942587 | 0.027835358 | No |
| row_163 | HPN | 15844 | -0.630272031 | 0.009561528 | No |
| row_164 | INHBA | 15893 | -0.656004071 | 0.01740002 | No |
| row_165 | PTGER2 | 15918 | -0.670851052 | 0.026907912 | No |
| row_166 | CDKN1A | 15940 | -0.678551853 | 0.03671975 | No |
| row_167 | SLC1A2 | 15961 | -0.690007389 | 0.0467778 | No |
| row_168 | LCP2 | 16234 | -0.851851463 | 0.04448649 | No |

ESO26 Allograft Rejection

| NAME | SYMBOL | RANK IN GENE LIST | RANK METRIC SCORE | RUNNING ES | CORE ENRICHMENT |
| --- | --- | --- | --- | --- | --- |
| row_0 | ITK | 136 | 3.136317492 | 0.046341036 | Yes |
| row_1 | THY1 | 206 | 2.430032253 | 0.084406465 | Yes |
| row_2 | BCAT1 | 268 | 2.186154366 | 0.1187155 | Yes |
| row_3 | CD3G | 280 | 2.1692698 | 0.15570106 | Yes |
| row_4 | CD4 | 409 | 1.812833905 | 0.17955355 | Yes |
| row_5 | NLRP3 | 471 | 1.70213747 | 0.20546445 | Yes |
| row_6 | MAP4K1 | 490 | 1.682004452 | 0.23357977 | Yes |
| row_7 | CCL2 | 535 | 1.585931063 | 0.258484 | Yes |
| row_8 | HCLS1 | 551 | 1.554904342 | 0.28457218 | Yes |
| row_9 | IL13 | 621 | 1.412258029 | 0.30497828 | Yes |
| row_10 | ITGAL | 673 | 1.361057878 | 0.32556504 | Yes |
| row_11 | IL18RAP | 817 | 1.16795814 | 0.3373375 | Yes |
| row_12 | IL2RG | 1088 | 0.947733462 | 0.33774638 | Yes |
| row_13 | LCK | 1146 | 0.910015047 | 0.3501508 | Yes |
| row_14 | IL12RB1 | 1330 | 0.797176778 | 0.35311428 | Yes |
| row_15 | CCL7 | 1513 | 0.707209766 | 0.35457614 | Yes |
| row_16 | C2 | 1556 | 0.69169575 | 0.36408332 | Yes |
| row_17 | ST8SIA4 | 1781 | 0.611909807 | 0.3613973 | Yes |
| row_18 | ETS1 | 1786 | 0.610253572 | 0.37174818 | Yes |
| row_19 | MMP9 | 1921 | 0.575649977 | 0.37377807 | Yes |
| row_20 | ACHE | 1932 | 0.571406126 | 0.3830986 | Yes |
| row_21 | CD247 | 2050 | 0.527474523 | 0.3853022 | Yes |
| row_22 | PRKCG | 2094 | 0.515997112 | 0.39170146 | Yes |
| row_23 | CD8A | 2126 | 0.508684218 | 0.39868653 | Yes |
| row_24 | APBB1 | 2135 | 0.506440461 | 0.40699863 | Yes |
| row_25 | ITGB2 | 2282 | 0.466932923 | 0.4064295 | Yes |
| row_26 | WAS | 2319 | 0.457996339 | 0.41223812 | Yes |
| row_27 | CSK | 2356 | 0.451003075 | 0.41792542 | Yes |
| row_28 | HLA-A | 2377 | 0.448920518 | 0.4245268 | Yes |
| row_29 | HLA-DQA1 | 2453 | 0.434143901 | 0.42760542 | Yes |
| row_30 | CCL4 | 2519 | 0.420571238 | 0.4310424 | Yes |
| row_31 | IL12A | 2775 | 0.372321934 | 0.4223582 | Yes |
| row_32 | HDAC9 | 2970 | 0.341929674 | 0.41676944 | Yes |
| row_33 | LTB | 3014 | 0.337170929 | 0.4200659 | Yes |
| row_34 | F2 | 3087 | 0.329307169 | 0.42150366 | Yes |
| row_35 | EREG | 3128 | 0.326034516 | 0.42478508 | Yes |
| row_36 | FLNA | 3133 | 0.325361371 | 0.43019286 | Yes |
| row_37 | CCL22 | 3164 | 0.321075469 | 0.4339821 | Yes |
| row_38 | ICAM1 | 3252 | 0.308981627 | 0.43417636 | Yes |
| row_39 | TLR1 | 3531 | 0.279919714 | 0.42252293 | No |
| row_40 | TLR6 | 3611 | 0.272958785 | 0.42256725 | No |
| row_41 | NCF4 | 3720 | 0.264001548 | 0.42073387 | No |
| row_42 | NCK1 | 4034 | 0.233399749 | 0.40619466 | No |
| row_43 | CD40 | 4075 | 0.230154112 | 0.40781248 | No |
| row_44 | IKBKB | 4167 | 0.22299403 | 0.40627718 | No |
| row_45 | RIPK2 | 4189 | 0.221887097 | 0.40887994 | No |
| row_46 | IL15 | 4198 | 0.221385464 | 0.41224608 | No |
| row_47 | EIF4G3 | 4235 | 0.217919037 | 0.41388914 | No |
| row_48 | IFNGR1 | 4616 | 0.189266667 | 0.3946051 | No |
| row_49 | AKT1 | 4714 | 0.182110444 | 0.3920041 | No |
| row_50 | UBE2D1 | 4739 | 0.18077217 | 0.39371532 | No |
| row_51 | MTIF2 | 5165 | 0.152420044 | 0.3711194 | No |
| row_52 | IFNGR2 | 5348 | 0.141918957 | 0.36277297 | No |
| row_53 | STAB1 | 5422 | 0.138128772 | 0.36083418 | No |
| row_54 | F2R | 5742 | 0.122277506 | 0.34401056 | No |
| row_55 | IL6 | 5975 | 0.109609604 | 0.33213404 | No |
| row_56 | EGFR | 6391 | 0.089922749 | 0.30904764 | No |
| row_57 | HLA-DRA | 6423 | 0.087850392 | 0.30873087 | No |
| row_58 | ACVR2A | 6502 | 0.084661715 | 0.30556744 | No |
| row_59 | CD74 | 6698 | 0.075925991 | 0.29530388 | No |
| row_60 | KLRD1 | 6835 | 0.069900334 | 0.28843975 | No |
| row_61 | LY75 | 6859 | 0.069025204 | 0.28827146 | No |
| row_62 | IRF8 | 6896 | 0.067548446 | 0.28730544 | No |
| row_63 | EIF3A | 6973 | 0.064108856 | 0.2839042 | No |
| row_64 | GBP2 | 7004 | 0.062479611 | 0.28320658 | No |
| row_65 | JAK2 | 7347 | 0.04903407 | 0.26374617 | No |
| row_66 | ABI1 | 7390 | 0.047436211 | 0.2620749 | No |
| row_67 | IFNAR2 | 7465 | 0.04443777 | 0.2584511 | No |
| row_68 | IL1B | 7496 | 0.042746071 | 0.2574111 | No |
| row_69 | IL18 | 7720 | 0.033852331 | 0.24475461 | No |
| row_70 | TLR3 | 7921 | 0.025996001 | 0.23332778 | No |
| row_71 | BCL10 | 8313 | 0.010882451 | 0.2102953 | No |
| row_72 | ABCE1 | 8412 | 0.006811799 | 0.20459333 | No |
| row_73 | GALNT1 | 8542 | 0.002513671 | 0.19697571 | No |
| row_74 | CDKN2A | 8665 | -0.002403379 | 0.18977189 | No |
| row_75 | CAPG | 8716 | -0.003987202 | 0.18687159 | No |
| row_76 | NOS2 | 9151 | -0.020031963 | 0.16144413 | No |
| row_77 | TAPBP | 9254 | -0.023693079 | 0.15579751 | No |
| row_78 | CSF1 | 9323 | -0.026080659 | 0.15221155 | No |
| row_79 | TAP1 | 9346 | -0.026939288 | 0.1513724 | No |
| row_80 | GLMN | 9448 | -0.030185893 | 0.1458978 | No |
| row_81 | EIF3D | 9625 | -0.036413081 | 0.13607706 | No |
| row_82 | MAP3K7 | 9632 | -0.036579501 | 0.13635541 | No |
| row_83 | ELF4 | 9766 | -0.04130673 | 0.12917332 | No |
| row_84 | RPL39 | 10195 | -0.057629976 | 0.10475456 | No |
| row_85 | ICOSLG | 10228 | -0.05906596 | 0.10387895 | No |
| row_86 | DYRK3 | 10274 | -0.061265942 | 0.10226944 | No |
| row_87 | RPL9 | 10321 | -0.062980242 | 0.10063029 | No |
| row_88 | CCL5 | 10465 | -0.068143778 | 0.09331995 | No |
| row_89 | NME1 | 10479 | -0.068740271 | 0.0937406 | No |
| row_90 | FAS | 10485 | -0.069115587 | 0.09464287 | No |
| row_91 | MRPL3 | 10516 | -0.070138104 | 0.094078146 | No |
| row_92 | BCL3 | 10554 | -0.071314454 | 0.09311811 | No |
| row_93 | BRCA1 | 10555 | -0.071317635 | 0.09435554 | No |
| row_94 | IL10 | 10763 | -0.079366758 | 0.083439 | No |
| row_95 | PSMB10 | 10885 | -0.083457522 | 0.077700935 | No |
| row_96 | STAT4 | 10902 | -0.084079273 | 0.07820956 | No |
| row_97 | EIF3J | 11061 | -0.09134718 | 0.07041098 | No |
| row_98 | EIF5A | 11078 | -0.092454433 | 0.07106492 | No |
| row_99 | HIF1A | 11307 | -0.101846911 | 0.059291255 | No |
| row_100 | TRAF2 | 11319 | -0.102067724 | 0.060408942 | No |
| row_101 | LYN | 11402 | -0.105692357 | 0.057372864 | No |
| row_102 | TLR2 | 11404 | -0.105786785 | 0.059148975 | No |
| row_103 | RPS3A | 11407 | -0.105896376 | 0.060867593 | No |
| row_104 | IL11 | 11445 | -0.10750661 | 0.060535524 | No |
| row_105 | UBE2N | 11638 | -0.116137601 | 0.051147837 | No |
| row_106 | CD96 | 11644 | -0.116329156 | 0.05286931 | No |
| row_107 | RPS19 | 11660 | -0.116825335 | 0.054005492 | No |
| row_108 | TIMP1 | 11662 | -0.116896994 | 0.055974375 | No |
| row_109 | CXCL9 | 11750 | -0.121234506 | 0.05291102 | No |
| row_110 | CD47 | 11903 | -0.128548071 | 0.046114247 | No |
| row_111 | CTSS | 11921 | -0.129487529 | 0.047351357 | No |
| row_112 | IL27RA | 11933 | -0.129961684 | 0.048953027 | No |
| row_113 | TPD52 | 11963 | -0.131442398 | 0.04951138 | No |
| row_114 | NPM1 | 12386 | -0.152052298 | 0.027087267 | No |
| row_115 | PTPN6 | 12523 | -0.15938209 | 0.021775726 | No |
| row_116 | B2M | 12531 | -0.15968971 | 0.024130763 | No |
| row_117 | IL4R | 12788 | -0.173999175 | 0.011946104 | No |
| row_118 | TAP2 | 13003 | -0.18594256 | 0.002463031 | No |
| row_119 | SOCS5 | 13020 | -0.186855376 | 0.004754913 | No |
| row_120 | RPS9 | 13104 | -0.19158788 | 0.003149813 | No |
| row_121 | STAT1 | 13276 | -0.202926978 | -0.003484818 | No |
| row_122 | CD8B | 13832 | -0.248427644 | -0.032135528 | No |
| row_123 | DEGS1 | 13854 | -0.250141829 | -0.029042514 | No |
| row_124 | LIF | 14070 | -0.271922886 | -0.03709314 | No |
| row_125 | CXCR3 | 14484 | -0.318611801 | -0.056092784 | No |
| row_126 | CD7 | 14698 | -0.34501636 | -0.06275639 | No |
| row_127 | SOCS1 | 14906 | -0.38038069 | -0.068450056 | No |
| row_128 | ZAP70 | 15152 | -0.427384347 | -0.07558496 | No |
| row_129 | CFP | 15205 | -0.436580896 | -0.071098134 | No |
| row_130 | CCND3 | 15219 | -0.43960768 | -0.0642426 | No |
| row_131 | IRF7 | 15316 | -0.457957298 | -0.061998006 | No |
| row_132 | IL7 | 15324 | -0.460439742 | -0.05442468 | No |
| row_133 | INHBB | 15337 | -0.465509474 | -0.047060333 | No |
| row_134 | TGFB2 | 15338 | -0.465615273 | -0.03898148 | No |
| row_135 | PRF1 | 15377 | -0.474483132 | -0.033005558 | No |
| row_136 | TGFB1 | 15499 | -0.510027289 | -0.031342242 | No |
| row_137 | INHBA | 15893 | -0.656004071 | -0.04330003 | No |
| row_138 | CCND2 | 15925 | -0.672859251 | -0.033466373 | No |
| row_139 | SRGN | 15989 | -0.698740542 | -0.025084116 | No |
| row_140 | GCNT1 | 16004 | -0.705694854 | -0.013671111 | No |
| row_141 | CRTAM | 16200 | -0.823733985 | -0.010959514 | No |
| row_142 | LCP2 | 16234 | -0.851851463 | 0.001861043 | No |
| row_143 | CCR1 | 16419 | -1.014840961 | 0.008541808 | No |
| row_144 | IL16 | 16645 | -1.431350112 | 0.02001442 | No |

EAC E2F Targets

| NAME | GENE SYMBOL | RANK IN GENE LIST | RANK METRIC SCORE | RUNNING ES | CORE ENRICHMENT |
| --- | --- | --- | --- | --- | --- |
| row_0 | CDKN1A | 2167 | 0.390260339 | -0.02338772 | No |
| row_1 | TP53 | 4441 | 0.285671502 | -0.053002372 | No |
| row_2 | CDKN2C | 5657 | 0.249946758 | -0.06483591 | No |
| row_3 | LBR | 7489 | 0.2079207 | -0.08960418 | No |
| row_4 | CCP110 | 9901 | 0.163270041 | -0.12676002 | No |
| row_5 | CKS2 | 14058 | 0.107323766 | -0.19796377 | No |
| row_6 | SMC3 | 14316 | 0.104464948 | -0.19834638 | No |
| row_7 | WEE1 | 14965 | 0.097851336 | -0.20611465 | No |
| row_8 | RAD50 | 16240 | 0.084750369 | -0.22581084 | No |
| row_9 | PAN2 | 16747 | 0.079735495 | -0.23174009 | No |
| row_10 | HMGB2 | 16795 | 0.079318598 | -0.2293354 | No |
| row_11 | HUS1 | 17274 | 0.074747078 | -0.23496023 | No |
| row_12 | KIF22 | 17462 | 0.072995104 | -0.23536257 | No |
| row_13 | MXD3 | 17644 | 0.071279913 | -0.23572627 | No |
| row_14 | CIT | 18088 | 0.067499474 | -0.24101216 | No |
| row_15 | PSIP1 | 18533 | 0.063769378 | -0.24646954 | No |
| row_16 | POLA2 | 18886 | 0.060865097 | -0.25037244 | No |
| row_17 | MMS22L | 18993 | 0.059932116 | -0.24983793 | No |
| row_18 | POLE | 19220 | 0.058060665 | -0.25156364 | No |
| row_19 | NUP205 | 19472 | 0.056231789 | -0.25381935 | No |
| row_20 | STAG1 | 20515 | 0.048424669 | -0.27078742 | No |
| row_21 | POLD1 | 20930 | 0.045148041 | -0.27646428 | No |
| row_22 | CDK1 | 21332 | 0.042195763 | -0.28202593 | No |
| row_23 | LYAR | 21673 | 0.039418545 | -0.28659192 | No |
| row_24 | PRKDC | 21983 | 0.037207104 | -0.29068476 | No |
| row_25 | AK2 | 22458 | 0.034067623 | -0.29790863 | No |
| row_26 | AURKA | 22826 | 0.03180695 | -0.30327865 | No |
| row_27 | CHEK1 | 23197 | 0.029314717 | -0.30880567 | No |
| row_28 | RAD21 | 23310 | 0.028615139 | -0.3096674 | No |
| row_29 | TFRC | 23468 | 0.027622376 | -0.31138867 | No |
| row_30 | NAA38 | 23539 | 0.027206779 | -0.3115441 | No |
| row_31 | PDS5B | 23596 | 0.026869275 | -0.3114587 | No |
| row_32 | RBBP7 | 24027 | 0.02419929 | -0.3182876 | No |
| row_33 | CBX5 | 24073 | 0.023909913 | -0.3181237 | No |
| row_34 | LIG1 | 25105 | 0.017882502 | -0.33614686 | No |
| row_35 | RAD51C | 25268 | 0.016989511 | -0.33839607 | No |
| row_36 | RAD1 | 25447 | 0.01604706 | -0.3409751 | No |
| row_37 | PLK1 | 26106 | 0.012454868 | -0.35243493 | No |
| row_38 | POLD3 | 26431 | 0.010903163 | -0.3578817 | No |
| row_39 | PPM1D | 26561 | 0.010183985 | -0.3598102 | No |
| row_40 | PLK4 | 27004 | 0.008002543 | -0.36752307 | No |
| row_41 | LUC7L3 | 27168 | 0.007231516 | -0.3701915 | No |
| row_42 | EED | 27583 | 0.005095328 | -0.37751445 | No |
| row_43 | UBE2T | 27697 | 0.004527141 | -0.37938434 | No |
| row_44 | CKS1B | 28595 | 4.53E-04 | -0.3956858 | No |
| row_45 | PRDX4 | 28675 | 1.02E-04 | -0.39711893 | No |
| row_46 | PTTG1 | 31570 | -0.001228016 | -0.44972208 | No |
| row_47 | TRA2B | 32073 | -0.003422249 | -0.45871484 | No |
| row_48 | CENPM | 32264 | -0.004198687 | -0.46199915 | No |
| row_49 | RFC2 | 33058 | -0.007962223 | -0.47609982 | No |
| row_50 | MKI67 | 33500 | -0.009981571 | -0.48371318 | No |
| row_51 | STMN1 | 34077 | -0.012734887 | -0.49366957 | No |
| row_52 | SMC4 | 34181 | -0.013207485 | -0.49500078 | No |
| row_53 | HMMR | 34288 | -0.013691036 | -0.49636668 | No |
| row_54 | PAICS | 35101 | -0.017470522 | -0.5104222 | No |
| row_55 | CSE1L | 35450 | -0.019318275 | -0.51595986 | No |
| row_56 | TBRG4 | 35533 | -0.019808531 | -0.5166377 | No |
| row_57 | MYC | 35592 | -0.020162469 | -0.5168643 | No |
| row_58 | ING3 | 35595 | -0.020172477 | -0.5160716 | No |
| row_59 | LMNB1 | 35755 | -0.020957278 | -0.5181032 | No |
| row_60 | ESPL1 | 36639 | -0.025414074 | -0.5331241 | No |
| row_61 | HNRNPD | 36722 | -0.025853613 | -0.5335535 | No |
| row_62 | CENPE | 36736 | -0.025947208 | -0.5327236 | No |
| row_63 | CNOT9 | 36937 | -0.027088134 | -0.5352492 | No |
| row_64 | PPP1R8 | 37466 | -0.030117407 | -0.5436179 | No |
| row_65 | RPA1 | 38052 | -0.033522442 | -0.55288374 | No |
| row_66 | RPA2 | 38078 | -0.033644527 | -0.5519559 | No |
| row_67 | BRCA2 | 38109 | -0.033858962 | -0.55111015 | No |
| row_68 | DCTPP1 | 38328 | -0.035074025 | -0.553635 | No |
| row_69 | SRSF1 | 39373 | -0.041606437 | -0.57091963 | No |
| row_70 | BRCA1 | 39833 | -0.044701047 | -0.57743365 | No |
| row_71 | XPO1 | 39968 | -0.045505673 | -0.57800144 | No |
| row_72 | NUDT21 | 39971 | -0.045522764 | -0.5761669 | No |
| row_73 | KIF4A | 40543 | -0.0494087 | -0.58452517 | No |
| row_74 | SPC25 | 40590 | -0.0497026 | -0.5833194 | No |
| row_75 | NASP | 40707 | -0.050490122 | -0.58335495 | No |
| row_76 | EZH2 | 41017 | -0.052850146 | -0.58680487 | No |
| row_77 | NAP1L1 | 41414 | -0.055633567 | -0.5917233 | No |
| row_78 | NUP107 | 41908 | -0.059585135 | -0.5982442 | No |
| row_79 | SMC1A | 42005 | -0.060330424 | -0.59751135 | No |
| row_80 | XRCC6 | 42158 | -0.061603099 | -0.5977451 | No |
| row_81 | CDCA3 | 42208 | -0.061990485 | -0.59608895 | No |
| row_82 | SUV39H1 | 42223 | -0.062114321 | -0.5937909 | No |
| row_83 | RAN | 42277 | -0.062589169 | -0.59218293 | No |
| row_84 | ZW10 | 42604 | -0.065384448 | -0.59542704 | No |
| row_85 | CDC25B | 42626 | -0.06559597 | -0.59311324 | No |
| row_86 | UBE2S | 43132 | -0.069741867 | -0.59943503 | No |
| row_87 | POLD2 | 43155 | -0.06992171 | -0.5969617 | No |
| row_88 | ANP32E | 43486 | -0.073073544 | -0.5999626 | No |
| row_89 | GINS4 | 43514 | -0.073328733 | -0.5974402 | No |
| row_90 | CTCF | 43911 | -0.07729397 | -0.6014684 | No |
| row_91 | ATAD2 | 44003 | -0.078108832 | -0.59991395 | No |
| row_92 | RPA3 | 44137 | -0.079478249 | -0.5990674 | No |
| row_93 | DSCC1 | 44492 | -0.08332254 | -0.60208374 | No |
| row_94 | MRE11 | 44714 | -0.085497856 | -0.60259086 | Yes |
| row_95 | EXOSC8 | 44841 | -0.086832553 | -0.6013147 | Yes |
| row_96 | E2F8 | 44881 | -0.087211892 | -0.59844005 | Yes |
| row_97 | TIMELESS | 44923 | -0.087626912 | -0.59558475 | Yes |
| row_98 | MSH2 | 45058 | -0.089038812 | -0.59436345 | Yes |
| row_99 | PHF5A | 45103 | -0.089350648 | -0.5914919 | Yes |
| row_100 | KIF2C | 45544 | -0.094730772 | -0.59560406 | Yes |
| row_101 | CDK4 | 45558 | -0.094977871 | -0.5919372 | Yes |
| row_102 | MELK | 45607 | -0.095739655 | -0.58887583 | Yes |
| row_103 | TCF19 | 45731 | -0.097053438 | -0.58712506 | Yes |
| row_104 | TMPO | 45764 | -0.097471751 | -0.5837014 | Yes |
| row_105 | CCNB2 | 45878 | -0.098811537 | -0.5816964 | Yes |
| row_106 | SNRPB | 45969 | -0.100130461 | -0.57921875 | Yes |
| row_107 | SMC6 | 45977 | -0.100176975 | -0.57522905 | Yes |
| row_108 | UNG | 46046 | -0.100858964 | -0.5723212 | Yes |
| row_109 | UBR7 | 46049 | -0.100921281 | -0.56820995 | Yes |
| row_110 | DEPDC1 | 46171 | -0.102346823 | -0.5662052 | Yes |
| row_111 | TUBB | 46541 | -0.106754564 | -0.5685314 | Yes |
| row_112 | MCM2 | 46674 | -0.108500712 | -0.5664739 | Yes |
| row_113 | CHEK2 | 46710 | -0.109004416 | -0.5626309 | Yes |
| row_114 | HMGB3 | 46865 | -0.110910691 | -0.5608746 | Yes |
| row_115 | DUT | 46966 | -0.112225227 | -0.5580818 | Yes |
| row_116 | NOLC1 | 47034 | -0.113254949 | -0.55464625 | Yes |
| row_117 | TK1 | 47222 | -0.116000876 | -0.5532812 | Yes |
| row_118 | SYNCRIP | 47312 | -0.117304675 | -0.5500795 | Yes |
| row_119 | PCNA | 47467 | -0.11965283 | -0.5479639 | Yes |
| row_120 | ORC2 | 47493 | -0.120015927 | -0.5434864 | Yes |
| row_121 | GINS1 | 47720 | -0.123333894 | -0.5425295 | Yes |
| row_122 | MCM4 | 47737 | -0.123606369 | -0.5377407 | Yes |
| row_123 | MAD2L1 | 47840 | -0.125117183 | -0.53445446 | Yes |
| row_124 | PA2G4 | 48105 | -0.128896028 | -0.53396034 | Yes |
| row_125 | ORC6 | 48188 | -0.129926041 | -0.5301126 | Yes |
| row_126 | WDR90 | 48284 | -0.131326646 | -0.52644384 | Yes |
| row_127 | TACC3 | 48566 | -0.135935709 | -0.5259697 | Yes |
| row_128 | CDKN1B | 48577 | -0.136110321 | -0.5205578 | Yes |
| row_129 | ASF1A | 48615 | -0.136738792 | -0.51561135 | Yes |
| row_130 | MCM3 | 48781 | -0.139532119 | -0.51287895 | Yes |
| row_131 | BUB1B | 48855 | -0.14055948 | -0.5084304 | Yes |
| row_132 | SSRP1 | 48887 | -0.14099367 | -0.50319993 | Yes |
| row_133 | ASF1B | 48888 | -0.141021892 | -0.49740425 | Yes |
| row_134 | DNMT1 | 48982 | -0.142564699 | -0.49323723 | Yes |
| row_135 | CTPS1 | 49038 | -0.143607199 | -0.48833597 | Yes |
| row_136 | NCAPD2 | 49061 | -0.144109949 | -0.48281366 | Yes |
| row_137 | DCLRE1B | 49062 | -0.14411068 | -0.47689104 | Yes |
| row_138 | TIPIN | 49176 | -0.146587834 | -0.47292253 | Yes |
| row_139 | SLBP | 49251 | -0.147995338 | -0.46818662 | Yes |
| row_140 | RFC3 | 49261 | -0.148157433 | -0.46226144 | Yes |
| row_141 | SRSF2 | 49360 | -0.150000259 | -0.45787978 | Yes |
| row_142 | PRPS1 | 49411 | -0.151118413 | -0.45257887 | Yes |
| row_143 | RACGAP1 | 49440 | -0.151635572 | -0.44685644 | Yes |
| row_144 | POLE4 | 49540 | -0.153427869 | -0.44235212 | Yes |
| row_145 | TUBG1 | 49556 | -0.153816298 | -0.43630353 | Yes |
| row_146 | MYBL2 | 49638 | -0.155518502 | -0.4313858 | Yes |
| row_147 | CDCA8 | 49643 | -0.155571342 | -0.42506495 | Yes |
| row_148 | DDX39A | 49711 | -0.156941116 | -0.41983405 | Yes |
| row_149 | CDKN3 | 49756 | -0.157749042 | -0.41415146 | Yes |
| row_150 | GSPT1 | 50096 | -0.164932549 | -0.4135409 | Yes |
| row_151 | GINS3 | 50215 | -0.167760134 | -0.40879324 | Yes |
| row_152 | NOP56 | 50305 | -0.170424014 | -0.40340847 | Yes |
| row_153 | PMS2 | 50369 | -0.17224507 | -0.3974758 | Yes |
| row_154 | DCK | 50459 | -0.174529344 | -0.39192232 | Yes |
| row_155 | AURKB | 50602 | -0.177775592 | -0.38719973 | Yes |
| row_156 | NBN | 50615 | -0.178081885 | -0.3800993 | Yes |
| row_157 | PSMC3IP | 50721 | -0.18128182 | -0.3745594 | Yes |
| row_158 | PRIM2 | 50992 | -0.188472494 | -0.371726 | Yes |
| row_159 | MCM5 | 51073 | -0.190538988 | -0.3653508 | Yes |
| row_160 | CDC20 | 51133 | -0.192432612 | -0.35851574 | Yes |
| row_161 | IPO7 | 51209 | -0.194831565 | -0.35187316 | Yes |
| row_162 | HMGA1 | 51245 | -0.196102068 | -0.34445062 | Yes |
| row_163 | RFC1 | 51316 | -0.198112994 | -0.3375822 | Yes |
| row_164 | DIAPH3 | 51346 | -0.198764205 | -0.3299411 | Yes |
| row_165 | JPT1 | 51352 | -0.198985904 | -0.3218542 | Yes |
| row_166 | KPNA2 | 51383 | -0.199786276 | -0.31418926 | Yes |
| row_167 | BIRC5 | 51537 | -0.204349563 | -0.30857465 | Yes |
| row_168 | BRMS1L | 51833 | -0.214690983 | -0.3051186 | Yes |
| row_169 | MCM6 | 51859 | -0.216100544 | -0.2966922 | Yes |
| row_170 | USP1 | 52115 | -0.225731149 | -0.29205465 | Yes |
| row_171 | RANBP1 | 52159 | -0.227170914 | -0.2835008 | Yes |
| row_172 | RRM2 | 52244 | -0.230985731 | -0.27553612 | Yes |
| row_173 | RAD51AP1 | 52311 | -0.233875692 | -0.2671252 | Yes |
| row_174 | BARD1 | 52353 | -0.236083642 | -0.25816864 | Yes |
| row_175 | EIF2S1 | 52374 | -0.237062782 | -0.24878979 | Yes |
| row_176 | ILF3 | 52442 | -0.23985064 | -0.24015148 | Yes |
| row_177 | KIF18B | 52461 | -0.240694955 | -0.23058696 | Yes |
| row_178 | DEK | 52643 | -0.249379933 | -0.22363114 | Yes |
| row_179 | PNN | 52694 | -0.252238929 | -0.2141744 | Yes |
| row_180 | DLGAP5 | 52705 | -0.252783507 | -0.20396753 | Yes |
| row_181 | RNASEH2A | 52766 | -0.255186379 | -0.1945716 | Yes |
| row_182 | POP7 | 52846 | -0.259660691 | -0.18533747 | Yes |
| row_183 | NME1 | 52992 | -0.267384678 | -0.17698671 | Yes |
| row_184 | SHMT1 | 53060 | -0.271767437 | -0.1670367 | Yes |
| row_185 | HELLS | 53143 | -0.276774406 | -0.15715382 | Yes |
| row_186 | NUP153 | 53160 | -0.277757436 | -0.14602974 | Yes |
| row_187 | SPC24 | 53396 | -0.295309007 | -0.13816881 | Yes |
| row_188 | DONSON | 53574 | -0.311588138 | -0.12858361 | Yes |
| row_189 | CDC25A | 53694 | -0.32306096 | -0.117471635 | Yes |
| row_190 | TRIP13 | 53741 | -0.327013791 | -0.10486905 | Yes |
| row_191 | CCNE1 | 53758 | -0.328384548 | -0.09166431 | Yes |
| row_192 | MLH1 | 53803 | -0.333040982 | -0.078777626 | Yes |
| row_193 | MCM7 | 54400 | -0.422869325 | -0.07224233 | Yes |
| row_194 | CDKN2A | 54561 | -0.459026843 | -0.05628843 | Yes |
| row_195 | SPAG5 | 54698 | -0.507282257 | -0.037914686 | Yes |
| row_196 | MTHFD2 | 54796 | -0.555831432 | -0.016836118 | Yes |
| row_197 | TOP2A | 54829 | -0.570367217 | 0.006022452 | Yes |

EAC MYC Targets V1

| NAME | GENE SYMBOL | RANK IN GENE LIST | RANK METRIC SCORE | RUNNING ES | CORE ENRICHMENT |
| --- | --- | --- | --- | --- | --- |
| row_0 | RUVBL2 | 12149 | 0.130283609 | -0.21459745 | No |
| row_1 | AP3S1 | 12699 | 0.123293087 | -0.21849675 | No |
| row_2 | SNRPA1 | 13087 | 0.118579827 | -0.21968149 | No |
| row_3 | PSMA7 | 13169 | 0.117556132 | -0.21534958 | No |
| row_4 | GLO1 | 13792 | 0.110392146 | -0.22121412 | No |
| row_5 | PSMD8 | 14376 | 0.103824526 | -0.22669345 | No |
| row_6 | NCBP2 | 14590 | 0.101566091 | -0.22555277 | No |
| row_7 | U2AF1 | 15307 | 0.09391766 | -0.23394108 | No |
| row_8 | LSM7 | 15836 | 0.088734157 | -0.23916502 | No |
| row_9 | PSMC4 | 16198 | 0.085080296 | -0.2415311 | No |
| row_10 | FAM120A | 16304 | 0.084133767 | -0.23928644 | No |
| row_11 | PRPF31 | 16315 | 0.084002092 | -0.2353199 | No |
| row_12 | CLNS1A | 16833 | 0.07894484 | -0.24082716 | No |
| row_13 | COPS5 | 17365 | 0.073906496 | -0.24683794 | No |
| row_14 | SNRPD2 | 17506 | 0.072596528 | -0.24579981 | No |
| row_15 | EIF4A1 | 18017 | 0.06806507 | -0.25171703 | No |
| row_16 | RSL1D1 | 18031 | 0.067977257 | -0.24859646 | No |
| row_17 | NCBP1 | 18971 | 0.060161874 | -0.26270893 | No |
| row_18 | EIF1AX | 19691 | 0.054748025 | -0.27308625 | No |
| row_19 | UBA2 | 20682 | 0.047003575 | -0.2887764 | No |
| row_20 | TXNL4A | 20962 | 0.044942554 | -0.29163286 | No |
| row_21 | HNRNPA3 | 21253 | 0.042841911 | -0.2947932 | No |
| row_22 | EIF4H | 21480 | 0.040969491 | -0.2968816 | No |
| row_23 | NDUFAB1 | 21658 | 0.03963301 | -0.29814452 | No |
| row_24 | BUB3 | 21723 | 0.038986128 | -0.29738355 | No |
| row_25 | RPL18 | 22017 | 0.037006143 | -0.30088666 | No |
| row_26 | PPIA | 22274 | 0.035241634 | -0.30380374 | No |
| row_27 | PRDX3 | 22340 | 0.034891404 | -0.3032632 | No |
| row_28 | RNPS1 | 22616 | 0.033076424 | -0.30663288 | No |
| row_29 | SRPK1 | 23255 | 0.028921144 | -0.31681198 | No |
| row_30 | PSMD7 | 23842 | 0.025360767 | -0.32622087 | No |
| row_31 | RAD23B | 24413 | 0.021764193 | -0.33551627 | No |
| row_32 | CCT3 | 24739 | 0.019954959 | -0.34044364 | No |
| row_33 | UBE2L3 | 25325 | 0.016739309 | -0.3502601 | No |
| row_34 | CANX | 25359 | 0.016544968 | -0.35004342 | No |
| row_35 | HDGF | 26234 | 0.011853596 | -0.36535904 | No |
| row_36 | NHP2 | 26380 | 0.011163344 | -0.36744577 | No |
| row_37 | SF3B3 | 26753 | 0.009203283 | -0.3737592 | No |
| row_38 | PSMB2 | 26878 | 0.008596419 | -0.37559065 | No |
| row_39 | SNRPG | 28226 | 0.002067442 | -0.39999506 | No |
| row_40 | PRDX4 | 28675 | 1.02E-04 | -0.4081407 | No |
| row_41 | RPS5 | 31469 | -7.02E-04 | -0.45892015 | No |
| row_42 | TRA2B | 32073 | -0.003422249 | -0.46972176 | No |
| row_43 | SNRPA | 32751 | -0.006538487 | -0.4817158 | No |
| row_44 | TOMM70 | 33336 | -0.009217663 | -0.4918855 | No |
| row_45 | CUL1 | 33448 | -0.009667356 | -0.49342754 | No |
| row_46 | DDX18 | 33688 | -0.010865908 | -0.49723917 | No |
| row_47 | CCT5 | 33917 | -0.011920977 | -0.5007985 | No |
| row_48 | IFRD1 | 34529 | -0.014767092 | -0.5111854 | No |
| row_49 | ABCE1 | 34540 | -0.014817954 | -0.51063555 | No |
| row_50 | PSMA2 | 34634 | -0.015246223 | -0.5115746 | No |
| row_51 | PSMD1 | 34751 | -0.015728042 | -0.5129083 | No |
| row_52 | HNRNPU | 34981 | -0.016858604 | -0.516242 | No |
| row_53 | MYC | 35592 | -0.020162469 | -0.52634424 | No |
| row_54 | ERH | 35804 | -0.021220697 | -0.52913505 | No |
| row_55 | VDAC3 | 36007 | -0.022294935 | -0.5317091 | No |
| row_56 | MRPL23 | 36142 | -0.0229009 | -0.533016 | No |
| row_57 | POLE3 | 36528 | -0.024872879 | -0.53879213 | No |
| row_58 | HNRNPD | 36722 | -0.025853613 | -0.54102665 | No |
| row_59 | HNRNPA2B1 | 36898 | -0.026893459 | -0.5428824 | No |
| row_60 | RPS2 | 37296 | -0.02903505 | -0.54867125 | No |
| row_61 | HDDC2 | 37790 | -0.0319589 | -0.5560623 | No |
| row_62 | CNBP | 37834 | -0.032175045 | -0.55525565 | No |
| row_63 | EIF3J | 38100 | -0.033818785 | -0.5584067 | No |
| row_64 | SERBP1 | 38380 | -0.035409529 | -0.56173396 | No |
| row_65 | FBL | 38461 | -0.035832409 | -0.56141984 | No |
| row_66 | MRPS18B | 38704 | -0.037254389 | -0.56398284 | No |
| row_67 | SSB | 38923 | -0.03851252 | -0.566047 | No |
| row_68 | SNRPB2 | 39013 | -0.03902974 | -0.56573874 | No |
| row_69 | SRSF1 | 39373 | -0.041606437 | -0.5702154 | No |
| row_70 | HSPE1 | 39654 | -0.043405782 | -0.57316595 | No |
| row_71 | TFDP1 | 39736 | -0.043988463 | -0.5724672 | No |
| row_72 | XPO1 | 39968 | -0.045505673 | -0.5744226 | No |
| row_73 | DDX21 | 40312 | -0.047749136 | -0.5783048 | No |
| row_74 | PTGES3 | 40331 | -0.047875457 | -0.57626796 | No |
| row_75 | TRIM28 | 40354 | -0.048063431 | -0.57429457 | No |
| row_76 | RPL22 | 40449 | -0.048718087 | -0.5735988 | No |
| row_77 | VBP1 | 40515 | -0.049176231 | -0.57235277 | No |
| row_78 | PSMD3 | 40643 | -0.049994305 | -0.57219434 | No |
| row_79 | HNRNPR | 40685 | -0.050283048 | -0.57045704 | No |
| row_80 | HPRT1 | 40876 | -0.051735029 | -0.5713588 | No |
| row_81 | STARD7 | 41073 | -0.053268168 | -0.57229406 | No |
| row_82 | SLC25A3 | 41243 | -0.054382294 | -0.57268304 | No |
| row_83 | EIF4E | 41302 | -0.054882597 | -0.5710279 | No |
| row_84 | NAP1L1 | 41414 | -0.055633567 | -0.57029986 | No |
| row_85 | PRPS2 | 41697 | -0.057741497 | -0.5725788 | No |
| row_86 | PHB | 42071 | -0.060987771 | -0.576353 | No |
| row_87 | XRCC6 | 42158 | -0.061603099 | -0.57487535 | No |
| row_88 | RAN | 42277 | -0.062589169 | -0.5739312 | No |
| row_89 | SSBP1 | 42564 | -0.06506189 | -0.5759214 | No |
| row_90 | ILF2 | 42645 | -0.065740995 | -0.57413024 | No |
| row_91 | POLD2 | 43155 | -0.06992171 | -0.5799375 | Yes |
| row_92 | RPL34 | 43254 | -0.070809007 | -0.5782236 | Yes |
| row_93 | NOP16 | 43328 | -0.07159961 | -0.5760157 | Yes |
| row_94 | CCNA2 | 43473 | -0.072972454 | -0.57503176 | Yes |
| row_95 | EIF3B | 43766 | -0.075733796 | -0.5766041 | Yes |
| row_96 | TUFM | 43957 | -0.077704795 | -0.5762234 | Yes |
| row_97 | HNRNPC | 44119 | -0.07931415 | -0.57523555 | Yes |
| row_98 | RFC4 | 44169 | -0.079877563 | -0.57218224 | Yes |
| row_99 | GOT2 | 44195 | -0.080081888 | -0.5686822 | Yes |
| row_100 | CCT2 | 44473 | -0.0830255 | -0.5696215 | Yes |
| row_101 | PSMD14 | 44736 | -0.085725121 | -0.5701546 | Yes |
| row_102 | PCBP1 | 44935 | -0.087748408 | -0.56942344 | Yes |
| row_103 | SRSF7 | 44948 | -0.087910898 | -0.5653002 | Yes |
| row_104 | SNRPD3 | 45253 | -0.091124237 | -0.5663308 | Yes |
| row_105 | LSM2 | 45311 | -0.091983482 | -0.5628252 | Yes |
| row_106 | CDK4 | 45558 | -0.094977871 | -0.56261027 | Yes |
| row_107 | PSMA6 | 45627 | -0.095862858 | -0.5591132 | Yes |
| row_108 | EIF2S2 | 45677 | -0.096361428 | -0.5552458 | Yes |
| row_109 | SRM | 45837 | -0.098303705 | -0.5532838 | Yes |
| row_110 | MRPL9 | 46045 | -0.100854106 | -0.5520691 | Yes |
| row_111 | CBX3 | 46165 | -0.102269173 | -0.5491835 | Yes |
| row_112 | PSMA4 | 46193 | -0.102703519 | -0.5446027 | Yes |
| row_113 | G3BP1 | 46259 | -0.10367173 | -0.5406654 | Yes |
| row_114 | RPLP0 | 46538 | -0.106721357 | -0.54045266 | Yes |
| row_115 | MCM2 | 46674 | -0.108500712 | -0.53755045 | Yes |
| row_116 | RACK1 | 46760 | -0.109655589 | -0.53368145 | Yes |
| row_117 | EIF3D | 46808 | -0.110174321 | -0.5290956 | Yes |
| row_118 | YWHAQ | 46870 | -0.110976458 | -0.5247247 | Yes |
| row_119 | SNRPD1 | 46912 | -0.111509435 | -0.51996374 | Yes |
| row_120 | DUT | 46966 | -0.112225227 | -0.5153857 | Yes |
| row_121 | PWP1 | 46974 | -0.112282798 | -0.5099679 | Yes |
| row_122 | NOLC1 | 47034 | -0.113254949 | -0.50544816 | Yes |
| row_123 | UBE2E1 | 47268 | -0.116639577 | -0.50392693 | Yes |
| row_124 | PABPC4 | 47290 | -0.116841823 | -0.49853873 | Yes |
| row_125 | SYNCRIP | 47312 | -0.117304675 | -0.49312767 | Yes |
| row_126 | CAD | 47334 | -0.117832638 | -0.4876905 | Yes |
| row_127 | PCNA | 47467 | -0.11965283 | -0.48418295 | Yes |
| row_128 | EIF4G2 | 47492 | -0.120000027 | -0.47869334 | Yes |
| row_129 | ORC2 | 47493 | -0.120015927 | -0.4727663 | Yes |
| row_130 | RPS3 | 47540 | -0.12065579 | -0.46764457 | Yes |
| row_131 | KPNB1 | 47690 | -0.122820042 | -0.46428984 | Yes |
| row_132 | MCM4 | 47737 | -0.123606369 | -0.4590224 | Yes |
| row_133 | SRSF3 | 47813 | -0.124788225 | -0.4542242 | Yes |
| row_134 | MAD2L1 | 47840 | -0.125117183 | -0.44851825 | Yes |
| row_135 | HNRNPA1 | 47842 | -0.125165433 | -0.4423551 | Yes |
| row_136 | YWHAE | 48011 | -0.127700821 | -0.43910503 | Yes |
| row_137 | CCT7 | 48034 | -0.12795271 | -0.4331863 | Yes |
| row_138 | AIMP2 | 48068 | -0.128521547 | -0.4274396 | Yes |
| row_139 | PA2G4 | 48105 | -0.128896028 | -0.42172897 | Yes |
| row_140 | RPS6 | 48143 | -0.129409149 | -0.4160112 | Yes |
| row_141 | PSMA1 | 48168 | -0.129652992 | -0.41004488 | Yes |
| row_142 | ACP1 | 48271 | -0.131132632 | -0.40542457 | Yes |
| row_143 | VDAC1 | 48326 | -0.132047713 | -0.39988577 | Yes |
| row_144 | CSTF2 | 48410 | -0.133388594 | -0.39480838 | Yes |
| row_145 | CCT4 | 48650 | -0.137550533 | -0.39236364 | Yes |
| row_146 | TARDBP | 48758 | -0.139177248 | -0.387437 | Yes |
| row_147 | TYMS | 48774 | -0.139460176 | -0.3808226 | Yes |
| row_148 | SF3A1 | 48983 | -0.142609805 | -0.37756398 | Yes |
| row_149 | COX5A | 49017 | -0.143388584 | -0.37108305 | Yes |
| row_150 | CTPS1 | 49038 | -0.143607199 | -0.36435482 | Yes |
| row_151 | SET | 49089 | -0.144568607 | -0.3581249 | Yes |
| row_152 | ODC1 | 49263 | -0.148200676 | -0.35395342 | Yes |
| row_153 | SRSF2 | 49360 | -0.150000259 | -0.34829217 | Yes |
| row_154 | PSMC6 | 49503 | -0.152736887 | -0.34333265 | Yes |
| row_155 | C1QBP | 49560 | -0.15390487 | -0.33675084 | Yes |
| row_156 | PHB2 | 49753 | -0.157712385 | -0.33245528 | Yes |
| row_157 | RPS10 | 49987 | -0.162609324 | -0.32866383 | Yes |
| row_158 | CYC1 | 50002 | -0.162861243 | -0.32087556 | Yes |
| row_159 | GSPT1 | 50096 | -0.164932549 | -0.31442228 | Yes |
| row_160 | PPM1G | 50144 | -0.166230619 | -0.307068 | Yes |
| row_161 | RPL6 | 50244 | -0.168690071 | -0.30053833 | Yes |
| row_162 | NOP56 | 50305 | -0.170424014 | -0.29321346 | Yes |
| row_163 | DHX15 | 50423 | -0.173530653 | -0.28677222 | Yes |
| row_164 | HSPD1 | 50595 | -0.177625105 | -0.2811112 | Yes |
| row_165 | MCM5 | 51073 | -0.190538988 | -0.2803796 | Yes |
| row_166 | EXOSC7 | 51105 | -0.191593975 | -0.27148166 | Yes |
| row_167 | PGK1 | 51117 | -0.19191356 | -0.26220405 | Yes |
| row_168 | CDC20 | 51133 | -0.192432612 | -0.2529736 | Yes |
| row_169 | TCP1 | 51165 | -0.193664581 | -0.24397339 | Yes |
| row_170 | XPOT | 51205 | -0.194719911 | -0.23506661 | Yes |
| row_171 | KPNA2 | 51383 | -0.199786276 | -0.22842032 | Yes |
| row_172 | APEX1 | 51447 | -0.20185855 | -0.21959764 | Yes |
| row_173 | HDAC2 | 51450 | -0.20190993 | -0.20966262 | Yes |
| row_174 | PABPC1 | 51494 | -0.202988744 | -0.20042025 | Yes |
| row_175 | RPL14 | 51688 | -0.209763259 | -0.19357233 | Yes |
| row_176 | SMARCC1 | 51734 | -0.211257264 | -0.18395801 | Yes |
| row_177 | MCM6 | 51859 | -0.216100544 | -0.17554179 | Yes |
| row_178 | RRM1 | 51972 | -0.220480055 | -0.16669095 | Yes |
| row_179 | LDHA | 52097 | -0.224973291 | -0.15783653 | Yes |
| row_180 | USP1 | 52115 | -0.225731149 | -0.146998 | Yes |
| row_181 | RANBP1 | 52159 | -0.227170914 | -0.13656138 | Yes |
| row_182 | ETF1 | 52176 | -0.227666453 | -0.12560908 | Yes |
| row_183 | EIF2S1 | 52374 | -0.237062782 | -0.11748574 | Yes |
| row_184 | RRP9 | 52417 | -0.238809407 | -0.10645616 | Yes |
| row_185 | NPM1 | 52592 | -0.246517494 | -0.09744745 | Yes |
| row_186 | PSMB3 | 52622 | -0.248231888 | -0.085716024 | Yes |
| row_187 | CDK2 | 52626 | -0.248565316 | -0.07349511 | Yes |
| row_188 | DEK | 52643 | -0.249379933 | -0.061470475 | Yes |
| row_189 | EEF1B2 | 52656 | -0.250150561 | -0.04933501 | Yes |
| row_190 | HSP90AB1 | 52983 | -0.266532898 | -0.042103224 | Yes |
| row_191 | NME1 | 52992 | -0.267384678 | -0.029043872 | Yes |
| row_192 | IMPDH2 | 53541 | -0.307878673 | -0.02380914 | Yes |
| row_193 | GNL3 | 53689 | -0.322573781 | -0.010553136 | Yes |
| row_194 | CDC45 | 53792 | -0.331991881 | 0.00398669 | Yes |
| row_195 | MCM7 | 54400 | -0.422869325 | 0.01382687 | Yes |

EAC MYC Targets V2

| NAME | GENE SYMBOL | RANK IN GENE LIST | RANK METRIC SCORE | RUNNING ES | CORE ENRICHMENT |
| --- | --- | --- | --- | --- | --- |
| row_0 | GRWD1 | 7661 | 0.204400495 | -0.11211673 | No |
| row_1 | EXOSC5 | 10199 | 0.158388913 | -0.13730241 | No |
| row_2 | MRTO4 | 13302 | 0.116066292 | -0.17831431 | No |
| row_3 | SORD | 13701 | 0.111362942 | -0.17087378 | No |
| row_4 | MAP3K6 | 20368 | 0.049649436 | -0.2853098 | No |
| row_5 | SUPV3L1 | 23777 | 0.025734741 | -0.34376904 | No |
| row_6 | IMP4 | 26047 | 0.012736653 | -0.38326943 | No |
| row_7 | PLK1 | 26106 | 0.012454868 | -0.38268203 | No |
| row_8 | PLK4 | 27004 | 0.008002543 | -0.39790693 | No |
| row_9 | DDX18 | 33688 | -0.010865908 | -0.51775813 | No |
| row_10 | NDUFAF4 | 34326 | -0.013870062 | -0.52749205 | No |
| row_11 | TBRG4 | 35533 | -0.019808531 | -0.5467701 | No |
| row_12 | MYC | 35592 | -0.020162469 | -0.5451678 | No |
| row_13 | DCTPP1 | 38328 | -0.035074025 | -0.59018385 | No |
| row_14 | HSPE1 | 39654 | -0.043405782 | -0.6085144 | No |
| row_15 | RABEPK | 40086 | -0.046298537 | -0.6102399 | No |
| row_16 | TCOF1 | 40407 | -0.048394222 | -0.60967505 | No |
| row_17 | DUSP2 | 41408 | -0.055600204 | -0.62050194 | No |
| row_18 | PUS1 | 41478 | -0.056182668 | -0.61435646 | No |
| row_19 | PHB | 42071 | -0.060987771 | -0.6170696 | No |
| row_20 | SLC29A2 | 42735 | -0.066534735 | -0.6203408 | No |
| row_21 | NOP16 | 43328 | -0.07159961 | -0.6216567 | No |
| row_22 | RRP12 | 43382 | -0.072198689 | -0.613112 | No |
| row_23 | NIP7 | 44211 | -0.080190137 | -0.6175796 | No |
| row_24 | CDK4 | 45558 | -0.094977871 | -0.6295007 | Yes |
| row_25 | SRM | 45837 | -0.098303705 | -0.62160194 | Yes |
| row_26 | UNG | 46046 | -0.100858964 | -0.61209637 | Yes |
| row_27 | CBX3 | 46165 | -0.102269173 | -0.60077184 | Yes |
| row_28 | LAS1L | 46854 | -0.110817857 | -0.59866595 | Yes |
| row_29 | NOLC1 | 47034 | -0.113254949 | -0.5870019 | Yes |
| row_30 | PES1 | 47733 | -0.123547345 | -0.5834014 | Yes |
| row_31 | MCM4 | 47737 | -0.123606369 | -0.56718034 | Yes |
| row_32 | UTP20 | 47802 | -0.124541387 | -0.5519432 | Yes |
| row_33 | AIMP2 | 48068 | -0.128521547 | -0.5398297 | Yes |
| row_34 | PA2G4 | 48105 | -0.128896028 | -0.52351105 | Yes |
| row_35 | WDR74 | 48343 | -0.132292569 | -0.51039284 | Yes |
| row_36 | PPRC1 | 48451 | -0.134051546 | -0.49468383 | Yes |
| row_37 | MYBBP1A | 48569 | -0.135962546 | -0.4789047 | Yes |
| row_38 | MPHOSPH10 | 48769 | -0.139307901 | -0.46417317 | Yes |
| row_39 | PRMT3 | 49141 | -0.145876974 | -0.4516981 | Yes |
| row_40 | TFB2M | 50179 | -0.166953638 | -0.4485343 | Yes |
| row_41 | NOP56 | 50305 | -0.170424014 | -0.42836273 | Yes |
| row_42 | HSPD1 | 50595 | -0.177625105 | -0.4102192 | Yes |
| row_43 | NOP2 | 50622 | -0.178274885 | -0.38721725 | Yes |
| row_44 | RCL1 | 50809 | -0.183262751 | -0.36646217 | Yes |
| row_45 | MCM5 | 51073 | -0.190538988 | -0.34614643 | Yes |
| row_46 | NOC4L | 51652 | -0.208385661 | -0.32919732 | Yes |
| row_47 | PPAN | 52119 | -0.225778654 | -0.30792552 | Yes |
| row_48 | RRP9 | 52417 | -0.238809407 | -0.28187093 | Yes |
| row_49 | WDR43 | 52458 | -0.240587026 | -0.25091827 | Yes |
| row_50 | IPO4 | 52475 | -0.241485372 | -0.21941179 | Yes |
| row_51 | NPM1 | 52592 | -0.246517494 | -0.18905751 | Yes |
| row_52 | BYSL | 52771 | -0.255545586 | -0.15863964 | Yes |
| row_53 | TMEM97 | 53296 | -0.2870332 | -0.1303549 | Yes |
| row_54 | FARSA | 53434 | -0.298428297 | -0.09354651 | Yes |
| row_55 | SLC19A1 | 53482 | -0.3029024 | -0.054515716 | Yes |
| row_56 | HK2 | 53688 | -0.322448611 | -0.01577859 | Yes |
| row_57 | GNL3 | 53689 | -0.322573781 | 0.026695322 | Yes |

EAC Inflammatory Response

| NAME | GENE SYMBOL | RANK IN GENE LIST | RANK METRIC SCORE | RUNNING ES | CORE ENRICHMENT |
| --- | --- | --- | --- | --- | --- |
| row_0 | CCL24 | 2 | 1.469812751 | 0.032485936 | Yes |
| row_1 | MEP1A | 142 | 0.807279289 | 0.047819417 | Yes |
| row_2 | CCL2 | 150 | 0.796279073 | 0.0653112 | Yes |
| row_3 | IL1B | 242 | 0.7179178 | 0.079540744 | Yes |
| row_4 | IFITM1 | 467 | 0.610839963 | 0.08898109 | Yes |
| row_5 | OLR1 | 478 | 0.605533779 | 0.1021977 | Yes |
| row_6 | TIMP1 | 513 | 0.595271826 | 0.114750564 | Yes |
| row_7 | C3AR1 | 610 | 0.570574939 | 0.1256289 | Yes |
| row_8 | SELE | 631 | 0.565717936 | 0.13778256 | Yes |
| row_9 | CCL5 | 663 | 0.560400188 | 0.14961842 | Yes |
| row_10 | OSM | 731 | 0.545712531 | 0.16047427 | Yes |
| row_11 | CMKLR1 | 759 | 0.539604366 | 0.17192276 | Yes |
| row_12 | DCBLD2 | 777 | 0.53709662 | 0.1834977 | Yes |
| row_13 | CD69 | 894 | 0.518284917 | 0.19285512 | Yes |
| row_14 | RGS1 | 936 | 0.510031879 | 0.20339455 | Yes |
| row_15 | IL2RB | 937 | 0.510025561 | 0.21467979 | Yes |
| row_16 | APLNR | 1028 | 0.497174978 | 0.22404319 | Yes |
| row_17 | IRF7 | 1041 | 0.495287418 | 0.234784 | Yes |
| row_18 | SELL | 1094 | 0.487260461 | 0.24461941 | Yes |
| row_19 | IL10 | 1185 | 0.476363957 | 0.25352234 | Yes |
| row_20 | IL6 | 1199 | 0.475316435 | 0.26380306 | Yes |
| row_21 | VIP | 1242 | 0.469027489 | 0.27341697 | Yes |
| row_22 | TLR1 | 1334 | 0.458258629 | 0.28190106 | Yes |
| row_23 | IL10RA | 1363 | 0.455328822 | 0.29146662 | Yes |
| row_24 | OPRK1 | 1386 | 0.452951789 | 0.30108872 | Yes |
| row_25 | C5AR1 | 1553 | 0.438611686 | 0.3077735 | Yes |
| row_26 | BST2 | 1631 | 0.431769252 | 0.3159262 | Yes |
| row_27 | EBI3 | 1682 | 0.427901357 | 0.32448456 | Yes |
| row_28 | CD48 | 1701 | 0.426266313 | 0.333589 | Yes |
| row_29 | STAB1 | 1883 | 0.41057688 | 0.33938053 | Yes |
| row_30 | MSR1 | 1905 | 0.40905872 | 0.3480496 | Yes |
| row_31 | HAS2 | 2049 | 0.398736298 | 0.35427055 | Yes |
| row_32 | CSF3R | 2085 | 0.395569682 | 0.36238644 | Yes |
| row_33 | PDPN | 2114 | 0.394054204 | 0.37059617 | Yes |
| row_34 | CD55 | 2144 | 0.391608149 | 0.37873358 | Yes |
| row_35 | CDKN1A | 2167 | 0.390260339 | 0.38696852 | Yes |
| row_36 | ITGB8 | 2217 | 0.386590749 | 0.39463103 | Yes |
| row_37 | IL1R1 | 2252 | 0.384127051 | 0.40251192 | Yes |
| row_38 | TNFSF10 | 2371 | 0.376499295 | 0.4086957 | Yes |
| row_39 | IL15 | 2386 | 0.375227123 | 0.41674355 | Yes |
| row_40 | LCK | 2614 | 0.361372232 | 0.42060938 | Yes |
| row_41 | PTGIR | 2639 | 0.359979272 | 0.42813793 | Yes |
| row_42 | TNFSF15 | 2647 | 0.359684736 | 0.43596923 | Yes |
| row_43 | P2RX7 | 2743 | 0.354868829 | 0.44209287 | Yes |
| row_44 | EREG | 2778 | 0.353402793 | 0.44929394 | Yes |
| row_45 | LCP2 | 2851 | 0.350116283 | 0.4557309 | Yes |
| row_46 | ABCA1 | 2928 | 0.346136272 | 0.46200702 | Yes |
| row_47 | NLRP3 | 2932 | 0.345734656 | 0.46960244 | Yes |
| row_48 | PROK2 | 2960 | 0.344367325 | 0.47673094 | Yes |
| row_49 | AQP9 | 2987 | 0.342810839 | 0.4838432 | Yes |
| row_50 | HBEGF | 3044 | 0.339794487 | 0.4903429 | Yes |
| row_51 | PTPRE | 3191 | 0.332875669 | 0.49505195 | Yes |
| row_52 | SRI | 3259 | 0.32983014 | 0.501131 | Yes |
| row_53 | SLC7A2 | 3436 | 0.32243371 | 0.5050632 | Yes |
| row_54 | MARCO | 3630 | 0.31469655 | 0.5085148 | Yes |
| row_55 | TNFAIP6 | 3882 | 0.305491179 | 0.5107075 | Yes |
| row_56 | GPR132 | 4257 | 0.29241547 | 0.51037294 | Yes |
| row_57 | LPAR1 | 4407 | 0.286813855 | 0.5140082 | Yes |
| row_58 | CCL7 | 4487 | 0.284185499 | 0.51885897 | Yes |
| row_59 | PDE4B | 4557 | 0.281886548 | 0.5238408 | Yes |
| row_60 | CYBB | 4580 | 0.281148881 | 0.5296614 | Yes |
| row_61 | INHBA | 4626 | 0.279546469 | 0.53502816 | Yes |
| row_62 | SLAMF1 | 4659 | 0.278708845 | 0.5406129 | Yes |
| row_63 | LY6E | 4917 | 0.270430803 | 0.5419206 | Yes |
| row_64 | CD82 | 4955 | 0.269580126 | 0.54721236 | Yes |
| row_65 | GPR183 | 5493 | 0.254633904 | 0.54307604 | Yes |
| row_66 | CXCL10 | 5946 | 0.243371934 | 0.54023707 | Yes |
| row_67 | OSMR | 6093 | 0.23963654 | 0.54288304 | Yes |
| row_68 | BEST1 | 6157 | 0.237954915 | 0.54700196 | Yes |
| row_69 | CXCL9 | 6503 | 0.229840338 | 0.54581046 | Yes |
| row_70 | CXCL6 | 6560 | 0.228445932 | 0.54984635 | Yes |
| row_71 | LAMP3 | 6771 | 0.224077538 | 0.55098355 | Yes |
| row_72 | BTG2 | 6802 | 0.223296002 | 0.55537856 | Yes |
| row_73 | KCNA3 | 7056 | 0.217532828 | 0.5555886 | Yes |
| row_74 | MEFV | 7143 | 0.215846434 | 0.55879986 | Yes |
| row_75 | IL7R | 7209 | 0.21410577 | 0.5623547 | Yes |
| row_76 | MMP14 | 7770 | 0.201845258 | 0.55663186 | Yes |
| row_77 | CSF1 | 7944 | 0.198425531 | 0.5578747 | Yes |
| row_78 | CD14 | 7991 | 0.19733873 | 0.5614042 | Yes |
| row_79 | ADRM1 | 8343 | 0.19077538 | 0.55923915 | Yes |
| row_80 | CHST2 | 8392 | 0.189763218 | 0.5625647 | Yes |
| row_81 | PIK3R5 | 8570 | 0.186623022 | 0.5634736 | Yes |
| row_82 | IL18RAP | 8678 | 0.184662446 | 0.56561273 | Yes |
| row_83 | CXCR6 | 9126 | 0.176307127 | 0.5613808 | No |
| row_84 | RTP4 | 9696 | 0.166970938 | 0.55472255 | No |
| row_85 | EMP3 | 9946 | 0.162589565 | 0.5537897 | No |
| row_86 | LTA | 10020 | 0.161453858 | 0.5560339 | No |
| row_87 | SLC31A2 | 10216 | 0.158081412 | 0.5559838 | No |
| row_88 | NMUR1 | 10386 | 0.155413687 | 0.5563477 | No |
| row_89 | CXCL11 | 10429 | 0.154840037 | 0.5590097 | No |
| row_90 | EIF2AK2 | 10462 | 0.154262349 | 0.5618408 | No |
| row_91 | TACR1 | 11230 | 0.142918378 | 0.55104774 | No |
| row_92 | PLAUR | 11629 | 0.137306258 | 0.54684436 | No |
| row_93 | ACVR2A | 12250 | 0.128977194 | 0.5384175 | No |
| row_94 | TNFRSF9 | 12409 | 0.126995012 | 0.5383528 | No |
| row_95 | ABI1 | 12572 | 0.12485452 | 0.53816783 | No |
| row_96 | IL4R | 12604 | 0.124400839 | 0.5403564 | No |
| row_97 | ADGRE1 | 12770 | 0.1224287 | 0.54006326 | No |
| row_98 | ITGB3 | 13073 | 0.118772902 | 0.5371965 | No |
| row_99 | GP1BA | 13264 | 0.116425768 | 0.5363156 | No |
| row_100 | SPHK1 | 13499 | 0.113710046 | 0.53457415 | No |
| row_101 | SCN1B | 13616 | 0.112311445 | 0.53494865 | No |
| row_102 | IL15RA | 13718 | 0.11122372 | 0.535572 | No |
| row_103 | FPR1 | 14066 | 0.10724251 | 0.53163135 | No |
| row_104 | CCRL2 | 14127 | 0.106523924 | 0.53289676 | No |
| row_105 | MXD1 | 14373 | 0.103860758 | 0.53073716 | No |
| row_106 | CALCRL | 14441 | 0.103285439 | 0.5318035 | No |
| row_107 | PSEN1 | 14501 | 0.102682814 | 0.533002 | No |
| row_108 | PVR | 14700 | 0.100480728 | 0.5316228 | No |
| row_109 | SGMS2 | 15146 | 0.095811106 | 0.52564615 | No |
| row_110 | SCARF1 | 15760 | 0.089473426 | 0.5164725 | No |
| row_111 | CCR7 | 15830 | 0.08879187 | 0.5171818 | No |
| row_112 | IL12B | 16057 | 0.086545408 | 0.5149848 | No |
| row_113 | TLR3 | 16839 | 0.078894094 | 0.5025204 | No |
| row_114 | KLF6 | 16923 | 0.078177005 | 0.50274 | No |
| row_115 | AXL | 17271 | 0.074765392 | 0.49808076 | No |
| row_116 | LIF | 17301 | 0.074515551 | 0.49920192 | No |
| row_117 | TACR3 | 17335 | 0.07417424 | 0.5002427 | No |
| row_118 | KCNMB2 | 18450 | 0.064452298 | 0.48139995 | No |
| row_119 | NPFFR2 | 18554 | 0.063606642 | 0.4809333 | No |
| row_120 | IL18R1 | 19391 | 0.056805067 | 0.46697944 | No |
| row_121 | HRH1 | 19396 | 0.056776587 | 0.46816295 | No |
| row_122 | RHOG | 19517 | 0.05593621 | 0.46721727 | No |
| row_123 | CD70 | 20088 | 0.051805045 | 0.45799255 | No |
| row_124 | TAPBP | 20274 | 0.050276879 | 0.455739 | No |
| row_125 | AHR | 20601 | 0.047654886 | 0.450862 | No |
| row_126 | NOD2 | 21064 | 0.044228397 | 0.44343466 | No |
| row_127 | PCDH7 | 21366 | 0.041916408 | 0.4388855 | No |
| row_128 | GABBR1 | 21653 | 0.03966558 | 0.4345595 | No |
| row_129 | SELENOS | 22364 | 0.034741931 | 0.42240998 | No |
| row_130 | P2RY2 | 22965 | 0.03081244 | 0.41217494 | No |
| row_131 | ROS1 | 23714 | 0.026092865 | 0.39914262 | No |
| row_132 | P2RX4 | 24880 | 0.019133192 | 0.37836915 | No |
| row_133 | BDKRB1 | 25069 | 0.018107519 | 0.3753492 | No |
| row_134 | NFKBIA | 25217 | 0.017331703 | 0.37305808 | No |
| row_135 | CLEC5A | 26831 | 0.008834614 | 0.34390548 | No |
| row_136 | SEMA4D | 27072 | 0.007733281 | 0.33970985 | No |
| row_137 | ATP2B1 | 27467 | 0.00564612 | 0.33266607 | No |
| row_138 | ICOSLG | 27675 | 0.004615273 | 0.32900187 | No |
| row_139 | SERPINE1 | 27795 | 0.004097749 | 0.32692736 | No |
| row_140 | NMI | 28482 | 0.001029328 | 0.31446856 | No |
| row_141 | ICAM4 | 29858 | -5.24E-16 | 0.28945082 | No |
| row_142 | IRF1 | 31541 | -0.001104974 | 0.25887176 | No |
| row_143 | ATP2A2 | 32821 | -0.006877019 | 0.23575288 | No |
| row_144 | NDP | 32939 | -0.007413711 | 0.23378815 | No |
| row_145 | ADORA2B | 32957 | -0.007470697 | 0.23364414 | No |
| row_146 | MET | 33124 | -0.008275635 | 0.23080693 | No |
| row_147 | NFKB1 | 33906 | -0.011833254 | 0.21685869 | No |
| row_148 | PTGER2 | 34319 | -0.013821761 | 0.2096683 | No |
| row_149 | LDLR | 34838 | -0.016187105 | 0.2006016 | No |
| row_150 | MYC | 35592 | -0.020162469 | 0.1873471 | No |
| row_151 | CD40 | 36919 | -0.027009262 | 0.16381854 | No |
| row_152 | CX3CL1 | 37542 | -0.030607812 | 0.15317868 | No |
| row_153 | CSF3 | 38047 | -0.033509146 | 0.14474998 | No |
| row_154 | F3 | 38469 | -0.035868704 | 0.13788368 | No |
| row_155 | RELA | 38887 | -0.03827576 | 0.13114339 | No |
| row_156 | KIF1B | 40544 | -0.04940974 | 0.10210622 | No |
| row_157 | IL1A | 40675 | -0.050241254 | 0.10085259 | No |
| row_158 | PTGER4 | 41584 | -0.056959011 | 0.085592106 | No |
| row_159 | LYN | 41756 | -0.058267415 | 0.08377008 | No |
| row_160 | GNAI3 | 42059 | -0.060843565 | 0.07962155 | No |
| row_161 | CCL22 | 42652 | -0.065825932 | 0.0703068 | No |
| row_162 | TLR2 | 42706 | -0.066240944 | 0.07080819 | No |
| row_163 | ITGA5 | 42882 | -0.067718223 | 0.0691225 | No |
| row_164 | CXCL8 | 43124 | -0.069682911 | 0.06627943 | No |
| row_165 | SLC31A1 | 43490 | -0.07310085 | 0.061255854 | No |
| row_166 | RAF1 | 43564 | -0.073722288 | 0.061558884 | No |
| row_167 | PTAFR | 43644 | -0.074458055 | 0.061769024 | No |
| row_168 | SLC7A1 | 43937 | -0.077556826 | 0.058172252 | No |
| row_169 | FFAR2 | 44131 | -0.079437621 | 0.056418374 | No |
| row_170 | ICAM1 | 44164 | -0.079841651 | 0.05760279 | No |
| row_171 | NAMPT | 44605 | -0.084525488 | 0.051467396 | No |
| row_172 | IFNGR2 | 44723 | -0.085651129 | 0.051233802 | No |
| row_173 | IFNAR1 | 45057 | -0.089035012 | 0.047145024 | No |
| row_174 | SLC1A2 | 46753 | -0.10959436 | 0.018729953 | No |
| row_175 | KCNJ2 | 46804 | -0.110117182 | 0.020256763 | No |
| row_176 | RASGRP1 | 47272 | -0.11669457 | 0.014341912 | No |
| row_177 | GCH1 | 48051 | -0.12815325 | 0.003022048 | No |
| row_178 | IRAK2 | 48485 | -0.134497195 | -0.001880267 | No |
| row_179 | ACVR1B | 48548 | -0.135598183 | -7.98E-06 | No |
| row_180 | EDN1 | 48691 | -0.13812989 | 4.65E-04 | No |
| row_181 | FZD5 | 48704 | -0.138429642 | 0.003309425 | No |
| row_182 | TNFSF9 | 49016 | -0.143380746 | 8.23E-04 | No |
| row_183 | TNFRSF1B | 49252 | -0.148041844 | -1.77E-04 | No |
| row_184 | TPBG | 49366 | -0.150064424 | 0.001087822 | No |
| row_185 | RIPK2 | 49437 | -0.151579335 | 0.003168165 | No |
| row_186 | RGS16 | 50094 | -0.164911821 | -0.005118593 | No |
| row_187 | ATP2C1 | 50701 | -0.180640191 | -0.012147594 | No |
| row_188 | CCL17 | 50876 | -0.184910521 | -0.011221988 | No |
| row_189 | CCL20 | 50923 | -0.18605423 | -0.007942151 | No |
| row_190 | IL18 | 51000 | -0.188679829 | -0.00515006 | No |
| row_191 | HIF1A | 52758 | -0.254959285 | -0.031476736 | No |
| row_192 | ADM | 52911 | -0.262654692 | -0.028430615 | No |
| row_193 | GPC3 | 53519 | -0.306043655 | -0.032703027 | No |
| row_194 | SLC11A2 | 53555 | -0.310180873 | -0.026476517 | No |
| row_195 | SLC4A4 | 53564 | -0.310633332 | -0.019748738 | No |
| row_196 | RNF144B | 53916 | -0.346607745 | -0.018465746 | No |
| row_197 | GNA15 | 54483 | -0.44076398 | -0.019011239 | No |
| row_198 | HPN | 54625 | -0.480003178 | -0.010955736 | No |
| row_199 | SLC28A2 | 55095 | -0.934232891 | 0.00118257 | No |

EAC IFNalpha Response

| NAME | GENE SYMBOL | RANK IN GENE LIST | RANK METRIC SCORE | RUNNING ES | CORE ENRICHMENT |
| --- | --- | --- | --- | --- | --- |
| row_0 | RSAD2 | 184 | 0.760538518 | 0.028640235 | Yes |
| row_1 | IFI44L | 282 | 0.696083426 | 0.056149993 | Yes |
| row_2 | ISG15 | 335 | 0.660443664 | 0.08297826 | Yes |
| row_3 | BATF2 | 437 | 0.617620826 | 0.10711592 | Yes |
| row_4 | IFITM1 | 467 | 0.610839963 | 0.13227597 | Yes |
| row_5 | C1S | 624 | 0.567534029 | 0.15330857 | Yes |
| row_6 | DDX60 | 674 | 0.558225632 | 0.1758929 | Yes |
| row_7 | IFIT2 | 788 | 0.535033584 | 0.19633973 | Yes |
| row_8 | GBP2 | 825 | 0.528492749 | 0.21790983 | Yes |
| row_9 | IFI44 | 833 | 0.527585745 | 0.23996845 | Yes |
| row_10 | IRF7 | 1041 | 0.495287418 | 0.2570368 | Yes |
| row_11 | SELL | 1094 | 0.487260461 | 0.27658248 | Yes |
| row_12 | TXNIP | 1133 | 0.482262611 | 0.29617223 | Yes |
| row_13 | MX1 | 1147 | 0.480322659 | 0.3161344 | Yes |
| row_14 | SAMD9L | 1374 | 0.454322636 | 0.33113506 | Yes |
| row_15 | TENT5A | 1490 | 0.443187058 | 0.34768328 | Yes |
| row_16 | IFIT3 | 1530 | 0.43990165 | 0.36547354 | Yes |
| row_17 | IFI27 | 1566 | 0.43740204 | 0.3832313 | Yes |
| row_18 | BST2 | 1631 | 0.431769252 | 0.40022555 | Yes |
| row_19 | CMPK2 | 1752 | 0.421488851 | 0.41577053 | Yes |
| row_20 | IL15 | 2386 | 0.375227123 | 0.42005387 | Yes |
| row_21 | TMEM140 | 2701 | 0.357272059 | 0.42937532 | Yes |
| row_22 | SAMD9 | 2911 | 0.346837491 | 0.44016483 | Yes |
| row_23 | USP18 | 2951 | 0.344791055 | 0.45395553 | Yes |
| row_24 | HLA-C | 3047 | 0.339698732 | 0.46651512 | Yes |
| row_25 | TRIM14 | 3132 | 0.335437864 | 0.4790953 | Yes |
| row_26 | OASL | 3163 | 0.334424973 | 0.49261355 | Yes |
| row_27 | HERC6 | 3385 | 0.324568659 | 0.5022487 | Yes |
| row_28 | HELZ2 | 3439 | 0.322200924 | 0.5148352 | Yes |
| row_29 | GBP4 | 3543 | 0.318227202 | 0.52634656 | Yes |
| row_30 | IFITM3 | 4013 | 0.30057618 | 0.53046906 | Yes |
| row_31 | PARP12 | 4296 | 0.290951222 | 0.53758276 | Yes |
| row_32 | STAT2 | 4373 | 0.288193703 | 0.54832155 | Yes |
| row_33 | PARP14 | 4641 | 0.279092044 | 0.5552089 | Yes |
| row_34 | IRF9 | 4732 | 0.276325196 | 0.56519437 | Yes |
| row_35 | LY6E | 4917 | 0.270430803 | 0.57322484 | Yes |
| row_36 | PARP9 | 5225 | 0.261748165 | 0.57865655 | Yes |
| row_37 | IFITM2 | 5272 | 0.260588706 | 0.5887793 | Yes |
| row_38 | TDRD7 | 5327 | 0.259143919 | 0.598696 | Yes |
| row_39 | CXCL10 | 5946 | 0.243371934 | 0.59770703 | Yes |
| row_40 | LAMP3 | 6771 | 0.224077538 | 0.5921657 | Yes |
| row_41 | OAS1 | 7165 | 0.215295881 | 0.5940822 | Yes |
| row_42 | ISG20 | 7709 | 0.203341648 | 0.5927719 | Yes |
| row_43 | PSMB8 | 7828 | 0.200688228 | 0.5990682 | Yes |
| row_44 | CSF1 | 7944 | 0.198425531 | 0.60532385 | Yes |
| row_45 | PLSCR1 | 8783 | 0.182607144 | 0.59778434 | Yes |
| row_46 | IFI35 | 9550 | 0.16936022 | 0.5909954 | Yes |
| row_47 | RTP4 | 9696 | 0.166970938 | 0.5953835 | Yes |
| row_48 | TAP1 | 9791 | 0.16524744 | 0.60062534 | Yes |
| row_49 | SP110 | 9897 | 0.163384989 | 0.60558903 | Yes |
| row_50 | CXCL11 | 10429 | 0.154840037 | 0.60245717 | Yes |
| row_51 | EIF2AK2 | 10462 | 0.154262349 | 0.608363 | Yes |
| row_52 | DHX58 | 10493 | 0.153662786 | 0.6142799 | Yes |
| row_53 | OGFR | 10948 | 0.147321671 | 0.6122302 | Yes |
| row_54 | LGALS3BP | 11128 | 0.144522384 | 0.6150569 | Yes |
| row_55 | EPSTI1 | 11316 | 0.14147374 | 0.61761004 | Yes |
| row_56 | B2M | 11733 | 0.136008546 | 0.6157747 | Yes |
| row_57 | CASP1 | 11871 | 0.134068295 | 0.6189245 | Yes |
| row_58 | TRIM21 | 12303 | 0.128272355 | 0.61649144 | No |
| row_59 | IL4R | 12604 | 0.124400839 | 0.61627454 | No |
| row_60 | ADAR | 12918 | 0.1206339 | 0.6156632 | No |
| row_61 | UBE2L6 | 14023 | 0.107677944 | 0.6001422 | No |
| row_62 | CCRL2 | 14127 | 0.106523924 | 0.60275114 | No |
| row_63 | MOV10 | 14593 | 0.101539113 | 0.5985764 | No |
| row_64 | PSMB9 | 15517 | 0.09160576 | 0.5856666 | No |
| row_65 | NUB1 | 15537 | 0.09142939 | 0.5891663 | No |
| row_66 | TRIM5 | 15902 | 0.088115104 | 0.5862613 | No |
| row_67 | IL7 | 16100 | 0.086073369 | 0.5863032 | No |
| row_68 | TRIM25 | 16554 | 0.081729271 | 0.5815134 | No |
| row_69 | IFIH1 | 16757 | 0.079641484 | 0.58119404 | No |
| row_70 | SLC25A28 | 18001 | 0.068193406 | 0.56148833 | No |
| row_71 | CMTR1 | 18368 | 0.065148145 | 0.55758125 | No |
| row_72 | CASP8 | 19112 | 0.059042972 | 0.5465709 | No |
| row_73 | LAP3 | 19121 | 0.058963355 | 0.54890513 | No |
| row_74 | ELF1 | 21826 | 0.038278427 | 0.5014092 | No |
| row_75 | TRAFD1 | 23962 | 0.024550932 | 0.46366924 | No |
| row_76 | LPAR6 | 24995 | 0.018536462 | 0.44570723 | No |
| row_77 | IRF2 | 27674 | 0.004621073 | 0.39726812 | No |
| row_78 | NMI | 28482 | 0.001029328 | 0.382656 | No |
| row_79 | IRF1 | 31541 | -0.001104974 | 0.3271681 | No |
| row_80 | CD47 | 31757 | -0.002068444 | 0.3233506 | No |
| row_81 | PSME1 | 32379 | -0.004784517 | 0.31227422 | No |
| row_82 | RNF31 | 36520 | -0.024840925 | 0.23813495 | No |
| row_83 | PSMA3 | 36733 | -0.025928516 | 0.23537529 | No |
| row_84 | CD74 | 40220 | -0.047136471 | 0.17405044 | No |
| row_85 | PSME2 | 41553 | -0.056752238 | 0.15224735 | No |
| row_86 | IFI30 | 43945 | -0.077624992 | 0.11209019 | No |
| row_87 | UBA7 | 44056 | -0.078672402 | 0.11340084 | No |
| row_88 | NCOA7 | 47686 | -0.122790411 | 0.052660424 | No |
| row_89 | PROCR | 47872 | -0.12564449 | 0.05458429 | No |
| row_90 | PNPT1 | 49224 | -0.147515506 | 0.03625289 | No |
| row_91 | RIPK2 | 49437 | -0.151579335 | 0.038777027 | No |
| row_92 | TRIM26 | 50596 | -0.177651256 | 0.025217826 | No |
| row_93 | CNP | 51545 | -0.204594463 | 0.0166053 | No |
| row_94 | MVB12A | 52862 | -0.260283053 | 0.00365155 | No |
| row_95 | GMPR | 55087 | -0.90514636 | 0.00132563 | No |

EAC IFNgamma Response

| NAME | GENE SYMBOL | RANK IN GENE LIST | RANK METRIC SCORE | RUNNING ES | CORE ENRICHMENT |
| --- | --- | --- | --- | --- | --- |
| row_0 | IFIT1 | 93 | 0.907944024 | 0.017674884 | Yes |
| row_1 | FGL2 | 134 | 0.814332604 | 0.034317255 | Yes |
| row_2 | CCL2 | 150 | 0.796279073 | 0.05102938 | Yes |
| row_3 | RSAD2 | 184 | 0.760538518 | 0.06665166 | Yes |
| row_4 | IFI44L | 282 | 0.696083426 | 0.07973467 | Yes |
| row_5 | GZMA | 330 | 0.663776398 | 0.093038246 | Yes |
| row_6 | CFH | 334 | 0.660999656 | 0.10708312 | Yes |
| row_7 | ISG15 | 335 | 0.660443664 | 0.121170714 | Yes |
| row_8 | BATF2 | 437 | 0.617620826 | 0.13250731 | Yes |
| row_9 | IDO1 | 438 | 0.617618442 | 0.14568143 | Yes |
| row_10 | PTGS2 | 588 | 0.575868964 | 0.15525413 | Yes |
| row_11 | XAF1 | 623 | 0.567839801 | 0.16674785 | Yes |
| row_12 | C1S | 624 | 0.567534029 | 0.17885363 | Yes |
| row_13 | CCL5 | 663 | 0.560400188 | 0.19011588 | Yes |
| row_14 | DDX60 | 674 | 0.558225632 | 0.20184118 | Yes |
| row_15 | CMKLR1 | 759 | 0.539604366 | 0.21182294 | Yes |
| row_16 | IFIT2 | 788 | 0.535033584 | 0.22272605 | Yes |
| row_17 | IFI44 | 833 | 0.527585745 | 0.2331792 | Yes |
| row_18 | C1R | 873 | 0.521520853 | 0.24359395 | Yes |
| row_19 | CD69 | 894 | 0.518284917 | 0.25428534 | Yes |
| row_20 | SERPING1 | 906 | 0.515641928 | 0.26508412 | Yes |
| row_21 | IL2RB | 937 | 0.510025561 | 0.2754174 | Yes |
| row_22 | FCGR1A | 955 | 0.505922556 | 0.2858997 | Yes |
| row_23 | DDX58 | 1038 | 0.49562332 | 0.2949797 | Yes |
| row_24 | IRF7 | 1041 | 0.495287418 | 0.30550805 | Yes |
| row_25 | TXNIP | 1133 | 0.482262611 | 0.3141393 | Yes |
| row_26 | MX1 | 1147 | 0.480322659 | 0.32414833 | Yes |
| row_27 | IL6 | 1199 | 0.475316435 | 0.33335918 | Yes |
| row_28 | SOCS1 | 1346 | 0.45701012 | 0.34045115 | Yes |
| row_29 | IL10RA | 1363 | 0.455328822 | 0.34987244 | Yes |
| row_30 | SAMD9L | 1374 | 0.454322636 | 0.35938144 | Yes |
| row_31 | IFIT3 | 1530 | 0.43990165 | 0.3659447 | Yes |
| row_32 | IFI27 | 1566 | 0.43740204 | 0.37463793 | Yes |
| row_33 | ZBP1 | 1575 | 0.43680492 | 0.38380966 | Yes |
| row_34 | BST2 | 1631 | 0.431769252 | 0.39201885 | Yes |
| row_35 | CMPK2 | 1752 | 0.421488851 | 0.39882618 | Yes |
| row_36 | OAS2 | 1839 | 0.414143026 | 0.4060954 | Yes |
| row_37 | TNFAIP3 | 1854 | 0.413203329 | 0.41465452 | Yes |
| row_38 | SSPN | 1956 | 0.405886412 | 0.42147473 | Yes |
| row_39 | METTL7B | 1998 | 0.40206489 | 0.42930502 | Yes |
| row_40 | CDKN1A | 2167 | 0.390260339 | 0.4345729 | Yes |
| row_41 | ITGB7 | 2319 | 0.379812002 | 0.43992725 | Yes |
| row_42 | TNFSF10 | 2371 | 0.376499295 | 0.44703028 | Yes |
| row_43 | IL15 | 2386 | 0.375227123 | 0.45477936 | Yes |
| row_44 | LCP2 | 2851 | 0.350116283 | 0.45380563 | Yes |
| row_45 | USP18 | 2951 | 0.344791055 | 0.45935902 | Yes |
| row_46 | IL18BP | 2999 | 0.342141479 | 0.46580195 | Yes |
| row_47 | HLA-DQA1 | 3101 | 0.33672151 | 0.47114682 | Yes |
| row_48 | TRIM14 | 3132 | 0.335437864 | 0.47775608 | Yes |
| row_49 | OASL | 3163 | 0.334424973 | 0.4843437 | Yes |
| row_50 | SRI | 3259 | 0.32983014 | 0.48965076 | Yes |
| row_51 | CD86 | 3279 | 0.328740329 | 0.49631727 | Yes |
| row_52 | HERC6 | 3385 | 0.324568659 | 0.50133014 | Yes |
| row_53 | HELZ2 | 3439 | 0.322200924 | 0.50723857 | Yes |
| row_54 | GBP4 | 3543 | 0.318227202 | 0.51215255 | Yes |
| row_55 | ZNFX1 | 3710 | 0.311669409 | 0.51578045 | Yes |
| row_56 | TNFAIP6 | 3882 | 0.305491179 | 0.5191856 | Yes |
| row_57 | SOCS3 | 3927 | 0.303713918 | 0.5248635 | Yes |
| row_58 | IFITM3 | 4013 | 0.30057618 | 0.5297284 | Yes |
| row_59 | PARP12 | 4296 | 0.290951222 | 0.5308039 | Yes |
| row_60 | NLRC5 | 4354 | 0.288804114 | 0.53592724 | Yes |
| row_61 | STAT2 | 4373 | 0.288193703 | 0.5417471 | Yes |
| row_62 | SELP | 4390 | 0.287442803 | 0.5475873 | Yes |
| row_63 | CCL7 | 4487 | 0.284185499 | 0.5519025 | Yes |
| row_64 | PDE4B | 4557 | 0.281886548 | 0.5566599 | Yes |
| row_65 | PARP14 | 4641 | 0.279092044 | 0.561103 | Yes |
| row_66 | IRF9 | 4732 | 0.276325196 | 0.5653597 | Yes |
| row_67 | MX2 | 4855 | 0.272225589 | 0.5689468 | Yes |
| row_68 | LY6E | 4917 | 0.270430803 | 0.5736054 | Yes |
| row_69 | PELI1 | 5069 | 0.266441047 | 0.5765415 | Yes |
| row_70 | IFITM2 | 5272 | 0.260588706 | 0.5784248 | Yes |
| row_71 | TDRD7 | 5327 | 0.259143919 | 0.58297 | Yes |
| row_72 | ARID5B | 5350 | 0.258606404 | 0.58808595 | Yes |
| row_73 | BTG1 | 5861 | 0.245194495 | 0.5840373 | Yes |
| row_74 | CXCL10 | 5946 | 0.243371934 | 0.58770025 | Yes |
| row_75 | HLA-B | 5952 | 0.243282139 | 0.59279865 | Yes |
| row_76 | VCAM1 | 6197 | 0.237019673 | 0.5934151 | Yes |
| row_77 | SAMHD1 | 6329 | 0.234061584 | 0.5960244 | Yes |
| row_78 | CXCL9 | 6503 | 0.229840338 | 0.59777945 | Yes |
| row_79 | OAS3 | 6578 | 0.228199914 | 0.6013008 | Yes |
| row_80 | FAS | 6845 | 0.222143665 | 0.6011997 | Yes |
| row_81 | HLA-A | 7005 | 0.218653947 | 0.60297084 | Yes |
| row_82 | STAT4 | 7041 | 0.217806488 | 0.60697997 | Yes |
| row_83 | MVP | 7091 | 0.216868728 | 0.61071444 | Yes |
| row_84 | PML | 7145 | 0.215783015 | 0.61435294 | Yes |
| row_85 | PIM1 | 7447 | 0.208632007 | 0.61332685 | Yes |
| row_86 | ISG20 | 7709 | 0.203341648 | 0.61291564 | Yes |
| row_87 | UPP1 | 7721 | 0.203166768 | 0.61704916 | Yes |
| row_88 | PSMB8 | 7828 | 0.200688228 | 0.6194014 | Yes |
| row_89 | SLAMF7 | 7844 | 0.200366989 | 0.6234024 | Yes |
| row_90 | VAMP5 | 8139 | 0.194255173 | 0.62219703 | Yes |
| row_91 | CD38 | 8302 | 0.191570505 | 0.62333596 | Yes |
| row_92 | CD274 | 8407 | 0.189524069 | 0.62548643 | Yes |
| row_93 | PLSCR1 | 8783 | 0.182607144 | 0.6225589 | No |
| row_94 | PFKP | 9319 | 0.1731686 | 0.61651903 | No |
| row_95 | IFI35 | 9550 | 0.16936022 | 0.615947 | No |
| row_96 | RTP4 | 9696 | 0.166970938 | 0.61687046 | No |
| row_97 | TAP1 | 9791 | 0.16524744 | 0.61868507 | No |
| row_98 | SP110 | 9897 | 0.163384989 | 0.6202598 | No |
| row_99 | CXCL11 | 10429 | 0.154840037 | 0.6139018 | No |
| row_100 | EIF2AK2 | 10462 | 0.154262349 | 0.61661005 | No |
| row_101 | DHX58 | 10493 | 0.153662786 | 0.61934197 | No |
| row_102 | IRF4 | 10765 | 0.14969793 | 0.6176046 | No |
| row_103 | OGFR | 10948 | 0.147321671 | 0.61743575 | No |
| row_104 | LGALS3BP | 11128 | 0.144522384 | 0.6172618 | No |
| row_105 | STAT1 | 11131 | 0.144475266 | 0.6203072 | No |
| row_106 | EPSTI1 | 11316 | 0.14147374 | 0.61997724 | No |
| row_107 | B2M | 11733 | 0.136008546 | 0.6153098 | No |
| row_108 | CASP1 | 11871 | 0.134068295 | 0.615677 | No |
| row_109 | CSF2RB | 12131 | 0.13050954 | 0.61374867 | No |
| row_110 | TRIM21 | 12303 | 0.128272355 | 0.61337364 | No |
| row_111 | ST8SIA4 | 12326 | 0.127895743 | 0.61570144 | No |
| row_112 | NOD1 | 12331 | 0.127863377 | 0.61835605 | No |
| row_113 | IL4R | 12604 | 0.124400839 | 0.6160609 | No |
| row_114 | ADAR | 12918 | 0.1206339 | 0.6129394 | No |
| row_115 | EIF4E3 | 13367 | 0.115088753 | 0.60724354 | No |
| row_116 | IL15RA | 13718 | 0.11122372 | 0.60324824 | No |
| row_117 | UBE2L6 | 14023 | 0.107677944 | 0.60001415 | No |
| row_118 | FPR1 | 14066 | 0.10724251 | 0.6015375 | No |
| row_119 | TOR1B | 14201 | 0.105722092 | 0.60135466 | No |
| row_120 | LATS2 | 14952 | 0.097946428 | 0.5897986 | No |
| row_121 | BPGM | 15074 | 0.096540436 | 0.5896565 | No |
| row_122 | PSMB9 | 15517 | 0.09160576 | 0.5835688 | No |
| row_123 | IL7 | 16100 | 0.086073369 | 0.57481605 | No |
| row_124 | TRIM25 | 16554 | 0.081729271 | 0.56831765 | No |
| row_125 | XCL1 | 16603 | 0.081269763 | 0.56917787 | No |
| row_126 | IFIH1 | 16757 | 0.079641484 | 0.568093 | No |
| row_127 | IRF5 | 16864 | 0.078655601 | 0.56784225 | No |
| row_128 | VAMP8 | 17347 | 0.074084431 | 0.5606531 | No |
| row_129 | CASP4 | 17760 | 0.070298001 | 0.5546568 | No |
| row_130 | SLC25A28 | 18001 | 0.068193406 | 0.5517449 | No |
| row_131 | RBCK1 | 18056 | 0.067734651 | 0.55220723 | No |
| row_132 | CMTR1 | 18368 | 0.065148145 | 0.54793864 | No |
| row_133 | P2RY14 | 18661 | 0.062715739 | 0.54396385 | No |
| row_134 | KLRK1 | 18719 | 0.062333543 | 0.5442564 | No |
| row_135 | CFB | 18803 | 0.061555121 | 0.54405934 | No |
| row_136 | AUTS2 | 18890 | 0.060843632 | 0.5437925 | No |
| row_137 | CASP8 | 19112 | 0.059042972 | 0.54103106 | No |
| row_138 | LAP3 | 19121 | 0.058963355 | 0.5421433 | No |
| row_139 | PTPN1 | 19158 | 0.058651716 | 0.54273933 | No |
| row_140 | GPR18 | 19819 | 0.053771608 | 0.5318785 | No |
| row_141 | SECTM1 | 19909 | 0.053092882 | 0.53139174 | No |
| row_142 | TAPBP | 20274 | 0.050276879 | 0.52584165 | No |
| row_143 | SOD2 | 20480 | 0.048680738 | 0.5231503 | No |
| row_144 | LYSMD2 | 20748 | 0.046557926 | 0.5192857 | No |
| row_145 | HLA-G | 21929 | 0.037586257 | 0.4986188 | No |
| row_146 | CASP7 | 22331 | 0.03497396 | 0.49206915 | No |
| row_147 | TRAFD1 | 23962 | 0.024550932 | 0.46293706 | No |
| row_148 | IFNAR2 | 24466 | 0.021488003 | 0.45424396 | No |
| row_149 | PSMB10 | 25059 | 0.018183596 | 0.44386116 | No |
| row_150 | NFKBIA | 25217 | 0.017331703 | 0.44137442 | No |
| row_151 | PTPN6 | 26288 | 0.011620062 | 0.422155 | No |
| row_152 | PSMB2 | 26878 | 0.008596419 | 0.41162226 | No |
| row_153 | STAT3 | 27240 | 0.006894953 | 0.4052014 | No |
| row_154 | MT2A | 27509 | 0.005484953 | 0.40044248 | No |
| row_155 | IRF2 | 27674 | 0.004621073 | 0.3975573 | No |
| row_156 | NMI | 28482 | 0.001029328 | 0.3828969 | No |
| row_157 | IRF1 | 31541 | -0.001104974 | 0.32728404 | No |
| row_158 | CIITA | 31721 | -0.001894287 | 0.32406777 | No |
| row_159 | PSME1 | 32379 | -0.004784517 | 0.31221655 | No |
| row_160 | NFKB1 | 33906 | -0.011833254 | 0.28470534 | No |
| row_161 | PSMA2 | 34634 | -0.015246223 | 0.2718037 | No |
| row_162 | ARL4A | 35687 | -0.020654501 | 0.25310448 | No |
| row_163 | SPPL2A | 35859 | -0.021470016 | 0.25045133 | No |
| row_164 | RNF31 | 36520 | -0.024840925 | 0.23897332 | No |
| row_165 | PSMA3 | 36733 | -0.025928516 | 0.23566933 | No |
| row_166 | CD40 | 36919 | -0.027009262 | 0.23287961 | No |
| row_167 | HLA-DRB1 | 38661 | -0.037032519 | 0.20199426 | No |
| row_168 | CD74 | 40220 | -0.047136471 | 0.17465387 | No |
| row_169 | RAPGEF6 | 41234 | -0.054297566 | 0.15738182 | No |
| row_170 | PSME2 | 41553 | -0.056752238 | 0.15280676 | No |
| row_171 | GBP6 | 42838 | -0.067382082 | 0.1308833 | No |
| row_172 | TNFAIP2 | 43280 | -0.071066238 | 0.12437576 | No |
| row_173 | IFI30 | 43945 | -0.077624992 | 0.1139509 | No |
| row_174 | IRF8 | 44019 | -0.07827305 | 0.11429237 | No |
| row_175 | ICAM1 | 44164 | -0.079841651 | 0.11337553 | No |
| row_176 | JAK2 | 44567 | -0.084138714 | 0.10785637 | No |
| row_177 | NAMPT | 44605 | -0.084525488 | 0.10898618 | No |
| row_178 | MYD88 | 45324 | -0.092234813 | 0.097890496 | No |
| row_179 | ISOC1 | 45702 | -0.096699722 | 0.09309411 | No |
| row_180 | RIPK1 | 46733 | -0.109299421 | 0.07668599 | No |
| row_181 | APOL6 | 47650 | -0.122348979 | 0.062630296 | No |
| row_182 | GCH1 | 48051 | -0.12815325 | 0.05808638 | No |
| row_183 | CASP3 | 48139 | -0.129341125 | 0.059262436 | No |
| row_184 | NCOA3 | 48258 | -0.13098754 | 0.05990961 | No |
| row_185 | PNP | 49044 | -0.143689305 | 0.048692495 | No |
| row_186 | PNPT1 | 49224 | -0.147515506 | 0.048582397 | No |
| row_187 | HLA-DMA | 49398 | -0.150774717 | 0.048650984 | No |
| row_188 | RIPK2 | 49437 | -0.151579335 | 0.051192883 | No |
| row_189 | TRIM26 | 50596 | -0.177651256 | 0.03391394 | No |
| row_190 | NUP93 | 50836 | -0.18400842 | 0.03349063 | No |
| row_191 | BANK1 | 51917 | -0.218662694 | 0.01850559 | No |
| row_192 | PTPN2 | 52278 | -0.232347459 | 0.016911937 | No |
| row_193 | HIF1A | 52758 | -0.254959285 | 0.013635551 | No |
| row_194 | ST3GAL5 | 53270 | -0.285356134 | 0.010425345 | No |
| row_195 | MTHFD2 | 54796 | -0.555831432 | -0.005463925 | No |
| row_196 | PLA2G4A | 54816 | -0.56575948 | 0.006258327 | No |

EAC Allograft Rejection

| NAME | GENE SYMBOL | RANK IN GENE LIST | RANK METRIC SCORE | RUNNING ES | CORE ENRICHMENT |
| --- | --- | --- | --- | --- | --- |
| row_0 | CCL11 | 7 | 1.304182053 | 0.02739825 | Yes |
| row_1 | CCL2 | 150 | 0.796279073 | 0.041620813 | Yes |
| row_2 | PRKCG | 152 | 0.79132098 | 0.058303956 | Yes |
| row_3 | IL1B | 242 | 0.7179178 | 0.07183689 | Yes |
| row_4 | IL11 | 250 | 0.716010809 | 0.08682141 | Yes |
| row_5 | CTSS | 267 | 0.705931425 | 0.101429455 | Yes |
| row_6 | CXCR3 | 269 | 0.703541279 | 0.116259955 | Yes |
| row_7 | MMP9 | 296 | 0.683876336 | 0.13022058 | Yes |
| row_8 | GZMA | 330 | 0.663776398 | 0.14362964 | Yes |
| row_9 | PRF1 | 468 | 0.610574186 | 0.15402375 | Yes |
| row_10 | C2 | 509 | 0.595931649 | 0.16587354 | Yes |
| row_11 | TIMP1 | 513 | 0.595271826 | 0.17838256 | Yes |
| row_12 | CD8B | 582 | 0.577799916 | 0.18934026 | Yes |
| row_13 | TLR6 | 607 | 0.571605206 | 0.20096773 | Yes |
| row_14 | CCL5 | 663 | 0.560400188 | 0.21179472 | Yes |
| row_15 | GBP2 | 825 | 0.528492749 | 0.22001982 | Yes |
| row_16 | CCND2 | 845 | 0.525075018 | 0.2307562 | Yes |
| row_17 | FLNA | 917 | 0.512500823 | 0.24028115 | Yes |
| row_18 | IL2RB | 937 | 0.510025561 | 0.25069988 | Yes |
| row_19 | IRF7 | 1041 | 0.495287418 | 0.25927937 | Yes |
| row_20 | CD8A | 1043 | 0.495170981 | 0.26971206 | Yes |
| row_21 | BCAT1 | 1107 | 0.484956801 | 0.27880123 | Yes |
| row_22 | LTB | 1138 | 0.481694341 | 0.2884219 | Yes |
| row_23 | IL10 | 1185 | 0.476363957 | 0.29763898 | Yes |
| row_24 | IL6 | 1199 | 0.475316435 | 0.30743435 | Yes |
| row_25 | CCR1 | 1313 | 0.461205095 | 0.31511256 | Yes |
| row_26 | FASLG | 1324 | 0.459390908 | 0.32462636 | Yes |
| row_27 | TLR1 | 1334 | 0.458258629 | 0.33413446 | Yes |
| row_28 | SOCS1 | 1346 | 0.45701012 | 0.34357983 | Yes |
| row_29 | LY86 | 1381 | 0.453553885 | 0.35253382 | Yes |
| row_30 | GCNT1 | 1585 | 0.43593505 | 0.35804132 | Yes |
| row_31 | CCL4 | 1773 | 0.419379324 | 0.3634905 | Yes |
| row_32 | STAB1 | 1883 | 0.41057688 | 0.37017295 | Yes |
| row_33 | CD3E | 1899 | 0.409382284 | 0.3785403 | Yes |
| row_34 | CD2 | 1982 | 0.403371841 | 0.3855619 | Yes |
| row_35 | EGFR | 2000 | 0.401850343 | 0.39373392 | Yes |
| row_36 | SPI1 | 2089 | 0.395433486 | 0.4004788 | Yes |
| row_37 | ZAP70 | 2267 | 0.383050323 | 0.40534317 | Yes |
| row_38 | KLRD1 | 2296 | 0.381415993 | 0.4128838 | Yes |
| row_39 | IL15 | 2386 | 0.375227123 | 0.41918403 | Yes |
| row_40 | FGR | 2444 | 0.371225744 | 0.42598197 | Yes |
| row_41 | CD3D | 2485 | 0.368796229 | 0.43303794 | Yes |
| row_42 | CCR5 | 2526 | 0.365841508 | 0.44003153 | Yes |
| row_43 | ITGAL | 2592 | 0.362424821 | 0.4464982 | Yes |
| row_44 | LCK | 2614 | 0.361372232 | 0.45374313 | Yes |
| row_45 | ITGB2 | 2620 | 0.36099264 | 0.46127114 | Yes |
| row_46 | IL2RG | 2669 | 0.358765721 | 0.46796986 | Yes |
| row_47 | IL12RB1 | 2716 | 0.356109142 | 0.4746489 | Yes |
| row_48 | EREG | 2778 | 0.353402793 | 0.4809979 | Yes |
| row_49 | LCP2 | 2851 | 0.350116283 | 0.48707744 | Yes |
| row_50 | CD4 | 2879 | 0.348263234 | 0.49393654 | Yes |
| row_51 | NLRP3 | 2932 | 0.345734656 | 0.5002875 | Yes |
| row_52 | HLA-DQA1 | 3101 | 0.33672151 | 0.5043377 | Yes |
| row_53 | GPR65 | 3149 | 0.334863991 | 0.5105502 | Yes |
| row_54 | CD86 | 3279 | 0.328740329 | 0.51514155 | Yes |
| row_55 | F2R | 3280 | 0.32866776 | 0.5220783 | Yes |
| row_56 | CD7 | 3303 | 0.327435344 | 0.5285888 | Yes |
| row_57 | PF4 | 3450 | 0.321768224 | 0.5327237 | Yes |
| row_58 | NCF4 | 3464 | 0.321255267 | 0.53926754 | Yes |
| row_59 | NCR1 | 3469 | 0.321124941 | 0.5459723 | Yes |
| row_60 | CCR2 | 3522 | 0.318939537 | 0.5517577 | Yes |
| row_61 | PRKCB | 3614 | 0.315528959 | 0.55676156 | Yes |
| row_62 | THY1 | 3643 | 0.31413883 | 0.56288224 | Yes |
| row_63 | CD96 | 3937 | 0.30336225 | 0.56395435 | Yes |
| row_64 | IGSF6 | 4134 | 0.296405017 | 0.5666443 | Yes |
| row_65 | DYRK3 | 4145 | 0.296125054 | 0.5727123 | Yes |
| row_66 | SRGN | 4246 | 0.292773217 | 0.57707214 | Yes |
| row_67 | GZMB | 4291 | 0.291130751 | 0.5824162 | Yes |
| row_68 | CD28 | 4292 | 0.291125655 | 0.5885605 | Yes |
| row_69 | CCL7 | 4487 | 0.284185499 | 0.591029 | Yes |
| row_70 | CFP | 4593 | 0.280638754 | 0.5950418 | Yes |
| row_71 | INHBA | 4626 | 0.279546469 | 0.6003596 | Yes |
| row_72 | PTPRC | 4740 | 0.276065916 | 0.6041304 | Yes |
| row_73 | FCGR2B | 5379 | 0.257870734 | 0.5979657 | Yes |
| row_74 | CD80 | 5440 | 0.256078035 | 0.6022788 | Yes |
| row_75 | F2 | 5537 | 0.253494263 | 0.60588247 | Yes |
| row_76 | FYB1 | 5694 | 0.249076992 | 0.6083013 | Yes |
| row_77 | ITK | 5800 | 0.246496007 | 0.6115934 | Yes |
| row_78 | HLA-E | 6398 | 0.232651442 | 0.60564244 | Yes |
| row_79 | CXCL9 | 6503 | 0.229840338 | 0.6086013 | Yes |
| row_80 | CD40LG | 6778 | 0.223953784 | 0.60834306 | Yes |
| row_81 | LY75 | 6809 | 0.223218977 | 0.6125085 | Yes |
| row_82 | FAS | 6845 | 0.222143665 | 0.6165602 | Yes |
| row_83 | SIT1 | 6944 | 0.220000118 | 0.6194205 | Yes |
| row_84 | ETS1 | 6955 | 0.219691768 | 0.6238753 | Yes |
| row_85 | HLA-A | 7005 | 0.218653947 | 0.6275987 | Yes |
| row_86 | STAT4 | 7041 | 0.217806488 | 0.6315589 | Yes |
| row_87 | CSF1 | 7944 | 0.198425531 | 0.61933666 | No |
| row_88 | UBE2D1 | 7975 | 0.197684377 | 0.6229631 | No |
| row_89 | CD79A | 8024 | 0.196509331 | 0.6262373 | No |
| row_90 | IL18RAP | 8678 | 0.184662446 | 0.61825466 | No |
| row_91 | IL16 | 8724 | 0.183783069 | 0.6213148 | No |
| row_92 | HLA-DOB | 9022 | 0.178298086 | 0.61967456 | No |
| row_93 | IKBKB | 9244 | 0.174469456 | 0.6193362 | No |
| row_94 | TAP1 | 9791 | 0.16524744 | 0.6128904 | No |
| row_95 | HLA-DOA | 9839 | 0.164413542 | 0.6155054 | No |
| row_96 | IFNGR1 | 9970 | 0.162282154 | 0.6165654 | No |
| row_97 | CD1D | 10205 | 0.158279806 | 0.6156488 | No |
| row_98 | CD247 | 10220 | 0.158024237 | 0.6187293 | No |
| row_99 | WAS | 10467 | 0.154208794 | 0.6175085 | No |
| row_100 | CD3G | 10570 | 0.152597487 | 0.6188735 | No |
| row_101 | DEGS1 | 10599 | 0.152102739 | 0.6215743 | No |
| row_102 | IRF4 | 10765 | 0.14969793 | 0.6217319 | No |
| row_103 | STAT1 | 11131 | 0.144475266 | 0.61814064 | No |
| row_104 | CARTPT | 11474 | 0.139216512 | 0.6148569 | No |
| row_105 | MAP3K7 | 11623 | 0.1374152 | 0.61506456 | No |
| row_106 | B2M | 11733 | 0.136008546 | 0.6159521 | No |
| row_107 | CRTAM | 12223 | 0.129230514 | 0.6097831 | No |
| row_108 | ACVR2A | 12250 | 0.128977194 | 0.6120323 | No |
| row_109 | ST8SIA4 | 12326 | 0.127895743 | 0.6133671 | No |
| row_110 | ACHE | 12473 | 0.126131147 | 0.613373 | No |
| row_111 | ABI1 | 12572 | 0.12485452 | 0.6142252 | No |
| row_112 | IL4R | 12604 | 0.124400839 | 0.6162868 | No |
| row_113 | EIF4G3 | 12607 | 0.124380045 | 0.6188755 | No |
| row_114 | HLA-DRA | 14350 | 0.10415718 | 0.5893815 | No |
| row_115 | TRAT1 | 14571 | 0.10171514 | 0.5875258 | No |
| row_116 | IL2 | 15075 | 0.096540406 | 0.5804122 | No |
| row_117 | HCLS1 | 15540 | 0.091386646 | 0.57389945 | No |
| row_118 | IL12B | 16057 | 0.086545408 | 0.5663384 | No |
| row_119 | IL7 | 16100 | 0.086073369 | 0.5673909 | No |
| row_120 | ELF4 | 16722 | 0.080045171 | 0.5577824 | No |
| row_121 | TLR3 | 16839 | 0.078894094 | 0.55733716 | No |
| row_122 | IL2RA | 17162 | 0.075873129 | 0.5530803 | No |
| row_123 | TRAF2 | 17208 | 0.075477183 | 0.55385464 | No |
| row_124 | LIF | 17301 | 0.074515551 | 0.55375355 | No |
| row_125 | CCL19 | 17418 | 0.073365189 | 0.5531916 | No |
| row_126 | TGFB2 | 18182 | 0.066718534 | 0.54071844 | No |
| row_127 | CCL13 | 18275 | 0.06594196 | 0.54043645 | No |
| row_128 | IL4 | 19484 | 0.056143124 | 0.51964414 | No |
| row_129 | ELANE | 19749 | 0.054318152 | 0.5159876 | No |
| row_130 | CCND3 | 20036 | 0.052270923 | 0.5118876 | No |
| row_131 | TAPBP | 20274 | 0.050276879 | 0.50863695 | No |
| row_132 | HLA-G | 21929 | 0.037586257 | 0.4793389 | No |
| row_133 | KRT1 | 22622 | 0.033048511 | 0.4674468 | No |
| row_134 | APBB1 | 23353 | 0.028319852 | 0.4547636 | No |
| row_135 | RPS19 | 23584 | 0.026947465 | 0.4511479 | No |
| row_136 | NCK1 | 24002 | 0.024318768 | 0.4440747 | No |
| row_137 | IFNG | 24038 | 0.024142545 | 0.44394746 | No |
| row_138 | TAP2 | 24259 | 0.02259388 | 0.44042185 | No |
| row_139 | IFNAR2 | 24466 | 0.021488003 | 0.4371276 | No |
| row_140 | PSMB10 | 25059 | 0.018183596 | 0.42674106 | No |
| row_141 | MBL2 | 25353 | 0.016578803 | 0.4217604 | No |
| row_142 | PTPN6 | 26288 | 0.011620062 | 0.40501335 | No |
| row_143 | HLA-DMB | 26766 | 0.009169032 | 0.39652878 | No |
| row_144 | ICOSLG | 27675 | 0.004615273 | 0.38010687 | No |
| row_145 | CD47 | 31757 | -0.002068444 | 0.30590463 | No |
| row_146 | EIF3A | 33944 | -0.012053021 | 0.26638898 | No |
| row_147 | ABCE1 | 34540 | -0.014817954 | 0.25587684 | No |
| row_148 | MAP4K1 | 34789 | -0.015888065 | 0.2517003 | No |
| row_149 | RPL3L | 35702 | -0.020729465 | 0.23554574 | No |
| row_150 | IL13 | 36131 | -0.022861732 | 0.22824161 | No |
| row_151 | RPS3A | 36308 | -0.023728855 | 0.22554044 | No |
| row_152 | UBE2N | 36396 | -0.024270725 | 0.2244699 | No |
| row_153 | GALNT1 | 36585 | -0.025126765 | 0.22157992 | No |
| row_154 | CD40 | 36919 | -0.027009262 | 0.21609168 | No |
| row_155 | SOCS5 | 37066 | -0.027787382 | 0.21402197 | No |
| row_156 | IL9 | 37615 | -0.031000741 | 0.20470645 | No |
| row_157 | EIF3J | 38100 | -0.033818785 | 0.19661477 | No |
| row_158 | TPD52 | 39440 | -0.042078894 | 0.17314236 | No |
| row_159 | BRCA1 | 39833 | -0.044701047 | 0.16695413 | No |
| row_160 | RPS9 | 39991 | -0.045642041 | 0.16506112 | No |
| row_161 | CAPG | 40031 | -0.045896586 | 0.16532026 | No |
| row_162 | CD74 | 40220 | -0.047136471 | 0.16289482 | No |
| row_163 | LYN | 41756 | -0.058267415 | 0.13619824 | No |
| row_164 | CCL22 | 42652 | -0.065825932 | 0.12130474 | No |
| row_165 | TLR2 | 42706 | -0.066240944 | 0.12173857 | No |
| row_166 | EIF5A | 42766 | -0.066758484 | 0.12207416 | No |
| row_167 | BCL10 | 43457 | -0.072838396 | 0.11105824 | No |
| row_168 | BCL3 | 43921 | -0.077358268 | 0.104267545 | No |
| row_169 | IRF8 | 44019 | -0.07827305 | 0.104154825 | No |
| row_170 | ICAM1 | 44164 | -0.079841651 | 0.103220135 | No |
| row_171 | JAK2 | 44567 | -0.084138714 | 0.09768232 | No |
| row_172 | IFNGR2 | 44723 | -0.085651129 | 0.09667012 | No |
| row_173 | CSK | 44798 | -0.086337827 | 0.09714605 | No |
| row_174 | GLMN | 46637 | -0.108098842 | 0.06598869 | No |
| row_175 | EIF3D | 46808 | -0.110174321 | 0.06522117 | No |
| row_176 | INHBB | 47070 | -0.113891222 | 0.06287653 | No |
| row_177 | IL12A | 47346 | -0.118009061 | 0.06036409 | No |
| row_178 | MRPL3 | 48216 | -0.13031736 | 0.04730475 | No |
| row_179 | RPL39 | 48371 | -0.132713899 | 0.04730403 | No |
| row_180 | HLA-DMA | 49398 | -0.150774717 | 0.03182014 | No |
| row_181 | RIPK2 | 49437 | -0.151579335 | 0.034327984 | No |
| row_182 | MTIF2 | 50662 | -0.179184437 | 0.015841477 | No |
| row_183 | IL18 | 51000 | -0.188679829 | 0.013692623 | No |
| row_184 | HDAC9 | 51198 | -0.194526419 | 0.014214194 | No |
| row_185 | RPL9 | 51310 | -0.197925746 | 0.016372114 | No |
| row_186 | AKT1 | 51989 | -0.221300229 | 0.0087079 | No |
| row_187 | CXCL13 | 52127 | -0.225886002 | 0.01098292 | No |
| row_188 | TNF | 52251 | -0.231384158 | 0.013628684 | No |
| row_189 | NPM1 | 52592 | -0.246517494 | 0.012645952 | No |
| row_190 | HIF1A | 52758 | -0.254959285 | 0.015025175 | No |
| row_191 | NME1 | 52992 | -0.267384678 | 0.016429517 | No |
| row_192 | IL27RA | 53075 | -0.272531986 | 0.020689648 | No |
| row_193 | NOS2 | 53993 | -0.356202245 | 0.011524485 | No |
| row_194 | CDKN2A | 54561 | -0.459026843 | 0.010897074 | No |

EAC EMT

| NAME | GENE SYMBOL | RANK IN GENE LIST | RANK METRIC SCORE | RUNNING ES | CORE ENRICHMENT |
| --- | --- | --- | --- | --- | --- |
| row_0 | TNFRSF11B | 8 | 1.288557529 | 0.021505008 | Yes |
| row_1 | ANPEP | 30 | 1.087220788 | 0.03939059 | Yes |
| row_2 | TGFBI | 106 | 0.875713348 | 0.052739896 | Yes |
| row_3 | SFRP4 | 143 | 0.806087077 | 0.06562891 | Yes |
| row_4 | LAMA1 | 144 | 0.80413574 | 0.079140134 | Yes |
| row_5 | GAS1 | 154 | 0.788656294 | 0.092227526 | Yes |
| row_6 | DKK1 | 185 | 0.7586689 | 0.10442898 | Yes |
| row_7 | PTHLH | 231 | 0.724836767 | 0.115789056 | Yes |
| row_8 | COL3A1 | 360 | 0.651149333 | 0.1244009 | Yes |
| row_9 | LRRC15 | 372 | 0.646998584 | 0.13507174 | Yes |
| row_10 | MMP1 | 465 | 0.610984743 | 0.14366372 | Yes |
| row_11 | TIMP1 | 513 | 0.595271826 | 0.15281044 | Yes |
| row_12 | FBLN1 | 548 | 0.587379277 | 0.16206108 | Yes |
| row_13 | CDH11 | 637 | 0.56507957 | 0.16995454 | Yes |
| row_14 | NT5E | 717 | 0.548047602 | 0.17772557 | Yes |
| row_15 | DCN | 736 | 0.54482758 | 0.18655236 | Yes |
| row_16 | PMP22 | 745 | 0.542446494 | 0.19552107 | Yes |
| row_17 | GREM1 | 746 | 0.541993678 | 0.20462775 | Yes |
| row_18 | DAB2 | 749 | 0.541579843 | 0.21369107 | Yes |
| row_19 | IGFBP3 | 790 | 0.534749806 | 0.22194825 | Yes |
| row_20 | CXCL12 | 809 | 0.531612575 | 0.230553 | Yes |
| row_21 | TFPI2 | 815 | 0.530196011 | 0.23937048 | Yes |
| row_22 | POSTN | 897 | 0.517834723 | 0.24659747 | Yes |
| row_23 | FLNA | 917 | 0.512500823 | 0.2548629 | Yes |
| row_24 | RGS4 | 934 | 0.510213971 | 0.2631445 | Yes |
| row_25 | MXRA5 | 940 | 0.509274006 | 0.27161044 | Yes |
| row_26 | TNC | 967 | 0.504122376 | 0.27960774 | Yes |
| row_27 | PRRX1 | 985 | 0.501371443 | 0.28772256 | Yes |
| row_28 | LOX | 1016 | 0.498365402 | 0.29555035 | Yes |
| row_29 | ABI3BP | 1024 | 0.497919887 | 0.3037891 | Yes |
| row_30 | LAMA3 | 1067 | 0.490747005 | 0.31127056 | Yes |
| row_31 | SGCD | 1077 | 0.489486903 | 0.31933126 | Yes |
| row_32 | WIPF1 | 1081 | 0.488650292 | 0.32748705 | Yes |
| row_33 | CADM1 | 1168 | 0.477437854 | 0.33394432 | Yes |
| row_34 | SPOCK1 | 1171 | 0.477220327 | 0.34192628 | Yes |
| row_35 | IL6 | 1199 | 0.475316435 | 0.34942138 | Yes |
| row_36 | COL6A3 | 1205 | 0.474331379 | 0.3573002 | Yes |
| row_37 | SPP1 | 1214 | 0.47303462 | 0.36510265 | Yes |
| row_38 | CCN1 | 1215 | 0.472770184 | 0.37304622 | Yes |
| row_39 | BGN | 1238 | 0.469448388 | 0.3805337 | Yes |
| row_40 | GPX7 | 1294 | 0.462990731 | 0.38731226 | Yes |
| row_41 | SERPINE2 | 1437 | 0.448335975 | 0.39226165 | Yes |
| row_42 | NID2 | 1467 | 0.44596082 | 0.39922714 | Yes |
| row_43 | LUM | 1522 | 0.440733761 | 0.40564993 | Yes |
| row_44 | COL1A1 | 1572 | 0.436851025 | 0.41209844 | Yes |
| row_45 | PCOLCE | 1611 | 0.433571577 | 0.418692 | Yes |
| row_46 | ECM2 | 1627 | 0.431884617 | 0.4256757 | Yes |
| row_47 | FBLN2 | 1650 | 0.430256307 | 0.43250465 | Yes |
| row_48 | SNAI2 | 1658 | 0.429469705 | 0.43959332 | Yes |
| row_49 | COL5A2 | 1712 | 0.425439626 | 0.44577733 | Yes |
| row_50 | COL5A1 | 1812 | 0.415816933 | 0.4509627 | Yes |
| row_51 | PDGFRB | 1828 | 0.414635718 | 0.45765656 | Yes |
| row_52 | TNFAIP3 | 1854 | 0.413203329 | 0.4641444 | Yes |
| row_53 | SLIT3 | 1862 | 0.412570685 | 0.47094914 | Yes |
| row_54 | FAP | 1898 | 0.409386218 | 0.4771909 | Yes |
| row_55 | MMP2 | 1932 | 0.407551557 | 0.48343825 | Yes |
| row_56 | MGP | 2016 | 0.400838614 | 0.48866308 | Yes |
| row_57 | MSX1 | 2065 | 0.397326201 | 0.49446568 | Yes |
| row_58 | MYLK | 2071 | 0.396962225 | 0.5010445 | Yes |
| row_59 | ADAM12 | 2207 | 0.387433529 | 0.505098 | Yes |
| row_60 | ECM1 | 2236 | 0.385573536 | 0.51106703 | Yes |
| row_61 | COL16A1 | 2271 | 0.382730991 | 0.51687914 | Yes |
| row_62 | IL15 | 2386 | 0.375227123 | 0.52110964 | Yes |
| row_63 | FERMT2 | 2414 | 0.373395175 | 0.5268922 | Yes |
| row_64 | COL8A2 | 2422 | 0.372773349 | 0.53302824 | Yes |
| row_65 | FN1 | 2473 | 0.369611233 | 0.5383288 | Yes |
| row_66 | FGF2 | 2606 | 0.361613631 | 0.54200304 | Yes |
| row_67 | TIMP3 | 2652 | 0.359613448 | 0.54722655 | Yes |
| row_68 | ACTA2 | 2763 | 0.354118705 | 0.5511752 | Yes |
| row_69 | COL1A2 | 2775 | 0.353494883 | 0.5569145 | Yes |
| row_70 | FBN1 | 2788 | 0.352900535 | 0.56262565 | Yes |
| row_71 | CTHRC1 | 2798 | 0.352253497 | 0.56838053 | Yes |
| row_72 | SPARC | 2846 | 0.35032624 | 0.57341164 | Yes |
| row_73 | MMP3 | 2862 | 0.349519908 | 0.57901144 | Yes |
| row_74 | COL12A1 | 2967 | 0.343991488 | 0.582899 | Yes |
| row_75 | AREG | 2978 | 0.343336076 | 0.58848584 | Yes |
| row_76 | SLIT2 | 3034 | 0.340514451 | 0.5932065 | Yes |
| row_77 | CCN2 | 3068 | 0.33835867 | 0.5982913 | Yes |
| row_78 | EDIL3 | 3127 | 0.335552454 | 0.60287404 | Yes |
| row_79 | PLOD2 | 3224 | 0.33136791 | 0.60669506 | Yes |
| row_80 | FZD8 | 3467 | 0.321229309 | 0.6076894 | Yes |
| row_81 | BMP1 | 3490 | 0.320192605 | 0.612669 | Yes |
| row_82 | DST | 3584 | 0.316400737 | 0.61629313 | Yes |
| row_83 | THY1 | 3643 | 0.31413883 | 0.6205161 | Yes |
| row_84 | GLIPR1 | 3760 | 0.309601337 | 0.6236075 | Yes |
| row_85 | LAMA2 | 3924 | 0.303863972 | 0.6257474 | Yes |
| row_86 | TGM2 | 3961 | 0.302756488 | 0.6301794 | Yes |
| row_87 | PTX3 | 4188 | 0.294710875 | 0.63101923 | Yes |
| row_88 | VCAN | 4189 | 0.294687241 | 0.6359706 | Yes |
| row_89 | FSTL1 | 4301 | 0.290890664 | 0.63883865 | Yes |
| row_90 | MYL9 | 4339 | 0.2895495 | 0.6430305 | Yes |
| row_91 | EFEMP2 | 4379 | 0.288008571 | 0.6471601 | Yes |
| row_92 | DPYSL3 | 4514 | 0.283370256 | 0.64948326 | Yes |
| row_93 | INHBA | 4626 | 0.279546469 | 0.6521607 | Yes |
| row_94 | GEM | 4669 | 0.278440326 | 0.65607494 | Yes |
| row_95 | NTM | 4786 | 0.27441588 | 0.6585752 | Yes |
| row_96 | COL6A2 | 4825 | 0.273238271 | 0.66247475 | Yes |
| row_97 | FBLN5 | 4856 | 0.272133529 | 0.66650134 | Yes |
| row_98 | BASP1 | 4995 | 0.268449694 | 0.6685011 | Yes |
| row_99 | PLOD3 | 5184 | 0.262909681 | 0.66949797 | Yes |
| row_100 | COL7A1 | 5346 | 0.258651316 | 0.6709146 | Yes |
| row_101 | CALD1 | 5410 | 0.257008791 | 0.67408663 | Yes |
| row_102 | LRP1 | 5501 | 0.254392922 | 0.6767235 | Yes |
| row_103 | GPC1 | 5530 | 0.253630847 | 0.6804756 | Yes |
| row_104 | LAMC2 | 5557 | 0.252933115 | 0.6842524 | Yes |
| row_105 | COL5A3 | 5595 | 0.251614094 | 0.68780684 | Yes |
| row_106 | CDH6 | 5635 | 0.250524282 | 0.69130665 | Yes |
| row_107 | TAGLN | 5780 | 0.247008801 | 0.69283694 | Yes |
| row_108 | VCAM1 | 6197 | 0.237019673 | 0.68925047 | Yes |
| row_109 | CXCL1 | 6203 | 0.236885086 | 0.69313973 | Yes |
| row_110 | THBS1 | 6380 | 0.232925072 | 0.6938511 | Yes |
| row_111 | SCG2 | 6553 | 0.228634775 | 0.69456327 | Yes |
| row_112 | CXCL6 | 6560 | 0.228445932 | 0.6982925 | Yes |
| row_113 | COL11A1 | 6565 | 0.228371918 | 0.7020568 | Yes |
| row_114 | WNT5A | 6618 | 0.227161273 | 0.70492756 | Yes |
| row_115 | FAS | 6845 | 0.222143665 | 0.7045481 | Yes |
| row_116 | COL4A2 | 6977 | 0.219284207 | 0.7058491 | Yes |
| row_117 | COL4A1 | 7496 | 0.207853496 | 0.6999168 | No |
| row_118 | MMP14 | 7770 | 0.201845258 | 0.69834113 | No |
| row_119 | TPM2 | 7955 | 0.198120967 | 0.69832224 | No |
| row_120 | NOTCH2 | 8047 | 0.195896193 | 0.699958 | No |
| row_121 | VIM | 8345 | 0.190737337 | 0.6977591 | No |
| row_122 | VEGFC | 8479 | 0.18827562 | 0.6985027 | No |
| row_123 | LOXL2 | 8505 | 0.187782377 | 0.701203 | No |
| row_124 | MFAP5 | 8989 | 0.179026186 | 0.6954231 | No |
| row_125 | SERPINH1 | 9058 | 0.177652121 | 0.69717085 | No |
| row_126 | FUCA1 | 9552 | 0.169347569 | 0.6910464 | No |
| row_127 | SGCB | 9674 | 0.167301819 | 0.69165593 | No |
| row_128 | EMP3 | 9946 | 0.162589565 | 0.6894571 | No |
| row_129 | ITGB5 | 10184 | 0.158625931 | 0.6878103 | No |
| row_130 | IL32 | 10344 | 0.156208336 | 0.687542 | No |
| row_131 | NNMT | 10446 | 0.154601827 | 0.68830204 | No |
| row_132 | OXTR | 10665 | 0.151198477 | 0.6868761 | No |
| row_133 | PLAUR | 11629 | 0.137306258 | 0.671662 | No |
| row_134 | SGCG | 11967 | 0.132815763 | 0.66776204 | No |
| row_135 | LOXL1 | 12384 | 0.127347752 | 0.6623329 | No |
| row_136 | MAGEE1 | 12509 | 0.125654206 | 0.66218805 | No |
| row_137 | LGALS1 | 12609 | 0.124370888 | 0.66247654 | No |
| row_138 | MEST | 12701 | 0.123269878 | 0.66289204 | No |
| row_139 | ITGB3 | 13073 | 0.118772902 | 0.65813756 | No |
| row_140 | COMP | 13109 | 0.118293859 | 0.6594884 | No |
| row_141 | ELN | 13763 | 0.110723168 | 0.6494678 | No |
| row_142 | PVR | 14700 | 0.100480728 | 0.6341262 | No |
| row_143 | TPM1 | 14843 | 0.098983116 | 0.6332057 | No |
| row_144 | JUN | 15091 | 0.096437521 | 0.63033205 | No |
| row_145 | SLC6A8 | 15138 | 0.095841229 | 0.6311054 | No |
| row_146 | HTRA1 | 15317 | 0.093803763 | 0.62944293 | No |
| row_147 | IGFBP2 | 15683 | 0.090165742 | 0.624317 | No |
| row_148 | PLOD1 | 16297 | 0.084192403 | 0.6145784 | No |
| row_149 | CDH2 | 16811 | 0.079218045 | 0.6065757 | No |
| row_150 | FSTL3 | 16918 | 0.078256458 | 0.605962 | No |
| row_151 | PMEPA1 | 17064 | 0.076742403 | 0.60461324 | No |
| row_152 | P3H1 | 17139 | 0.07604485 | 0.6045446 | No |
| row_153 | FBN2 | 17851 | 0.069467008 | 0.5927756 | No |
| row_154 | APLP1 | 19352 | 0.057176251 | 0.5664447 | No |
| row_155 | THBS2 | 19776 | 0.054116689 | 0.55965775 | No |
| row_156 | GJA1 | 19925 | 0.052906316 | 0.5578539 | No |
| row_157 | PFN2 | 23047 | 0.03025387 | 0.50157756 | No |
| row_158 | ID2 | 24210 | 0.02296163 | 0.48082146 | No |
| row_159 | SDC4 | 24407 | 0.021786084 | 0.47762144 | No |
| row_160 | CAP2 | 24943 | 0.018769352 | 0.4682028 | No |
| row_161 | SERPINE1 | 27795 | 0.004097749 | 0.41639945 | No |
| row_162 | ITGAV | 28516 | 8.46E-04 | 0.4033137 | No |
| row_163 | LAMC1 | 28628 | 2.92E-04 | 0.40129903 | No |
| row_164 | COPA | 31450 | -6.01E-04 | 0.34998277 | No |
| row_165 | CALU | 31763 | -0.002098859 | 0.34434137 | No |
| row_166 | FOXC2 | 32519 | -0.005543634 | 0.33069775 | No |
| row_167 | ITGB1 | 33049 | -0.007916757 | 0.32120594 | No |
| row_168 | GADD45B | 35435 | -0.019212956 | 0.27813515 | No |
| row_169 | FMOD | 37883 | -0.032434389 | 0.23415844 | No |
| row_170 | PPIB | 38192 | -0.034313999 | 0.22913112 | No |
| row_171 | CAPG | 40031 | -0.045896586 | 0.19646099 | No |
| row_172 | TPM4 | 40417 | -0.04847651 | 0.19027066 | No |
| row_173 | ENO2 | 41007 | -0.052736785 | 0.18044026 | No |
| row_174 | CRLF1 | 41136 | -0.053627614 | 0.17901243 | No |
| row_175 | ITGA2 | 41363 | -0.055291925 | 0.17582953 | No |
| row_176 | ITGA5 | 42882 | -0.067718223 | 0.14934826 | No |
| row_177 | CXCL8 | 43124 | -0.069682911 | 0.14613424 | No |
| row_178 | CD44 | 43223 | -0.070513986 | 0.14553598 | No |
| row_179 | SFRP1 | 43295 | -0.071185224 | 0.14544024 | No |
| row_180 | MATN2 | 43620 | -0.074293181 | 0.14079355 | No |
| row_181 | TGFBR3 | 45433 | -0.09336926 | 0.10939412 | No |
| row_182 | SNTB1 | 45508 | -0.094355732 | 0.10963312 | No |
| row_183 | COLGALT1 | 45588 | -0.095342219 | 0.109797716 | No |
| row_184 | IGFBP4 | 46525 | -0.106591702 | 0.09455874 | No |
| row_185 | RHOB | 47246 | -0.116314568 | 0.08341312 | No |
| row_186 | CD59 | 47533 | -0.120577268 | 0.08023548 | No |
| row_187 | PCOLCE2 | 47689 | -0.122811541 | 0.07947885 | No |
| row_188 | GADD45A | 48081 | -0.128670797 | 0.074526794 | No |
| row_189 | TNFRSF12A | 48206 | -0.130138367 | 0.074457295 | No |
| row_190 | QSOX1 | 48209 | -0.1301907 | 0.0766084 | No |
| row_191 | MATN3 | 48743 | -0.139028534 | 0.06924677 | No |
| row_192 | PDLIM4 | 49999 | -0.162787721 | 0.049147997 | No |
| row_193 | BDNF | 50537 | -0.176433414 | 0.042342074 | No |
| row_194 | SDC1 | 50707 | -0.180803463 | 0.042305112 | No |
| row_195 | SAT1 | 52173 | -0.227632478 | 0.019475052 | No |
| row_196 | VEGFA | 53782 | -0.331015676 | -0.004219744 | No |
| row_197 | MCM7 | 54400 | -0.422869325 | -0.00834056 | No |
| row_198 | PRSS2 | 55142 | -1.318286896 | 3.27E-04 | No |
